# Modelling of a new form of nitrogen doped activated carbon for adsorption of various dyes and hexavalent chromium ions

**DOI:** 10.1038/s41598-025-87398-6

**Published:** 2025-01-31

**Authors:** Mohamed A. El-Nemr, Uyiosa Osagie Aigbe, Kingsley Eghonghon Ukhurebor, Kingsley Obodo, Adetunji Ajibola Awe, Mohamed A. Hassaan, Safaa Ragab, Ahmed El Nemr

**Affiliations:** 1 Department of Chemical Engineering, Faculty of Engineering, Minia University, Minia, Egypt; 2 Department of Mathematics and Physics, Cape Peninsula University of Technology, Cape Town, South Africa; 3 Department of Physics, Faculty of Science, Edo State University Uzairue, Edo State, Nigeria; 4 Center for Space Research, North-West University, Potchefstroom, 2531 South Africa; 5 Department of Conservation and Marine Sciences, Cape Peninsula University of Technology, Cape Town, South Africa; 6 Environment Division, National Institute of Oceanography and Fisheries, Kayet Bey, El-Anfoushy, Alexandria, Egypt

**Keywords:** N-doped activated carbon, Hexavalent Chromium, Acid Brown 14 dye, Acid Orange 7 dye, Adsorption, Dry fish waste, Environmental chemistry, Chemical engineering

## Abstract

This study reports a new form of nitrogen-doped activated carbon (AC5-600) produced from a blend of sawdust (SD) and fish waste (FW) treated with urea and ZnCl_2_ for the adsorption of toxic metals and dyes. The adsorbent was also explored in the treatment of acid brown 14 (AB14) and acid orange 7 (AO7) dye molecules and hexavalent chromium (Cr^6+^) ions. The pH controls the sorption of individual contaminants, with an observed superlative % of individual contaminants removed at pH 1.5. Removal at pH was credited to the electrostatic interaction (EI) between the anion dyes and Cr^6+^ species at this pH and the protonated sites accessible on the AC5-600 adsorbent surface. Based on the error values obtained from the non-linear modelling (NLM) of the kinetic and isotherm models, the Elovich (ELM-AB14 and Cr^6+^), pseudo-first- (PFOM-AB14) and second-order models (PSOM-AB14, AO7 and Cr^6+^) and the Freundlich (FRHM) model were found to ideally define the sorption of the various contaminants. The determined maximum sorption capacity (*Q*_m_) based on the NLM was 1114, 1929 and 318 mg.g^-1^ for AB14 dye, AO7 dye and Cr^6+^ ions, respectively. Based on the computational adsorption calculations, the sorption energies for the AO7 and AB14 dyes were -4.492 and -8.090 eV and 2.563, 1.789, 1.226 and 1.928 eV for Cr_2_, CrO_3_, CrO_4_, and CrO_4_H species. AB14 and AO7 dyes and Cr^6+^ ions adsorption to synthesised AC5-600 was predicted employing the response surface methodology (RSM) and artificial neural network (ANN) models. The ANN model was more effective in predicting AB14 and AO7 dyes and Cr^6+^ ions adsorption than the RSM, and it was highly applicable in the sorption process.

**Supplementary Information:**

The online version contains supplementary material available at 10.1038/s41598-025-87398-6.

## Introduction

The existence of noxious heavy metals (HMs) in shallow waters and wastewater epitomises a serious ecosystem and civic well-being issue. The discharge of run-offs from various industrial processes comprises of high concentrations of dissolved metals such as chromate (Cr^3+^ and Cr^6+^), which have to be reduced according to governmental standards^1^. Chromium (Cr) is a distinctive non-biodegradable HMs. It exists in trivalent Cr (Cr^3+^) and hexavalent Cr (Cr^6+^) forms. It is generally employed in different manufacturing processes like leather tanning, steelmaking, mining and electroplating. Among the Cr species, Cr^6+^ is extremely peripatetic in marine systems or soil and its noxiousness, carcinogenicity and mutagenicity are 5.0 × 10^2^ and 1.0 × 10^3^ times that of Cr^3+^ due to its high solubility and easy adsorption and build-up in the stomach, liver and kidney. It has been listed by the “Environmental-Protection-Agency (EPA) of the United States (US)” as one of the 17 chemicals that pose a key hazard to people. The permissible limit of Cr^6+^ according to the “World-Health-Organization (WHO)” and EPA of the US are 0.05 and 0.1 mg L^–1^. Hence, it is essential to find an effective, eco-friendly, cost-effective, modest and achievable approach to remediate wastewater containing Cr^6+^ ions^2,3^.

It is projected that over 7.0 × 10^5^ tonnes of roughly 1.0 × 10^4^ various dyes (carbon-based organic composites), as well as pigments (inorganic composites), are manufactured globally. A huge portion of these artificial dyes is generally employed in paper, leather, printing, food, textile and cosmetic industries. It is projected that 50–100 L of water per kilogram (kg) of product is utilized during the dyeing process in the textile industry and these produce huge amounts of waste to be treated. It is projected that between 2–50% of the general dyes manufactured are obtained from industrial run-offs released or lost in wastewater, thereby causing potential damage to the marine ecosystem. Another negative effect of the dye contained in industrial run-offs is the decrease in the penetrability of radiant energy via water, thereby preventing the photochemical action of marine plants. Dyes are created to be non-biodegradable and they represent a stern problem once discharged, since they are noxious, cancer-causing and mutagenic. In trivial concentration, they are unwanted and noticeable. They are also accountable for diseases in humans^4–8^.

Extremely hazardous contaminants are organic azo-dyes are distinct by their -N = N- bond as their chromophore, which is part of an extended delocalized system comprising of the arene-groups, with acid brown 14 (AB14) and acid orange 7 (AO7-2-naphthol orange) dyes being anionic noxious aromatic azo-dyes with the chemical formula of C_26_H_16_N_4_Na_2_O_8_S_2_ and C_16_H_11_N_2_NaO_4_S. They are synthetic dyes that are utilized by various process industries responsible for the production of leather, printing (paper dyeing) and textiles. They are non-biodegradable and resist light irradiation and chemical oxidation, with adverse effects on living beings and the immediate bionetwork. A huge chunk of azo-dyes utilized in the textile industry give rise to effluents. Hence, it is critical to confiscate these hazardous pollutants from industrial waste^9–12^.

The various treatment approaches employed to confiscate these contaminants from water-soluble systems include not limited to adsorption, flocculation, membrane filtration, and chemical oxidation reaction. The adsorption procedure is thought to be more efficient due to its forthright, active, flexible operation and ensuring a benign environment, by not producing sludge for the treatment of contaminants^4,9,13^. The most applied approach for the confiscation of contaminants from wastewater is the adsorption of contaminants on activated carbon (AC)^10–12,14,15^. The application of AC for contaminants removal is owing primarily to the biosorbent material’s enormously precise surface area (SA), high sorption capacity, porosity, design simplicity and operational ease^4,16–18^.

It is an exceedingly permeable sorptive medium that has a multifaceted structure composed mainly of graphitic architecture of carbon atoms. It comprises of pores network with ducts created inside a stiff skeleton of muddled layers of carbon atoms, linked by chemical bonds, assembled haphazardly, thus creating an extraordinary porous structure of crevices and nooks between the carbon layers. They have an extraordinary level of porosity and well-developed SA with abundant oxygenated functional groups (FGs) like lactones, carbonyls, phenols, and carboxylic acids^19–23^. The pores present on the surface of AC are of substantial standing and they occur in macropores, microspores, and mesopores forms. AC can be created from a series of carbonaceous or biomass materials like wood, lignite coal, coconut shell etc. by chemically or physically activating them^24–28^.

A statistical technique called response surface methodology (RSM) is used to optimise processes when a number of independent input factors have an impact on a dependent output variable^29–33^. The answer is the name of the output variable. RSM concurrently accounts for all process variables when forecasting a result, making it an improved systematic method of experimentation^34–37^. An approximate representation of how biological neural networks process information is provided by the artificial neural network (ANN)^38^. In addition to input and output layers, most neural network layouts also include one or more hidden layers; the exact number depends on the type of experiment. The main characteristic of a neural network is its capacity to determine output values from a collection of input values through certain internal computations^38^. It may do approximations that are non-parametric or without a framework. Because ANN is strong, efficient, and very good at representing the non-linear (NL) correlations between the parameters and responses of various systems, it may be used in complicated systems^39^. By training the multiple input–output systems technique, the ANN can also analyse multivariate NL and complicated systems with sufficient data^40^.

To optimize AC adsorption performance to improve their electronic conductivity, and provide extra ion storage sites and constraint capacitance, nitrogen (N) doping is an essential effective approach. With the addition of N into the structures of carbon-based composite, the doped N_2_ atoms cause local strain inside the six-membered carbon-ring, which sequentially distorts the source carbon structure^13,41–44^. Hence, this study looks at the effectiveness of *N*-doped AC prepared from urea and zinc chloride (ZnCl_2_) treated sawdust (SD) and fish waste (FW) biomass in the adsorption of HM (Cr^6+^ ions) and dye molecules (AO7 and AB14 dyes). Also assessed in this study, are the impacts of various factors (pH, dosage and time) on the adsorption of these contaminants along with the NL of the sorption procedure using both the kinetic and isotherm models. Lastly, density functional theory calculations, ANN and RSM were applied to understand and optimize the adsorption energetics of the dye molecules (AO7 and AB14 dyes) and chromate-based compounds on the graphene as an idealized configuration for AC.

## Materials and methods

### Materials

Zinc-chloride (ZnCl_2_), potassium dichromate (K_2_Cr_2_O_7_-molecular weight (MW) = 294.19 g, an assay of 99%)), ethanol (C_2_H_5_OH), and urea (NH_2_CONH_2_), were procured from Sigma Aldrich, USA. Hydrochloric acid (HCl, M.W = 36.46 g, acid-metric assay of 30–34%) was procured from SD-Fine Chem Limited, Mumbai-India. 1,5–Diphenyl-Carbazide used as a complexing reagent for the Cr^6+^ ions assessment was procured from BDHZ-chemicals LTD Poole-England. AO7 dye (Orange II) (AO7) (C. I. 15,510) (C_16_H_11_N_2_NaO_4_S) (M.W = 350 g) and AB14 dye (C.I. 22,110) (C_26_H_16_N_4_Na_2_O_8_S_2_) (M.W = 622.547 g) were procured from ISMA dye corporation, Egypt. All the chemicals used for this research were of reasonable rating and were not purified for further usage.

### Synthesis of the N-doped AC (AC5-600)

The SD and grinded FW (60% protein) used as precursors for the synthesis of AC used in this study were obtained from a carpenter workshop and a fisherman in Alexandria, Egypt. N-doped AC was prepared from SD, grinded FW (60% protein), urea and ZnCl_2_ as precursor materials, using the hydrothermal approach at 180 °C followed by pyrolysis under N_2_ at 600 °C. A mixture of 50 g SD and 50 g grinded FW (60% protein) was treated with 10 g urea and 50 g ZnCl_2_, which were dissolved in 300 ml distilled H_2_O. This mixture was exposed to hydrothermal treatment at 180 °C for 300 min in a 500 mL Teflon cup in a stainless-steel autoclave. The product obtained from the hydrothermal treatment of the precursors treated with urea and ZnCl_2_ was dried at 125 °C overnight in an oven. Using a temperature of 600 °C, the hydrothermal product was carbonized under N_2_ for 1 h to give the primary AC. The primary AC was filtered and refluxed for 120 min in 2 N HCl. The obtained AC was filtered, cleaned with distilled H_2_O and C_2_H_5_OH and afterwards dehydrated overnight at 125 °C in an oven. The obtained AC was sonicated under 100 mL H_2_O for 0.5 h, decanted, washed with 100 ml C_2_H_5_OH and dried overnight at 125 °C in an oven. The obtained N-doped AC was labelled as AC5-600.

### Characterization of the N-doped AC (AC5-600)

The “adsorption–desorption isotherm” of the AC5-600 was assessed at N_2_ gas boiling point using a BET analyser instrument-BELSORP, Mini II, BEL Japan, Inc. To acquire mono-layer-volume (*V*_m_) (cm^3^ (STP) g^-1^), the SA (*S*_BET_) (m^2^/g), total pore volume (*V*_T_) (p/p_0_) (cm^3^/g), (C) energy-constant and mean-pore-diameter (nm), BET analysis of the isotherm was carried out. The typical pore radius was measured using Eq. (1):

1 $$r\left( {nm} \right) = \frac{{2V_{T} \left( {mL g^{{{-}1}} } \right)}}{{a_{s,BET} \left( {m^{2} g^{{{-}1}} } \right)}} \times 1000$$

The AC5-600 morphology and elemental analyses were obtained utilizing the QUANTA 250 SEM coupled with an EDX spectrometry. The AC5-600 surface FGs were measured utilizing the FTIR platinum ATR model V-100 VERTEX70 spectrometry at a wave number range of 400–4000 cm^-1^. Thermal analysis of the AC5-600 was assessed using an SDT650-Simultaneous thermal analyser instrument in the temperature range of 25–1000 °C using 5 °C per min as ramping temperature. To assess AC5-600 material’s surface chemistry, the Thermo Fisher Scientific K-Alpha X-ray photoelectron spectrometry with a pass energy of 50 eV and base pressure of approximately 10^–9^ mbar.

### Adsorption experiments

#### Adsorption study of AB14 and AO7 dyes

A standard dye stock solution of 1000 mg L^–1^ was made by dissolving 1.0 g of dye mass in a litre of distilled H_2_O. Appropriate preliminary concentrations of AB14 and AO7 dye solutions were ready by dispersing a measured stock solution of specific dye in an appropriate volume of distilled H_2_O. The batch equilibrium process was used in the sorption study of AB14 and AO7 dye to AC5-600. The influence of pH on specific dye removal was considered by agitating 100 mg of AC5-600 to 100 mL of 100 mg L^–1^ of AB14 and AO7 dye solutions with varying initial pH values of 1.5–11 using 0.1 M HCl or NaOH in shaker (JSOS-500) at 200 rpm for 2 h. The influence of adsorbent dose on specific dye elimination was considered by shaking 100 mL of 100–400 mg. L^–1^ dye concentration with varying doses of AC5-600 (50–250 mg) at the time interval of 10–120 min at pH 1.5. The equilibrium concentration (C_e_) of dye remaining in the solution after the sorption process was evaluated using a UV–visible spectrophotometer at an adsorption wavelength (λ_max_) of 461 nm (AB14 dye) and 490 nm (AO7 dye). The percentage (%) of AB14 and AO7 dye removed from a water-soluble medium was computed using Eq. (2).

2 $$Removal (\%)=\frac{\left({C}_{0}-{C}_{e}\right)}{{C}_{0}}\times 100$$

The isotherm study was achieved by contacting varying concentrations of AB14 and AO7 dye solutions (100–400 mg L^–1^) with varying AC5-600 doses (50–250 mg) at 200 rpm for 120 min at pH 1.5. In the kinetics study, 50–250 mg of AC5-600 dose was agitated with 100 mL of AB14 and AO7 dyes of initial concentration of 100–400 mg L^–1^ at 200 rpm at time intervals of 10–120 min at pH 1.5. The residual dye concentration of specific dye after the sorption process was analyzed using a UV–visible spectrophotometer. The amount of dye adsorbed (*q*_t_) at time (*t*) to AC5-600 was measured using Eq. (3).

3 $${q}_{t}=\frac{\left({C}_{0}-{C}_{t}\right)}{w}\times V$$

where *q*_t,_ *C*_0,_ *C*_e_, *C*_t_, *V* and *W* signify the sorption capacity (mg g^-1^) of the AC5-600 at time *t*; the initial concentration (mg L^–1^) of the specific dye; residual-concentration of the specific dye (mg L^–1^) and the residual-concentration of the specific dye over time *t* (mg L^–1^); the volume of dye solution (L) and the mass of AC5-600 (g).

#### Adsorption study of Cr^6+^ ions

By dispersing 2.8289 g of K_2_Cr_2_O_7_ in 1 L of distilled H_2_O, a Cr^6+^ stock solution was prepared. Preliminary concentrations of Cr^6+^ were prepared by dissolving the measured stock solution in an appropriate volume of distilled H_2_O. Batch sorption experiments were employed in this study to evaluate the removal efficiency of AC5-600 for Cr^6+^. The pH impact on Cr^6+^ sorption to AC5-600 was studied by agitating 100 mL of 100 mg L^–1^ Cr^6+^ solution with changing solution pH of 1.5–11 by means of 0.1 M HCl or NaOH with 100 mg of AC5-600. This influence of AC5-600 dose on the sorption of Cr^6+^ was studied by shaking 100 mL of initial Cr^6+^ concentration (100–400 mg L^–1^) with varying AC5-600 doses (50–250 mg) at different time intervals (10–120 min) at room temperature (25 °C) at pH 1.5. The % of Cr^6+^ ions removal by AC5-500 was calculated using Eq. (2). In the kinetics study, 50–250 mg of AC5-600 dose were added to 100 mL of Cr^6+^solution of initial concentrations of 100–400 mg L^–1^, which were shaken at 200 rpm at 25 °C at pH 1.5. Samples were set aside from the solution at different intervals time 10–120 min. Analysis of the residual-concentration of Cr^6+^ ions was carried out afterwards. The isotherm study was performed by contacting initial concentrations of Cr^6+^ solutions (100–400 mg L^–1^) with diverse weights of AC5-600 (50–250 mg) at 200 rpm, and time intervals of 10–120 min at pH 1.5. The residual-concentration of Cr^6+^ ion was determined at a UV–Visible spectrophotometer wavelength (λ_max_) of 545 nm using 1,5–diphenylcarbazide as a complexing reagent for assessing Cr^6+^ ions in solutions. Equation (2) was utilized to determine the *q*_t_ (mg g^-1^) after the sorption process.

#### AC5-600 point of zero charge (pH_PZC_)

The drift approach was utilized to assess the pH_PZC_ of AC5-600^45,46^. This involved the addition of 50 mg of AC5-600 to 0.1 M of NaCl solution. The 0.1 M of NaCl solution pH (2–10) was altered using 0.1 M HCl or NaOH. After 24 h equipoise time of the blend of AC5-600 and 0.1 M NaCl solution, the final pH was assessed. The pH_PZC_ of the AC5-600 was assessed from the plot of the change in solution pH (final pH (pH_f_)-initial pH (pH_i_) = *∆*pH) against the pH_i_ of the NaCl solution. The intersection points from the plot of *∆*pH against pH_i_ were taken as the pH_PZC_ of AC5-600.

### Error analysis of kinetic and isotherm models

The study of a huge body of data from the literature shows that in various cases, the choice has been inappropriate and an erroneous meaning has been given to the calculated coefficients. The data set examined, not physicochemical features of the sorption process, the dynamics of the process appear to impact the outcomes of researchers^47^. However, the conversion of NL Eqn. to linear-forms indirectly changes their error structure and indirectly disrupts error variance, normality assumptions and standard least squares. Accordingly, more current studies have revealed that the NL approach is better quality than the linear approach for fitting either the kinetic or isotherm models^48^.

The NL approaches utilized in this study were resolved with the Levenbergy-Marquardt iteration algorithm in Originlab Pro 2018. This regression generally comprises of the minimization or maximization of the distribution error between the predicted and experimental data, based on the convergence conditions. The error functions applied are the NL chi-square (X^2^) error, the residual sum of squares error (RSS) and root mean square error (RMSE) which are assessed using Eq. (4–5), where *q*_cal_ is the calculated (modelled), *q*_exp_ is the experimental data and *N* is the number of data points^49,50^.

4 $$X^{2} = \mathop \sum \limits_{i = 1}^{N} \left[ {\frac{{\left( {q_{exp} - q_{cal} } \right)^{2} }}{{q_{cal} }}} \right]$$

5 $$RSS=\sum_{i=1}^{N}{\left({q}_{exp}-{q}_{cal}\right)}^{2}$$

### Computational method

The van der Waals (vdW) corrected density functional theory^51–53^ as implemented in the DMOL3 code^54,55^ within the Materials Studio software suite was carried out using the Perdew–Burke–Ernzerhof (PBE) form of the generalized gradient approximation (GGA) exchange–correlation functional to model graphene and adsorbate interactions. The basis set DNP + with DFT semi-core pseudopots, hexadecapole multipolar expansion, and SCF tolerance of 10^–6^ Ha was set. The Tkatchenko–Scheffler method^56^. For semi-empirical dispersion correction was applied to account for the vdW interactions.

The graphene unit cell with 2 atoms was used to construct the 8 × 8 super cell of graphene used to study the adsorption of different dyes as well as chromate systems. The 8 × 8 supercell consists of 128 atoms of carbon and the vacuum distance of 20 Å was applied along the c-axis to prevent the interactions of the periodic images^57,58^. For the structural optimization convergence criteria of 10^−6^ eV/atom were applied to the considered configurations. The forces within these alloys were optimized to 0.03 eV/Å during geometric relaxation. The adsorption locator module was used to identify the lowest energy adsorption sites for various adsorbates(species) on the graphene surface. This module has been shown to obtain reasonable lowest energy surface-molecular adsorbate configuration^57,59^. The adsorption energy (E_Ads_) for each configuration was determined via the equation below (Eq. 6)^60,61^.

6 $${\text{E}}_{{{\text{Ads}}}} = {\text{E}}_{{{\text{SA}}}} - {\text{E}}_{{{\text{Surf}}}} {-}{\text{E}}_{{{\text{Species}}}}$$

where E_SA_, E_Surf_, E_Species_ represent the total energy of the graphene with the molecular adsorbate species, the total energy of graphene super-cell, and the total energy of molecular adsorbate species (dyes and chromate ions), respectively.

### Design of RSM experiment

The historical data design and optimal custom design of RSM, in contrast to other RSM designs like the central composite design (CCD) or Box-Behnken design (BBD), allow for the construction of mathematical models based on experimental data that has previously been collected^30^. Using the State-Ease design expert v 13.0.5.0 software, the D-optimal design to investigate the adsorption of AB14 dye, AO7 dye, and Cr^6+^ ions from adsorbent coating was examined. RSM-based CCD method was used to study the optimisation of effective parameters on the adsorption process, i.e., the effects of three independent variables (A: CA5-600 dose (mg), B: initial pollutant concentration (mg/L), and C: Contact Time (min)) on the response (R: dye or Cr^6+^ ions removal %). RSM is a statistical technique that establishes regression model equations and operating parameters using quantitative data from relevant experiments. Three processes are involved in the optimisation process: carrying out a statistically planned experiment, figuring out the mathematical model coefficients, predicting the response value, and assessing the suitability of the generated model^31,62,63^. Table S1 provides the experiment’s range and variables. Six axial points, eight factorial points, and six replicates at the centre point formed the basis of the ideal custom design for the three independent variables. Five degrees of variation were applied to the selected factors: –α, –1, 0, 1, + α. Equation (7) was used to compute the number of experiment runs.

7 $${\mathbf{N}} = 2^{K} + 2K + C = 2^{3} + 2.3 + 6 = 20$$

where *K* is the number of factors to be examined, *C* is the number of experiments carried out at the centre, and *N* is the number of runs. Table S1 displays the lowest and upper bounds for each factor. Analysis of variance (ANOVA) was used to statistically analyse the resulting model. Surface contour plots were utilised to investigate the relationships between the variables.

### ANN modeling

**Fig. 1:**
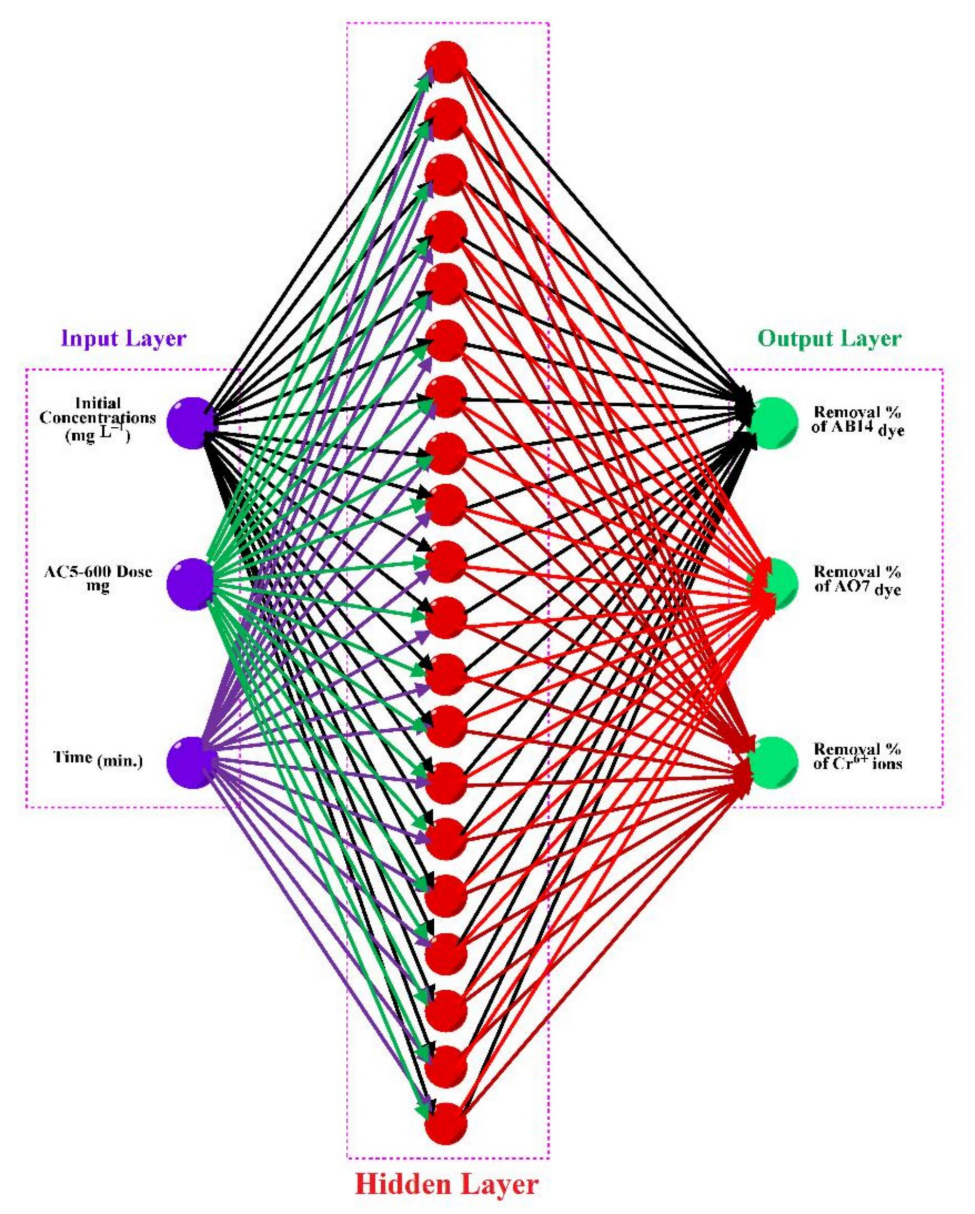
ANN architecture utilized in this study.

The ANN model constructs linear and nonlinear relationships between input and output data sets without preexisting statistical links between the input and output data by applying straightforward computational components. Neurones, which are nodes in the system network, connect the input and output data. These neurones perform a variety of tasks, including processing and storing vast quantities of data over the whole domain. Via the neurone transfer function, which produces the output signal, each neurone receives one or more input signals. After that, the output signal is passed across layers with varying intensities until the network output is reached. There are many other kinds of neural networks, but the feed-forward back-propagation NN (BPNN), sometimes called the Multi-layer Perceptron (MLP), is the most often used kind. Because it prevents the ANN algorithm’s overfitting and error correction in applications, the BPNN is very significant and well-known. An input layer (IL) (independent variable), hidden layers (HNs), an output layer (OL) (dependent variable), the activation function, the connection weight and biases, and the summation nod are the components of the BPNN model. In learning algorithms, the hidden layer’s job is to record the NL link between the input and output data. It is possible to change and manage the amount of hidden layers. Because of its versatility and flexibility, BP setup may be applied to data modelling, pattern detection, forecasting, and classification. This study uses the ANN technique to simulate the removal of various dyes, including AB14 and AO7 dyes, and HMs, like Cr^6+^ (AC5-600). MATLAB R2015b version was utilized to fit the ANN model by the Levenberg Marquart (LM) training algorithm with training (70%), validation (15%), and testing (15%). The best-fit ANN model must possess the highest R^2^ which measures the strength of the linear correlation between the acquired ED and the predicted data (PD) in the ANN and the lowest MSE which is employed as a network performance factor. The multiple layer perceptrons (MLPs) structure of the ANN model used for this study had a hidden layer (HL) of 22 neurons. This was because, after testing a large number of HL hidden neurons (between 4 and 24) during the training stage, the ANN with 22 hidden neurones demonstrated the best performance, with the highest *R*^2^ and the lowest MSE values (Fig. 1). Lastly, the independent variables were the starting concentration of dyes and HMs (mg/L), time (min), and the adsorbent dose of the (AC5-600) (mg). The dependent variables were AO7 dye, AB14 dye, and Cr^6+^ elimination^45,46^.

## Results and discussions

### Characterization

#### Analysis of the AC5-600 Morphology

**Fig. 2:**
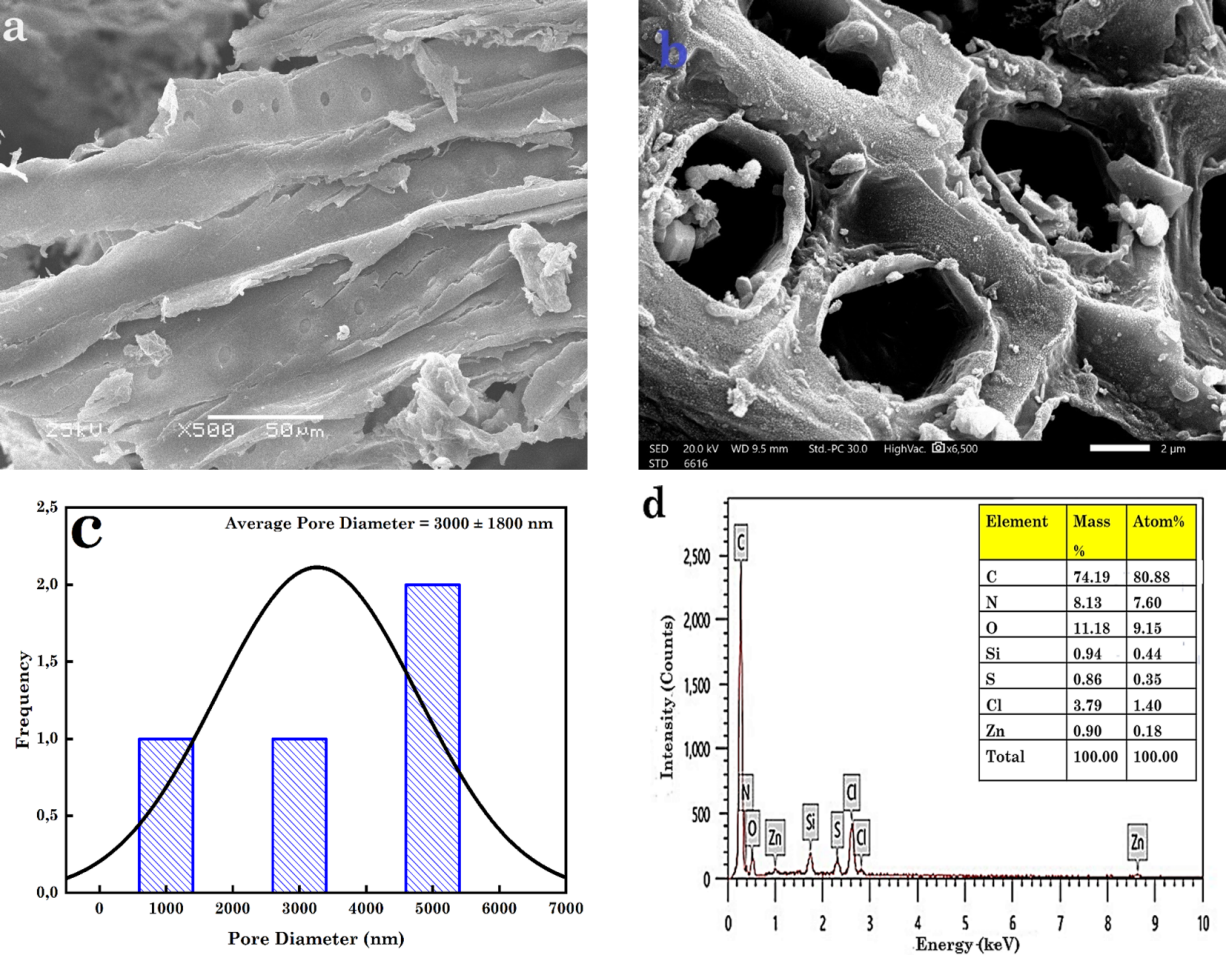
SEM image of (**a**) raw SD, (**b**) AC, (**c**) Pore size distribution determined using ImageJ software and (**d**) EDX of AC.

Figure 2 (a and b) displays the SEM images of the surface morphology of the raw SD and the prepared AC5-600 from the mixture of SD and grinded FW. Based on the SEM image in Fig. 1a, the macroscopic surface of the materials was heterogeneous, with numerous pericarps, crinkles and folds^64,65^. The SEM image in Fig. 2b shows a smooth and rough SA from the AC5-600. These smooth areas were characterized by a rough structure like a sequence of similar lines, with a few macropores noticed. These rough surface micrographs depict a diverse roughness with oval shapes. The oval structure clearly showed the presence of macropores in the AC. The pore size of the prepared AC5-600 was in the range of 1.4–4.5 μm, with an average pore size of 3.0 ± 1.8 μm based on Fig. 2c, determined using ImageJ software. The determined pore size from the ImageJ analysis shows that the AC had a macropore structure. Based on Fig. 2d, shows the EDX of the prepared AC. Based on the EDX analysis, the elemental composition of the AC showed that 74% and 11% of all elements were mostly carbon (C) and oxygen (O) (insert of Fig. 2d). Other elements present were zinc (Zn), N, silicon (Si), sulphur and chlorine (Cl).

#### Analysis of the FTIR

**Fig. 3:**
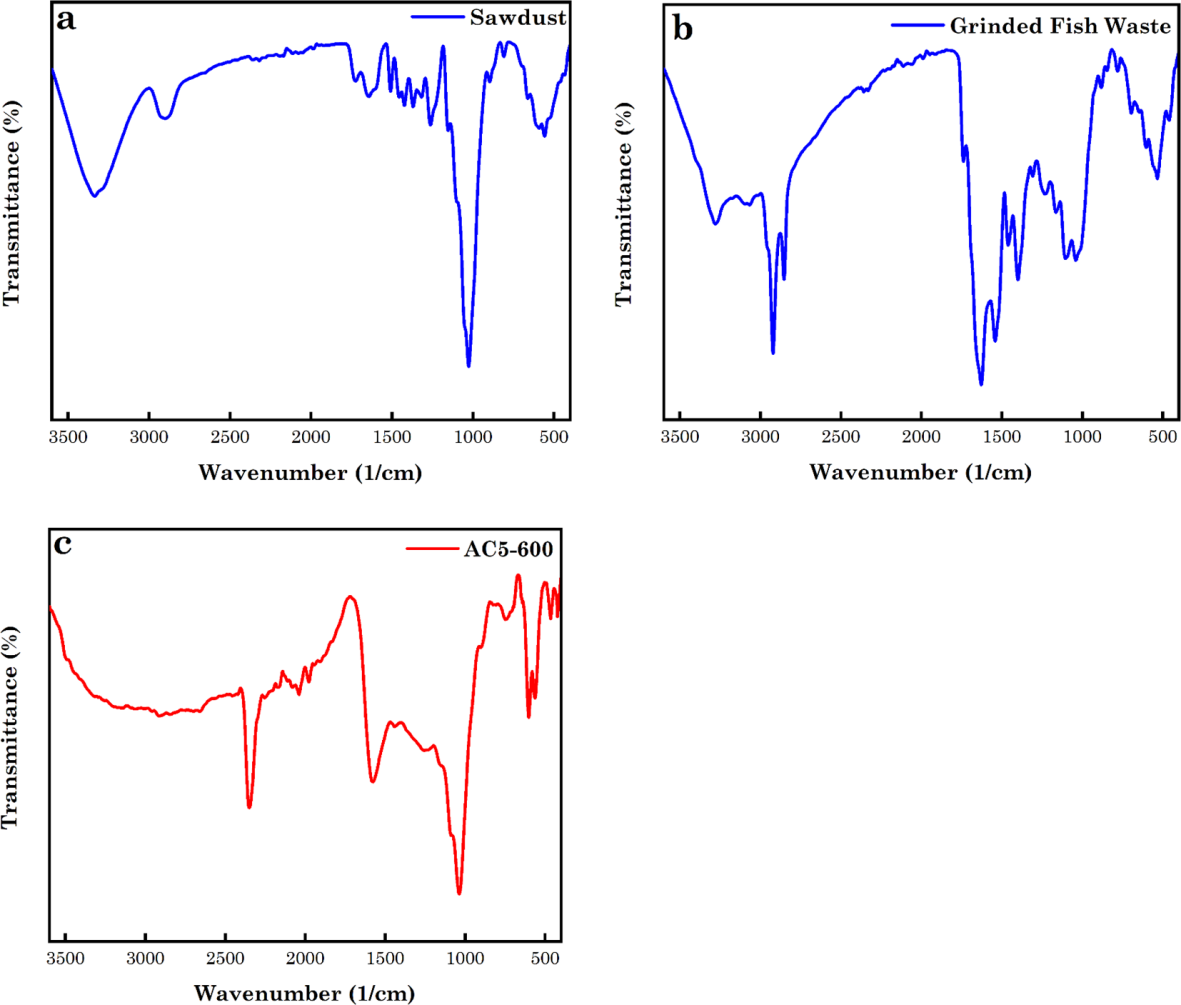
FTIR spectra of (**a**) raw SD, (**b**) grinder FW, and (**c**) AC5-600.

The FTIR spectra of the raw SD, grinded FW and the AC derived from the mixture of the raw SD and grinded FW treated with urea and ZnCl_2_ are depicted in Fig. 3(a-c). Figure 2a shows the SD IR spectra. The adsorption peaks at 3332, and 2899 cm^-1^ were attributed to the existence of intermolecular bonded -OH stretching of lignin and cellulose groups, stretching of aliphatic CH_2_ group and C-H stretching vibration from -CH_2_ groups. The peaks observed at 1729, 1640, 1508 and 1452 cm^-1^ were accredited to aldehyde C = O stretching vibration from aromatic groups of lignin, N–H amide group, C = C stretching in the aromatic compounds and -OH deformation. Peaks noticed at 1037, and 558 cm^-1^ were attributed to C-O stretching of the primary alcohol and bending vibration of aromatic compounds. The peak at 891 cm^-1^ was due to the aromatic skeletal vibration^4,66^. The IR spectra of grinded FW is shown in Fig. 3b. The broad peak at 3282 cm^-1^ was attributed to -OH groups of amide A. The peak at 2924 cm^–1^ was also ascribed to the CH_2_ stretching vibrations of the amide B or CH group (alkanes). 1735, 1628 and 1540 cm^–1^ were due to ester and lipids groups, stretching vibration of C = O of amide I, II and III and N–H bending and C-N stretching vibration. Peaks observed at 1307 and 1231 cm^–1^ were due to N–H bending and C-N stretching vibration and O = C-N group. While peaks noticed at 1162 and 1036 cm^–1^ were due to asymmetric stretching of phosphate (PO_4_^3–^) groups. The peaks at 1372 cm^–1^ showed the occurrence of nitro groups owing to NO asymmetric stretching of nitro compounds. Peaks at 1464 and 1402 cm^-1^ were due to the carbonate (CO_3_^2–^) group. The peaks at 1042, and 872 cm^–1^ were ascribed to CO groups (carboxylic acids, esters, ethers and alcohols) and NH bending in amines I and II of collagen. The peak at 841, 690, 602, and 534 cm^–1^ was due to the vibration of the phosphate (PO_4_^3–^) groups^66,67^. While Fig. 3c depicts the IR spectra of AC5-600 from the mixture of raw SD, and grinded FW treated with ZnCl_2_ and urea. The peaks at 3314 and 3156 cm^–1^ represent the -OH stretching for alcohol and N–H stretching frequencies. While the peak at 2899 cm^–1^ was ascribed to CH_2_ stretching vibrations of raw SD and amide B. The peaks at 1578 and 1446 cm^–1^ were attributed to N–H bending and C-N stretching vibration of amide II and C–H vibration in –CH_2_– deformation. The peaks at 1037 and 747 cm^–1^ were representative of C–O–C stretching and external bending of -C-H for different benzene rings substituted^66,68^. The peak at 2043 and 2345 cm^–1^ was associated with the ketamine (C = C = N) and C = N stretching vibration^69^. The FTIR analysis of raw SD-ZnCl_2_, Urea, Fish-SD-ZnCl_2_-Urea hydrothermal mixture, AC5-600-AO7 dye, and AC5-600-AB14 dye are presented in Fig. S1.

#### Analysis of the XRD

**Fig. 4:**
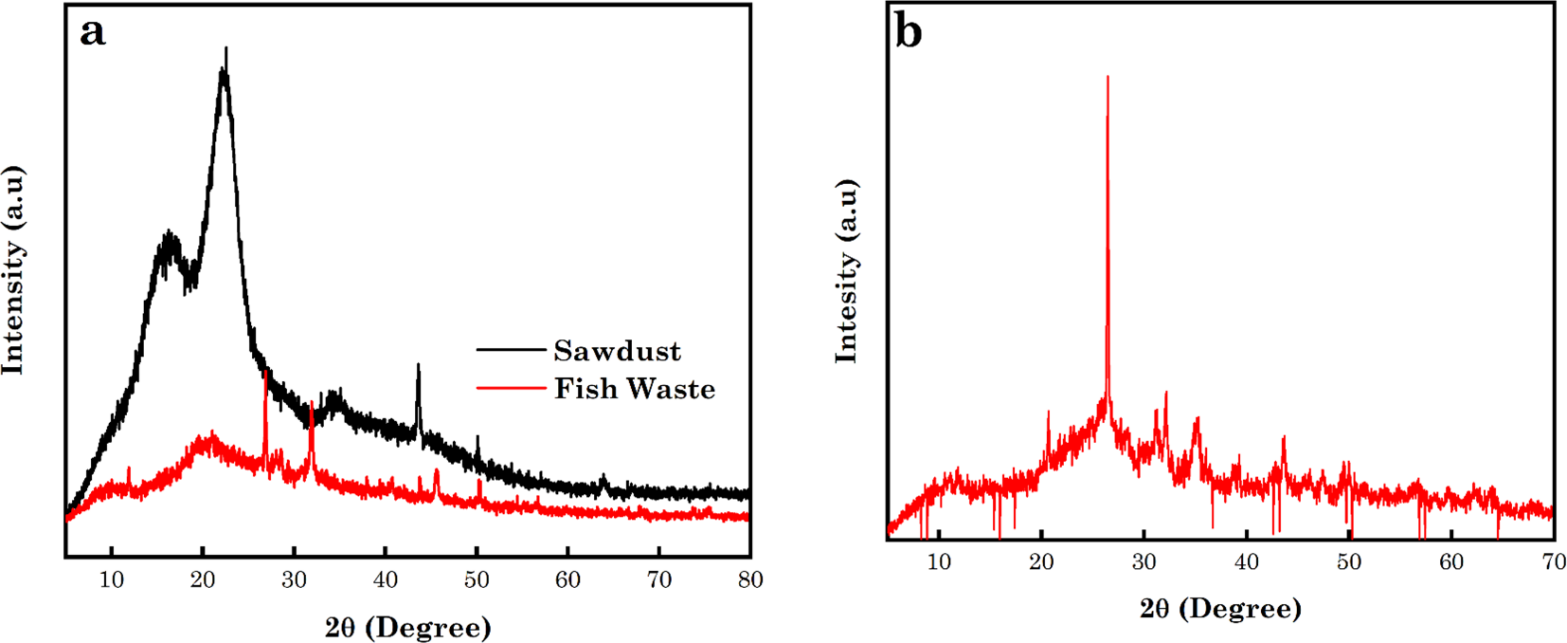
XRD pattern of (**a**) SD and FW and (**b**) synthesized N-doped AC5-600.

In lignocellulosic biomass, cellulose exists in crystalline and amorphous forms, while lignin and hemicellulose are regarded as amorphous materials. The XRD pattern of the raw SD and FW is displayed in Fig. 4a. The crystalline plane peaks of raw sawdust were found at narrow sharp 2*θ* values of 16.6 and 22° for the raw biomass, with the lower value displaying the amorphous region of the material^70–72^. As noticed with the XRD pattern of the FW, there exist crystalline and amorphous regions in the structure, which is similar to the XRD pattern of chitin and chitson. Crystalline peaks of the FW were observed at 2θ values of 9.70, 26.8, 31.9, 45.7, 50.4, 54.5 and 67.7°^73,74^. XRD spectra of AC5-600 are depicted in Fig. 4b. The extensive and weak peaks noticed at 2*θ* values of 26.5° and 43.1° were ascribed to the 002 and 100 interplanar spacings planes of carbon and planes of hexagonal graphite according to JCPDS card no. 41-1487. This showed the occurrence of a low degree of crystallinity and turbostratic carbon and a trivial graphitic crystal structure with some nm occurring in this porous carbon. The broad peak (002) depicts a feature of unstructured carbon due to the process of activation and the peak occurrence at 100 showed that the sample contained graphitic carbon^66,75–78^.

#### Analysis of the XPS

**Fig. 5:**
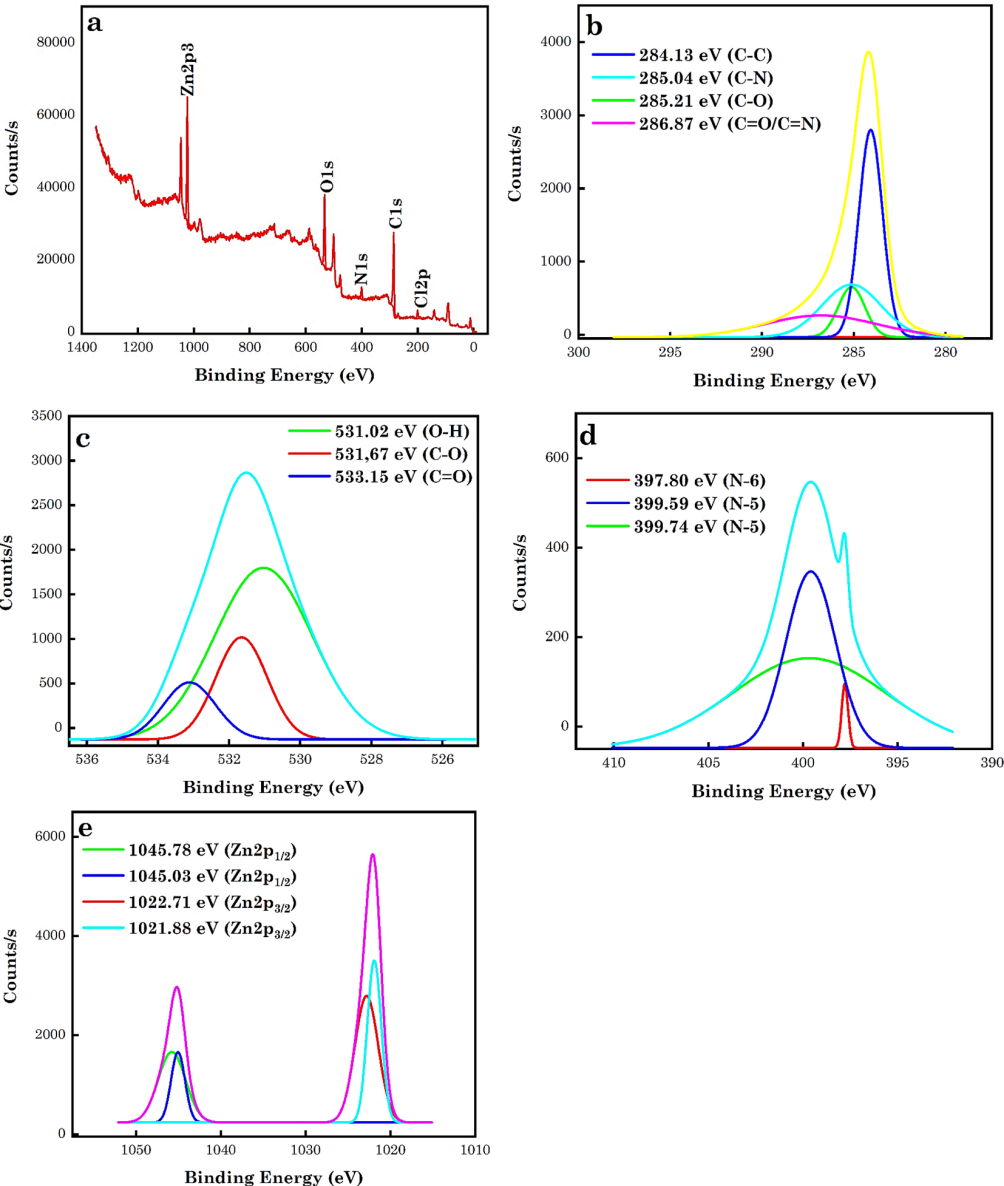
XPS spectrum of (**a**) AC5-600 Scan analysis and (**b**) C1s, (**c**) O1s (**d**) N1s and (**e**) Zn2p XPS core level spectra.

XPS was applied to quantify the FGs on the surface of the AC. Figure 5a-e shows the spectrum and spectra of AC5-600 and C1s, O1s, N1s, Cl2p and Zn2p. Based on Fig. 5a, the characteristic peaks of C1s, O1s, N1s and Zn2p were noticed on the synthesized AC5-600 and these peaks were located at 268.88, 532.18, 400.93, 201.25 and 1022.46 eV were associated with C1s, O1s, N1s, Cl2p and Zn2p. From Fig. 5b, C1s showed four peaks, obtained by the curve fitting of the C1s spectrum using Originlab software. The C1s spectrum complex signal was simplified into four peaks centred at 284.13 (41.64%), 285.04 (11.40%), 285.21 (26.38%) and 286.87 (20.59) eV, which were ascribed to sp^2^-C hybridized C–C, C-N, C-O and C = O/C = N bonds. In Fig. 5c, the O1s spectrum showed a complex signal simplified into three peaks, obtained by the curve fitting of the O1s spectrum. These three peaks were centred at 531.02 (66.93%), 531.67 (21.12%) and 533.15 eV (11.95%) and were attributed to O–H, C-O, and C = O bonds. In Fig. 5d, the N1s spectrum displayed a complex signal simplified into three peaks, obtained by the curve fitting of the N1s spectrum. These three peaks were centred at 397.80 (1.76%), 399.59 (37.94%), and 399.74 eV (60.31%) and were attributed to N-6 (pyridinic N), and N-5 (pyrrolic N) bonds. The dominant N type was pyrrolic N, followed by pyridinic N. This is linked with carbonyl or phenolic groups on the neighbouring carbon atoms. This also suggests that the N element of the AC in the form of pyrrole is involved in contaminant sorption^66,75,79–82^. In Fig. 5e, the Zn2ps spectrum displayed a complex signal simplified into four symmetry peaks, obtained by the curve fitting of the Zn2ps spectrum. These four peaks were centred at 1022.71 (36.35%), 1021.88 (27.80%), 1045.03 (12.01%) and 1045.78 eV (23.83%) and were attributed to Zn2p_3/2_ and Zn2p_1/2_ bonds, which shows the proximity of Zn2p^83^.

#### Analysis of the BET

**Fig. 6:**
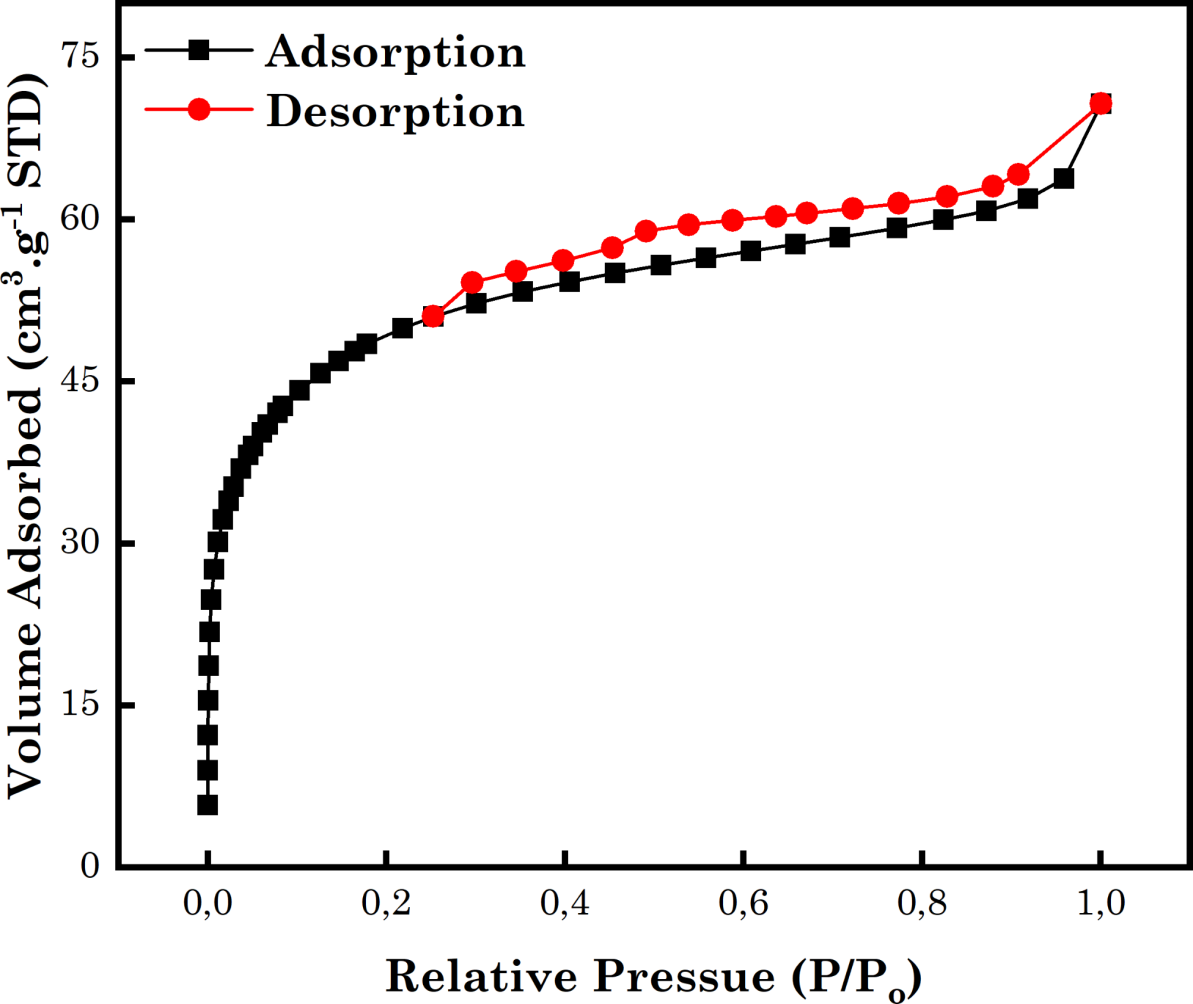
N_2_ adsorption–desorption of N-doped AC5-600 adsorbent.

The N_2_ adsorption–desorption isotherm of AC5-600 adsorbent is depicted in Fig. 6. The adsorption isotherm of the AC5-600 was a characteristic type I and IV isotherm according to the IUPAC cataloguing, owing to the sharp increase in the P/P_o_ range of around 0.0–0.2 and a highland created above 0.2. It showed a microporous and mesoporous AC material. The presence of the hysteresis loop suggested an accurate isotherm that is a blend of type 1 and IV of the IUPAC cataloguing. The preliminary part of the isotherm was type 1 with substantial uptake at low relative pressure, which matches the micropore sorption. At intermediates and high relative pressures, the isotherm was type IV with a hysteresis loop of type H4 linked with the monolayer-multilayer sorption followed by capillary condensation in a thin slit-like pore. According to the analysis of BET results, the SA, monolayer volume, mean pore diameter and total pore volume of the AC5-600 adsorbent were 554 m^2^/g, 127 cm^3^ (STP) g^–1^, 2.1 nm and 0.3 m^3^/g (Fig. S2). Hence, AC5-600 showed a mesoporosity that has an impact on the obtained isotherm profile^84,85^.

#### Analysis of the TGA

**Fig. 7:**
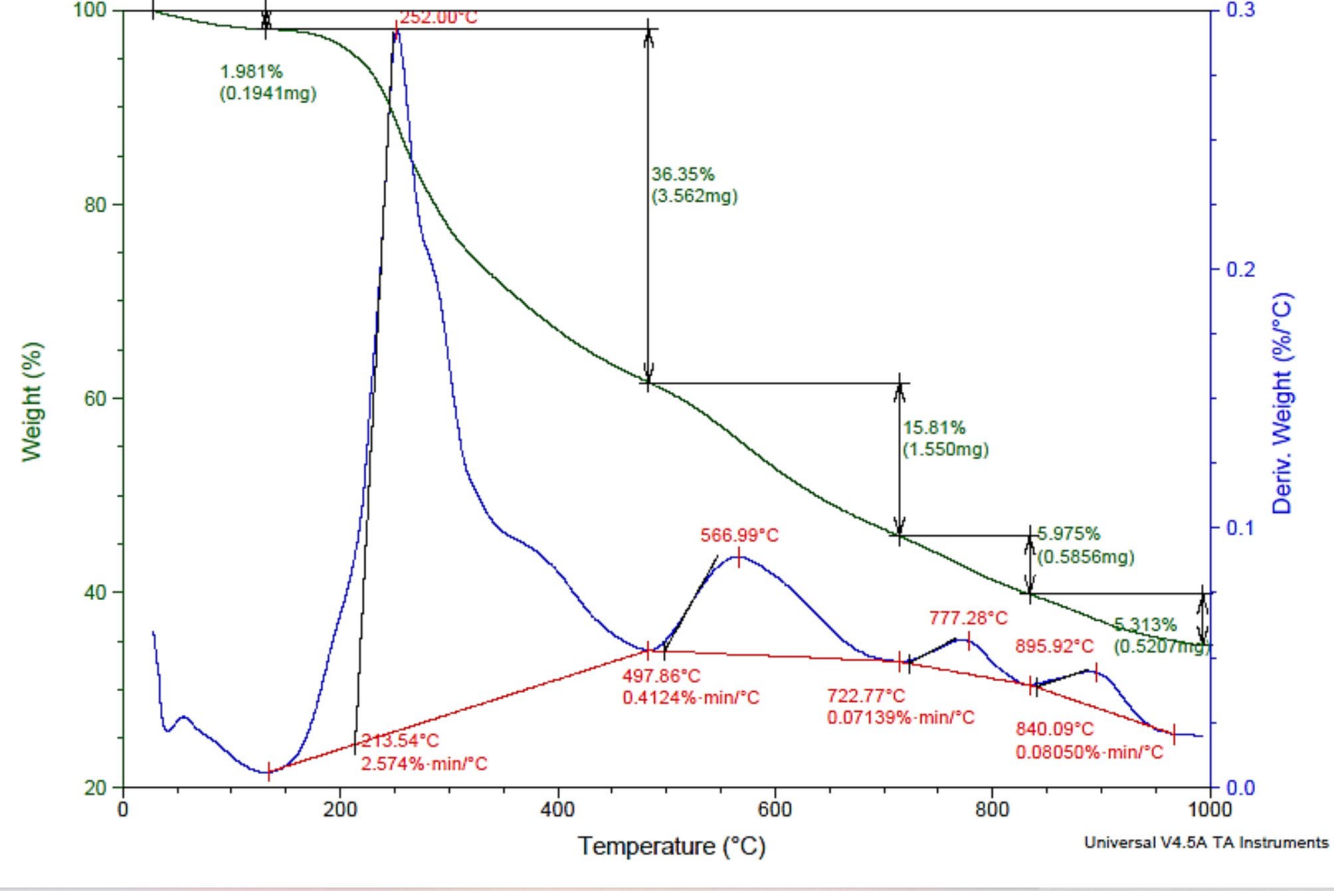
TGA and DTA studies of SD-FW-U-ZnCl_2_ prepared from SD blended with FW hydrothermal treated with urea and ZnCl_2_ followed by oven drying at 125 °C.

Figure 7 shows the TGA analysis of the prepared composite of SD-FW-U-ZnCl_2_ via the hydrothermal process. A multiphase thermochemical breakdown of SD-FW-U-ZnCl_2_ was noticed in the temperature range of 25–146, 150–480, 498–721, 723–838 and 840–990 °C with loss of weight of 1.98, 36.0, 15.8, 6.0 and 6.3%. In the opening segment of the temperature range of 25–146 °C, a miniature amount of weight loss in the SD-FW-U-ZnCl_2_ was noticed in the TGA curve, which goes together with the small DTA curve peak. This loss in weight was ascribed to the release of moisture. In the second segment, a substantial weight loss was observed in the SD-FW-U-ZnCl_2_ at the temperature range of 150–480 °C, which goes together with a huge DTA curve peak. While in the third segment, a reasonable weight loss was observed with this temperature range (498–721 °C). In the fourth and final segments, a tiny weight loss of 6.0 and 5.3% was noticed in the SD-FW-U-ZnCl_2_ at temperature ranges of 723–838 and 840–990 °C. The weight loss in the SD-FW-U-ZnCl_2_ in the fourth segment of the TGA analysis was due to ZnCl_2_ release, which was accompanied by limited volatile carbon-based compounds. This may explain why the pyrolysis at 600 °C is acceptable to produce the proposed AC5-600. The TGA-DTA analysis of raw SD material was reported in Fig. S3.

### pH effects

**Fig. 8:**
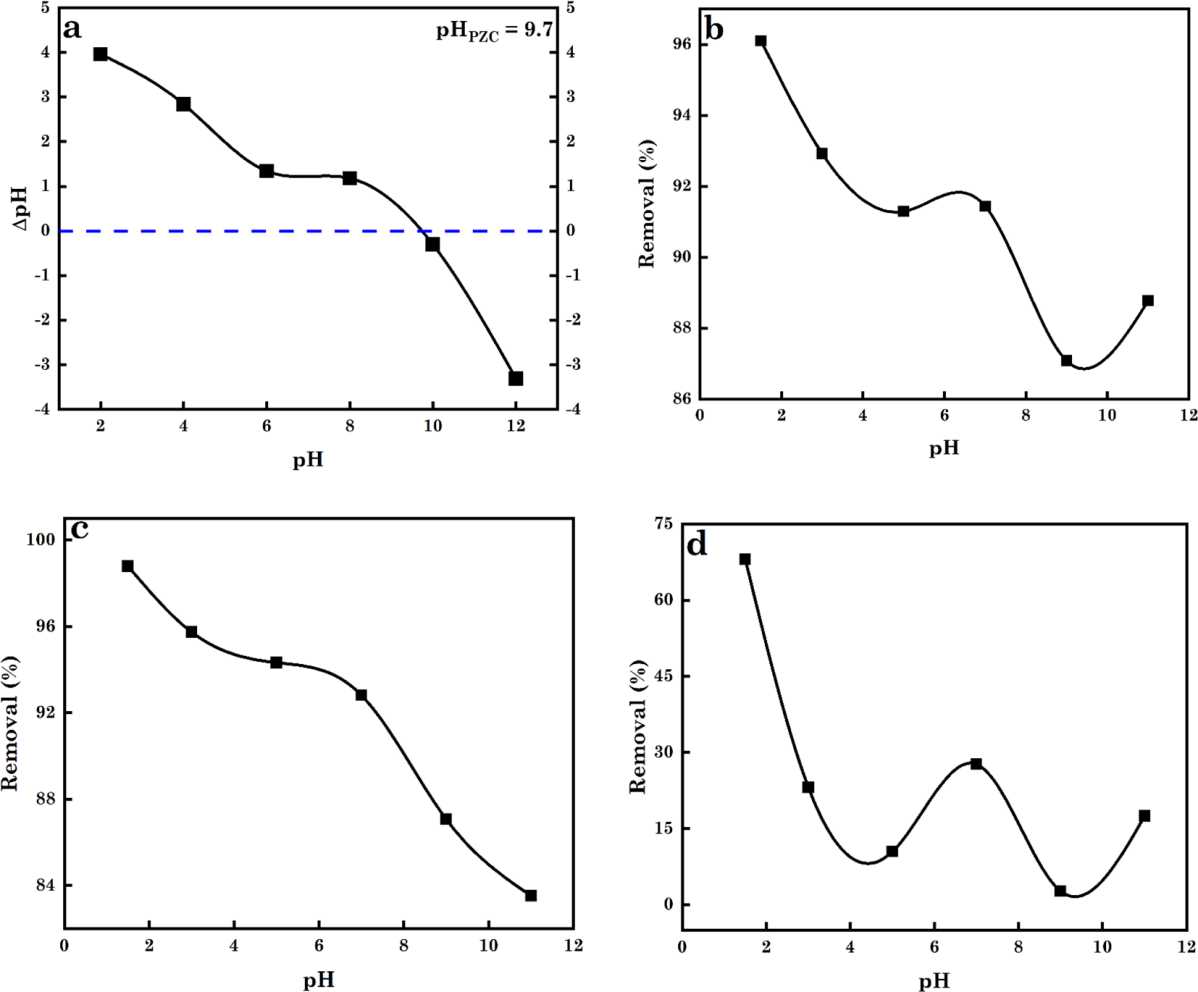
(**a**) pH_PZC_ of adsorbent, the effect of pH on the sorption of (**b**) AB14 dye, (**c**) AO7 dye and (d) Cr^6+^ ions.

A critical factor that impacts the sorption of contaminants from water-soluble systems and controls the speciation/ionization of the contaminant as well as the adsorbent surface charge is the pH^69,86^. To understand the electrostatic interactions (EI) between the adsorbent surface and the charge species of the pollutants in a water-soluble medium, the point of zero charge is measured. In a water-solution medium, the pH_PZC_ of the AC5-600 adsorbent was assessed to be 9.7. At pH < pH_PZC,_ the surface charge on the AC5-600 was positively (+ ve) charged and at pH > pH_PZC_, the surface charge on the AC5-600 adsorbent was negatively (–ve) charged (Fig. 8a)^4,87^. As observed from Fig. 8b,c,d, it was noticed that the percentage (%) of AB14 (Fig. 8b), and AO7 dye molecules (Fig. 8c) and Cr^6+^ ions (Fig. 8d) removed reduced with intensifying pH, with an ideal removal % observed at pH 1.5. For AO7 and AB14 dyes, which are anionic azo-dyes, their optimum removal % at low pH value was due to the electrostatic attraction between protonated surface sites (hydron-H^+^) on AC5-600 adsorbent (pH < pH_PZC_) and the –ve charged dye molecules. The subsequent increase in solution pH led to the increased deprotonation (hydroxyl-OH) of the sites on the AC5-600 adsorbent surface (pH > pH_PZC_) and the –ve charged dye molecules, leading to the electrostatic repulsion between the molecules of the dye and the deprotonated sites on the surface of the AC5-600. The same phenomenon was reported in the study of Eldeeb et al.^88^, El Nemr et al.^13^, Naraghi et al.^89^ and Khalil et al.^90^. As observed in Fig. 8d, the sorption of Cr^6+^ to AC5-600 was noticed to reduce with intensifying pH, with ideal removal % of Cr^6+^ observed at pH 1.5. Cr^6+^ is stated to occur in different species at various pH values (H_2_CrO_4_, HCrO_4_^−^, CrO_4_^2−^, HCr_2_O_7_^−^ and Cr_2_O_7_^2–^). The most abundant species of Cr^6+^ in an acidic solution (≤ 2) is HCrO_4_^−^ and the maximum % of Cr^6+^ removed was observed below this pH. At this pH, the increased removal was due to the EI between the protonated surface sites on the AC5-600 and the –ve-charged monovalent chromate ions (HCrO_4_^−^). With the successive intensification of the solution pH, HCrO_4_^−^ was gradually converted to other forms of Cr^6+^ (CrO_4_^2−^, and Cr_2_O_7_^2–^). At elevated pH values, there was an increased struggle for the surface sites on AC5-600 by the –ve charged OH^–^ ions in the sorption medium and CrO_4_^2−^ species. This led to a reduction in the % of Cr^6+^ removed. This could also be credited to the electrostatic repulsion between the deprotonated site on the surface of the AC5-600 adsorbent and the –ve charged CrO_4_^2−^ species at higher pH^87,91–93^.

### Effect of sorbent dosage and initial concentration

**Fig. 9:**
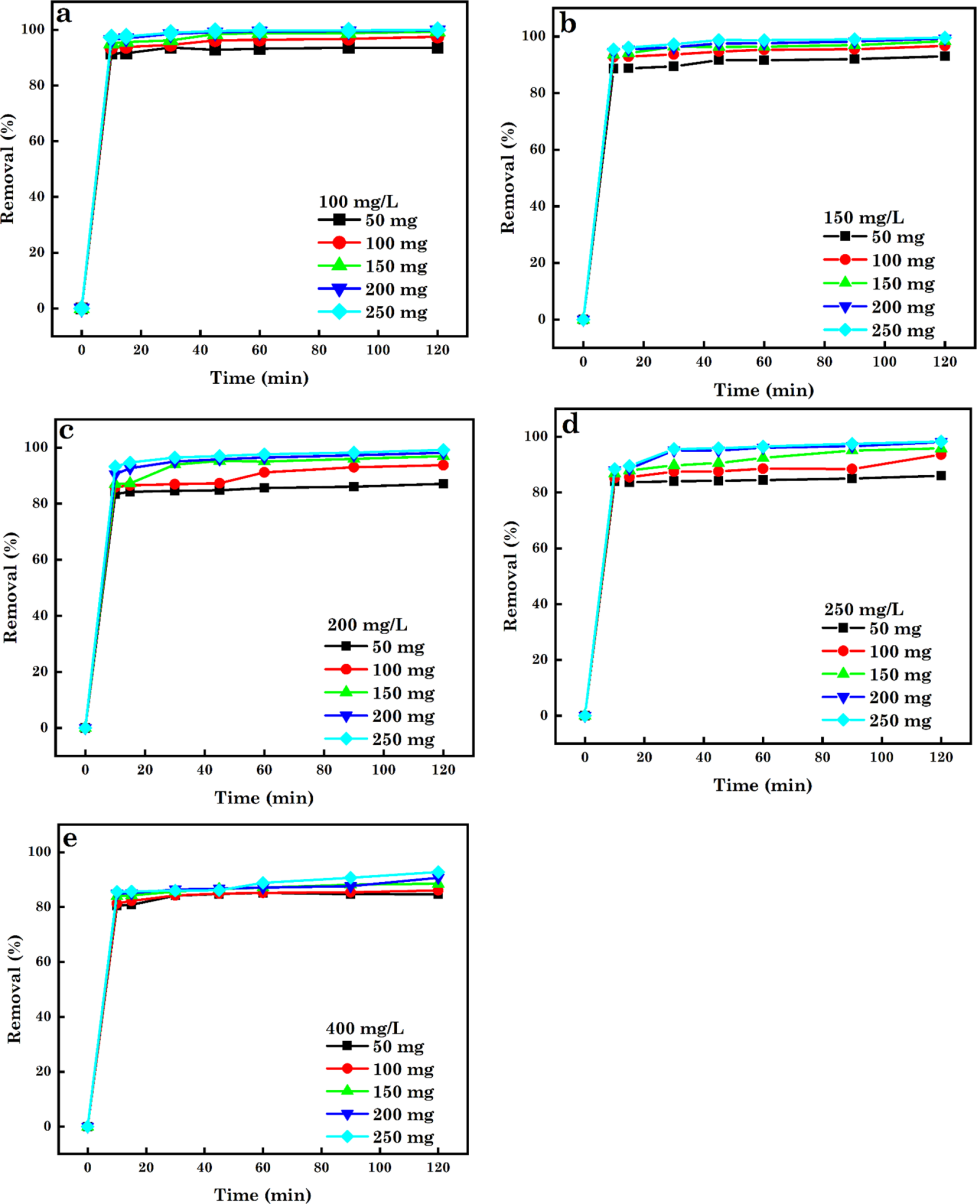
Effect of adsorbent dosage and initial-concentrations on the sorption of (**a**) 100 mg L^–1^, (**b**) 150 mg L^–1^, (**c**) 200 mg L^–1^, (**d**) 250 mg L^–1^ and (**e**) 400 mg L^–1^ of AB14 dye solution.

**Fig. 10:**
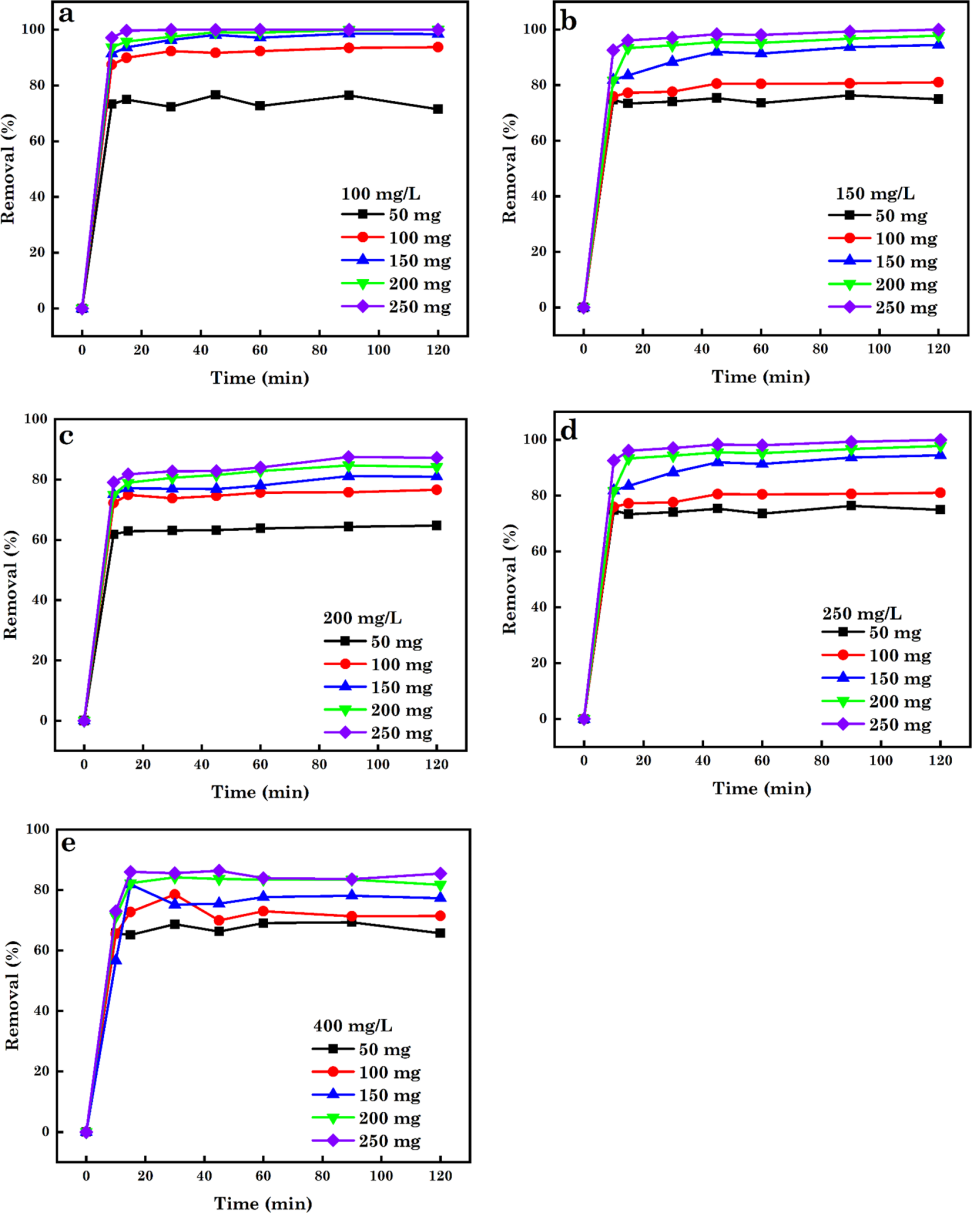
Effect of adsorbent dosage and initial-concentrations on the sorption of (**a**) 100 mg L^–1^, (**b**) 150 mg L^–1^, (**c**) 200 mg L^–1^, (**d**) 250 mg L^–1^ and (**e**) 400 mg L^–1^ of AO7 dye solution.

**Fig. 11:**
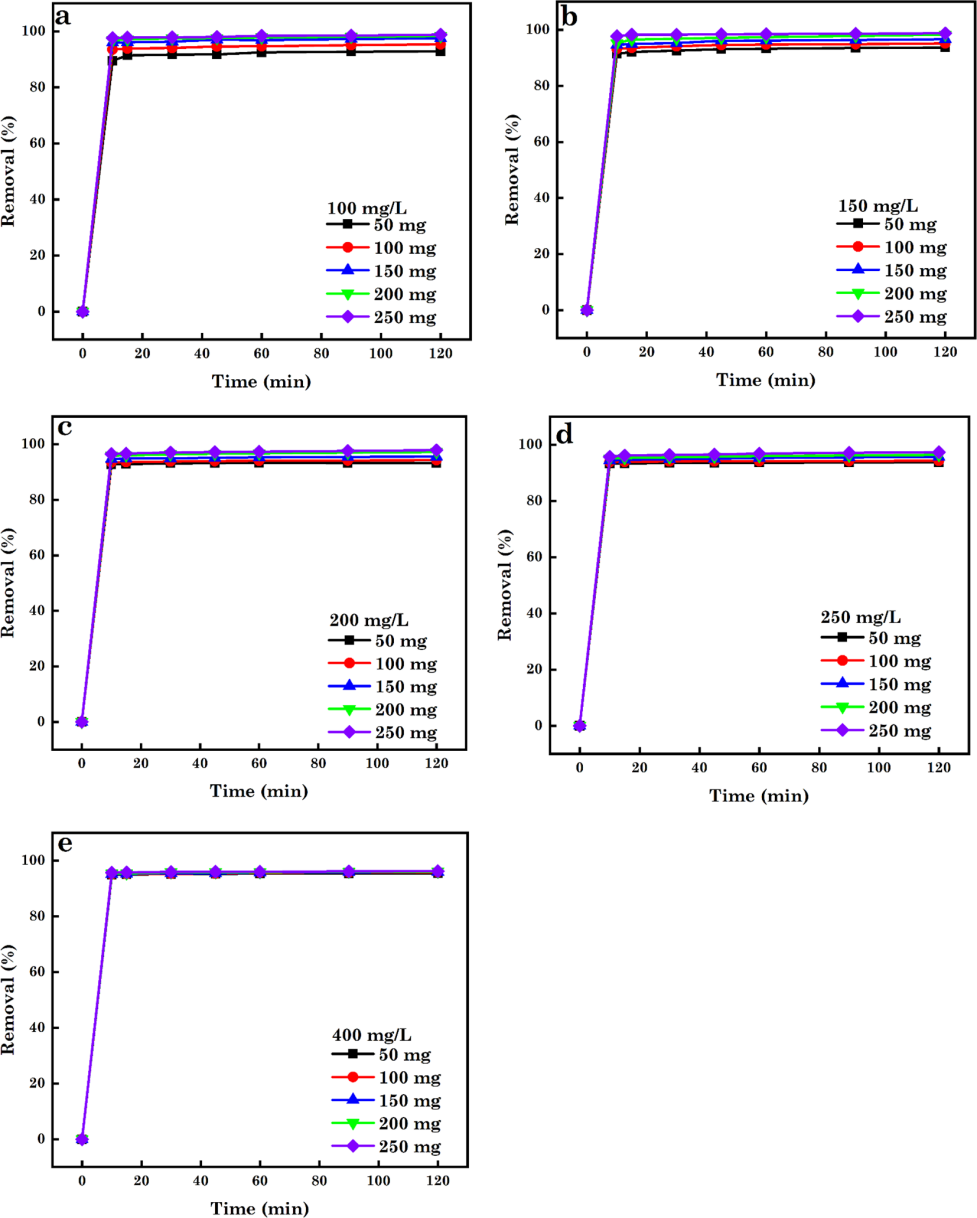
Effect of adsorbent dosage and initial-concentrations on the sorption of (**a**) 100 mg.L^–1^, (**b**) 150 mg.L^–1^, (**c**) 200 mg.L^–1^, (**d**) 250 mg.L^–1^ and (**e**) 400 mg.L^–1^ of Cr^6+^ ions.

In the sorption process, the adsorbent SA and particle size are critical factors influencing the removal % and sorption capacity (*q*_e_)^94^. As observed from Figs. 9, 10 and 11, the % of contaminants removed increased with increasing dosage of AC5-600 (50–400 mg) with increasing time (10–120 min). Further increases in the interaction time to 15 min, it was noticed that the % of various pollutants removed was constant with increasing dosage. With the use of low adsorbent dosage, a minimum quantity of pollutants was confiscated to AC5-600 due to the insufficient sorption sites accessible for the adsorbate ions and molecules to occupy. The increase in the % of contaminants removed with increasing dosage and time was attributed to the increased number of supplementary surface sorption sites available for adsorbing the various contaminants and directly corresponding to the adsorbent dosage utilized. Further increase in the dosage used to 250 mg for AB14 dye, and 400 mg for AO7 dye and Cr^6+^ ions and time of interaction resulted in a constant removal of the various contaminants, and this was attributed to the saturation of the pore volume and sorption sites. At high adsorbent dosage, the production of huge chunks of adsorbent particles led to the decreased accessible SA for the sorption of various pollutants^95–97^. The initial concentration of contaminants plays a serious part in the sorption process and offers an essential driving force needed to overcome the tasks of mass transfer between pollutants in water-soluble solutions and solid forms. The effect of initial-concentrations on confiscation efficiency was assessed in this study (Fig. 9, 10, and 11) utilizing initial concentrations of 100–400 mg.L^–1^, which was agitated with varying nano-dosages (50–250 mg) at pH 1.5 and varying time of 10–120 min. It was noticed that a surge in the initial concentration of individual pollutants induced a slight decline in the % of pollutants removed over time and varying dosage. For Fig. 10e, it was observed that the % of Cr^6+^ ions removed was constant at varying dosage and time interactions. The removal efficiency was noticed to depend on the decreased concentrations of the various contaminants, which offers a positive force and enhances the sorption process and the immense amount of the individual contaminants occasioned competition for the available binding sites on the adsorbent thereby enabling higher sorption. An increase in the concentrations of AB14, AO7 dyes and Cr^6+^ ions resulted in the measured reduction in the % of contaminants removed per unit mass of adsorbent or the extra contaminants left un-adsorbed in the solution. This was ascribed to the saturation of the available binding sites on the surface of AC5-600^91,98–101^.

### Analysis of time effects and NLM kinetic models

**Fig. 12:**
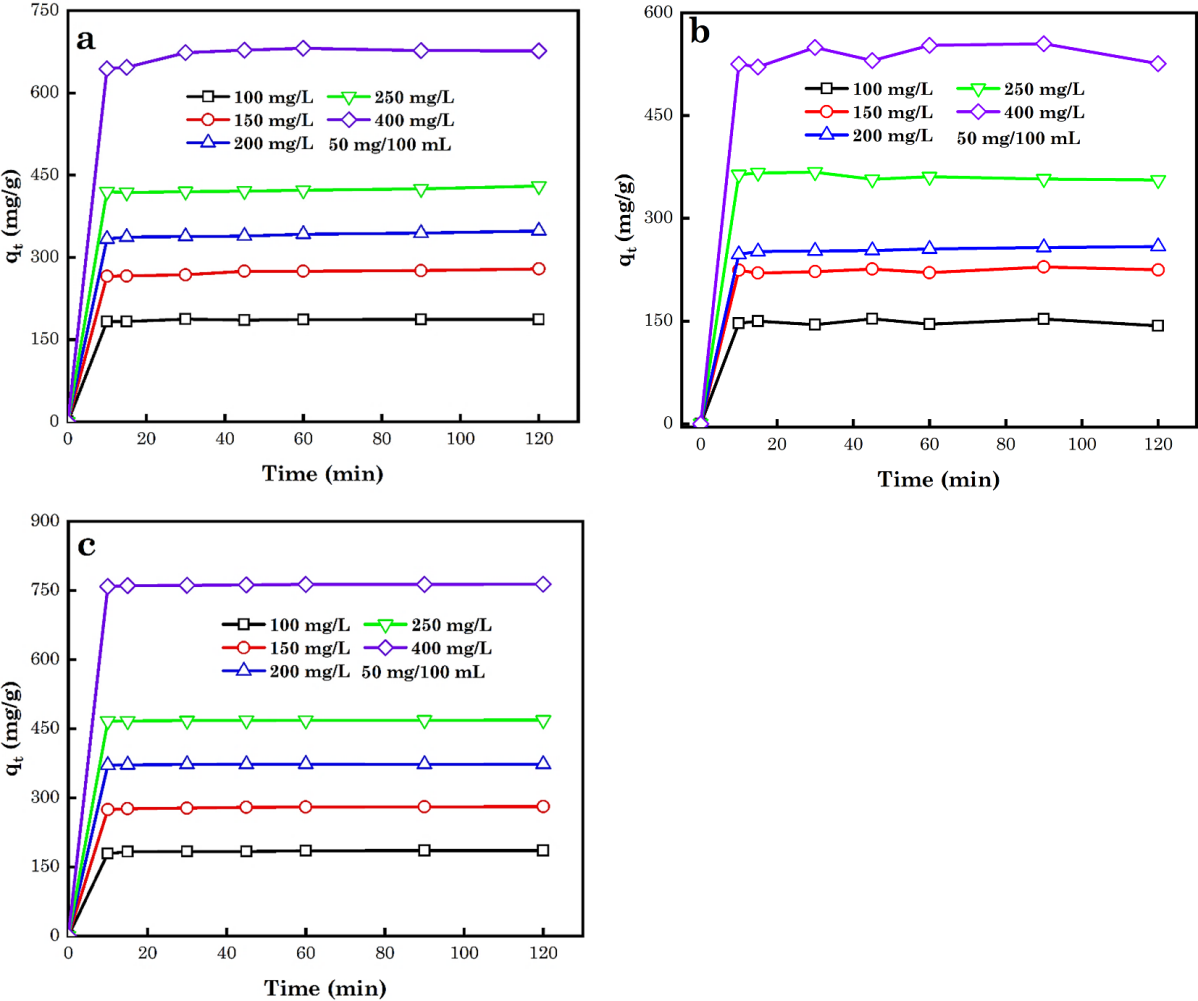
Impact of time on the sorption of (**a**) AB14 dye, (**b**) AO7 dye and (**c**) Cr^6+^ ions.

Another critical factor used in the study of the equilibrium phase and to assess the kinetic features of the sorption process is the contact time. Figure 12a-c shows the effect of time on the sorption of AB14 and AO7 dye molecules and Cr^6+^ ions. As noticed in this figure, with an increase in the time of interaction of the sorption process, the number of various contaminants sorbed by AC5-600 increased swiftly and then slowly attained equilibrium at 10 min. In the early sorption stage, a huge number of individual contaminants were adsorbed to the AC5-600 adsorbent, owing to sufficient sorption sites accessible on the surface of the adsorbent by the contaminants. Subsequently, the sorption sites accessible became saturated and the % of contaminants removed reduced and attained equilibrium over time, hence providing diffusion resistance. This could also be attributed to the vacancy of the adsorbent’s SA and their stimulating propensity towards the sorption of the adsorbate, which will drop substantially with time and SA filling^102^.

The kinetic sorption models define the sorption mechanism of the different contaminants by adsorbents and + ve assess the rate of sorption during the elimination of the contaminants from run-offs on an engineering scale and to optimize the design parameters including the adsorbate residence time and dimension of the reactor. In experimental works, the sorption process explanation using kinetic and the confiscation efficiency of contaminants by different adsorbents have been assessed often by numerous scientific models, most of which are experimental Eqn. The most common kinetic linear models, which are used to fit experimental data are the pseudo-first order model (PFOM), pseudo-second order model (PSOM), Elovich model (ELM) and the intraparticle diffusion model (IDM). The choice between these Eqn. is generally employed based on the goodness of fit, adjudicated by the coefficient of determination (*R*^2^)^102^.

**Fig. 13:**
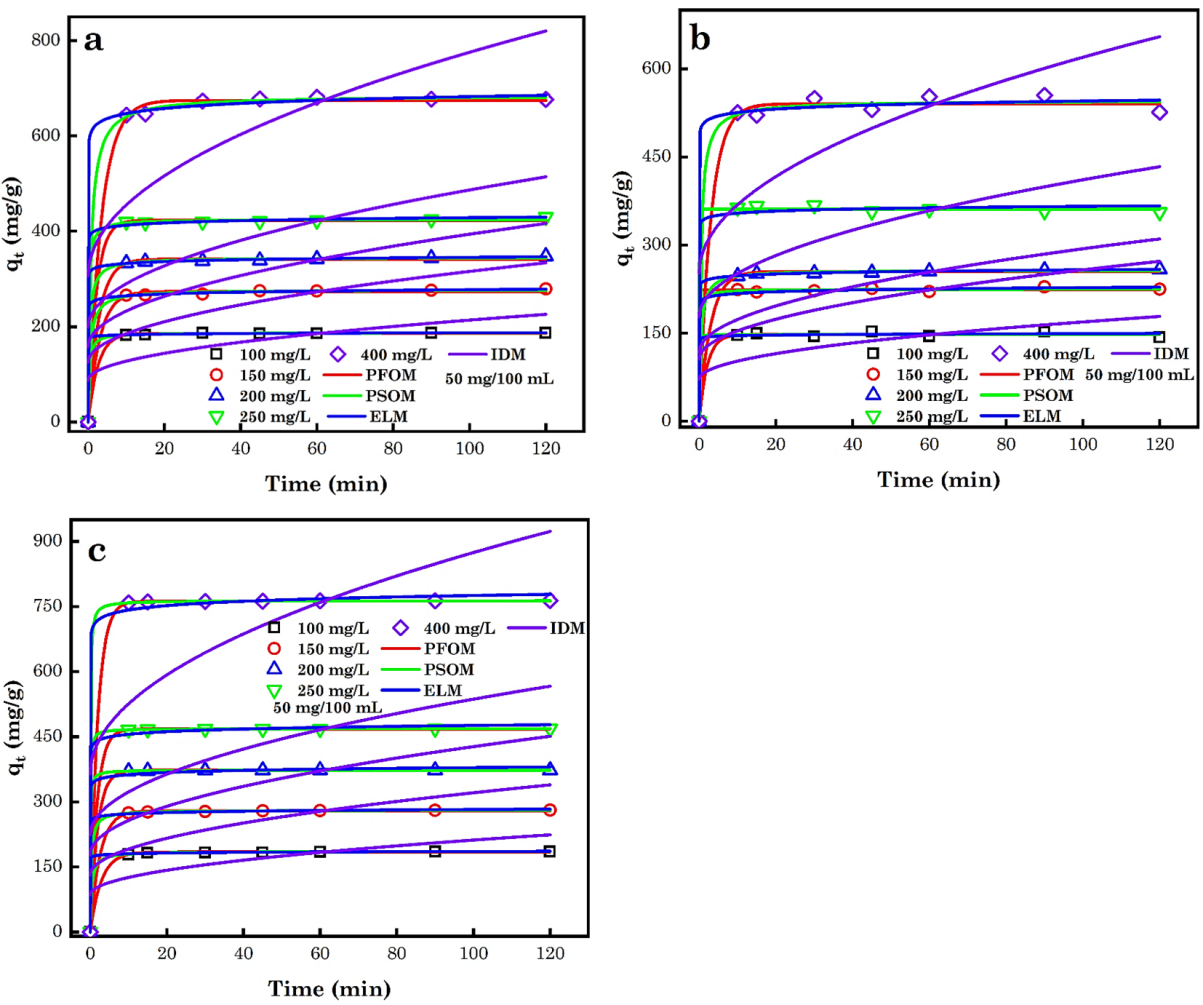
NLM of (**a**) AB14 dye, (**b**) AO7 dye and (**c**) Cr^6+^ ions employing kinetic models.

The PFOM assumes that the degree of variation in the uptakes of solute with time is directly relative to the difference in equilibrium concentration and the quantity of solute sorbed with time. The NL form of PFOM is represented by Eq. (8) ^43^. The PSOM assumes that the rate-controlling phase in the process of sorption is chemisorption. It also additionally forecasts the behaviour across the entire array of sorption. Its rate of sorption is impacted by the q_e_ rather than the adsorbate concentration. The IDM is the most generally utilized model to define the sorption mechanism, pathway of reaction and the determining step rate. The NL forms of PSOM and IDM are represented by Eq. (9–10) ^103^ and Fig. 13a-c. The ELM assist in the prediction of a system stimulation, energy, mass and surface diffusion deactivation. It has been implicitly used in effluent processing notwithstanding its preliminary use in gaseous systems. This model is based on the kinetic principle which adopts that sorption sites improve exponentially with sorption; this suggests multilayer sorption. The NL form for this model is given by Eq. (11) ^66,104^.

8 $${q}_{t}={q}_{e}\left(1-{e}^{\left(-{K}_{1}t\right)}\right)$$

9 $${q}_{t}=\frac{{q}_{e}^{2}{K}_{2}t}{\left(1+{q}_{e}{K}_{2}t\right)}$$

10 $${q}_{t}=\frac{1}{\beta }\text{ln}\left(\alpha \beta t+1\right)$$

11 $${q}_{t}={K}_{IDM}{t}^{0.5}+C$$

*K*_1_, *K*_2_ and *K*_IDMc_ are the rate constant of the PFOM (min^−1^), the rate constant of PSOM (g mg^−1^ min^−1^) and the IDM rate constant (mg g^–1^ min^1/2^). $\alpha$ (mg g^–1^ min^–1^) and $\beta$ (g mg^–1^) are constants for the ELM sorption process which symbolize the preliminary sorption rate and surface coverage constant and activation energy for chemisorption. *C* in the IDM model is the boundary diffusion influence^105,106^. The NL kinetic study results are shown in Tables 1, 2, and 3. Consequently, the obtained *q*_t_ of the PFOM and PSOM model had a substantial correlation with the experimental *q*_t_. Additionally, the PSOM, PFOM, and ELM in comparison to IDM had higher *R*^2^ and low *X*^2^, and RSS error values for the modelled experimental data of AB14 and AO7 dye molecules and Cr^6+^ ions sorption to AC5-600. The PSOM was found to best describe the sorption of AB14 and AO7 dye molecules and Cr^6+^ ions to AC5-600 adsorbent based on the low error value and high *R*^2^ values analysed. The variance between the calculated and experimental q_e_ at equilibrium was very negligible, which showed that the revealed that models were appropriate to define the kinetic sorption of AB14 dye, AO7 dye and Cr^6+^ ions to AC5-600. The IDM modelling the sorption process was not rate-controlling and diffused via the liquid film of the AC5-600, owing to the poor R^2^, X^2^ and RSS values obtained^107–109^.

**Table 1:** Determined parameters from the modelling of the experimental data from the sorption of AB14 to AC5-600 employing NL kinetic models.

Parameter			PFOM		PSOM		ELM		IDM				
AC5-600	AB14 (mg L^–1^)	*q*_*e*_ (exp.)	*q*_*e*_ (cal.)	*K*_1_	*q*_e_ (cal)	*K*_*2*_	$\alpha$	$\beta$	*K*_*IDM*_	*C*			
50 mg	100	187.1500	186.1880	0.3910	187.3628	0.0204	3.445×10^41^	0.5315	12.6283	87.55			
	150	279.0400	273.7546	0.3341	277.3460	0.0068	1.925×10^20^	0.1788	19.0860	124.99			
	200	341.8300	341.8343	0.3620	344.9905	0.0077	2.173×10^25^	0.1771	23.5705	158.09			
	250	429.9000	422.8723	0.4758	425.1420	0.0129	6.867×10^25^	0.1451	28.7979	198.86			
	400	681.2100	674.7677	0.2882	684.8823	0.0021	3.151×10^18^	0.0651	46.8888	306.71			

Parameter			PFOM		PSOM			ELM			IDM		
AC5-600	AB14 (mg L^–1^)	*R*^*2*^	*RSS*	*X*^*2*^	*R*^*2*^	*RSS*	*X*^*2*^	*R*^*2*^	*RSS*	*X*^*2*^	*R*^*2*^	*RSS*	*X*^*2*^
50 mg	100	0.9997	9.6125	1.6021	0.9999	3.9185	0.6531	0.9998	5.3717	0.8953	0.4710	15,951.76	2658.63
	150	0.9985	101.7190	16.9532	0.9994	36.1288	6.0215	0.9998	12.7652	2.1275	0.4994	32,515.49	5419.25
	200	0.9992	79.8963	16.9532	0.9996	36.1288	6.0215	0.9999	12.9135	2.1522	0.4876	52,001.14	8666.86
	250	0.9995	83.6770	13.3161	0.9997	36.4404	6.0734	0.9995	80.1978	13.3663	0.4730	82,283.11	13,713.85
	400	0.9988	489.9684	13.4962	0.9996	52.9701	8.8284	0.9992	334.1427	55.6904	0.4994	196,253.53	32,708.92

**Table 2:** Determined parameters from the modelling of the experimental data from the sorption of AO7 to AC5-600 employing NL kinetic models.

Parameter			PFOM		PSOM		ELM		IDM				
AC5-600	AO7 (mg L^–1^)	*q*_*e*_ (exp.)	*q*_*e*_ (cal.)	*K*_1_	q_e_ (cal)	*K*_*2*_	$\alpha$	$\beta$	*K*_*IDM*_	*C*			
50 mg	100	153.1370	148.0753	0.4806	147.9853	0.3733	1.521×10^44^	0.6946	9.7596	71.5655			
	150	229.0210	223.8458	1391.4964	224.9953	0.0318	2.61910^21^	0.2306	15.1953	105.8182			
	200	258.8850	255.0595	0.3420	257.5977	0.0091	1.487×10^24^	0.2280	17.6046	117.6198			
	250	367.4150	361.1864	7288.5408	361.1881	0.0002	1.039×10^30^	0.1970	23.3151	177.6198			
	400	554.7330	540.3351	0.3322	545.7125	0.0041	7.09610^26^	0.1180	36.7394	252.1502			

Parameter			PFOM		PSOM			ELM			IDM		
AC5-600	AO7 (mg L^–1^)	*R*^*2*^	*RSS*	*X*^*2*^	*R*^*2*^	*RSS*	*X*^*2*^	*R*^*2*^	*RSS*	*X*^*2*^	*R*^*2*^	*RSS*	*X*^*2*^
50 mg	100	0.9949	99.4766	16.6128	0.9948	99.8597	16.6433	0.9941	113.5579	18.9263	0.4410	10,753.5188	1792.2551
	150	0.9987	59.2533	9.8757	0.9988	52.6253	8.7709	0.9981	85.6900	14.2817	0.4684	23,339.0715	3889.8452
	200	0.9994	36.6157	6.1026	0.9997	14.5658	2.4276	0.9999	5.8048	0.9675	0.4895	28,785.8867	4797.6478
	250	0.9989	130.7186	21.7864	0.9989	130.7186	21.7864	0.9960	458.9140	76.4857	0.4236	65,866.5132	10,977.7522
	400	0.9960	1007.4196	167.9033	0.9965	894.2703	149.0451	0.9992	0.9960	1016.7792	0.4740	133,402.0745	22,233.6791

**Table 3:** Determined parameters from the modelling of the experimental data from the sorption of Cr^6+^ to AC5-600 employing NL kinetic models.

Parameter			PFOM		PSOM		ELM		IDM				
AC5-600	Cr^6+^ (mg L^–1^)	*q*_*e*_ (exp.)	*q*_*e*_ (cal.)	*K*_1_	q_e_ (cal)	*K*_*2*_	$\alpha$	$\beta$	*K*_*IDM*_	*C*			
50 mg	100	185.6380	184.4816	0.3479	185.9094	0.0151	3.366×10^33^	0.4361	12.5937	85.9552			
	150	280.9780	279.2759	0.3914	281.0075	0.0138	1.080×10^27^	0.2316	18.9712	131.1480			
	200	373.2100	372.5764	0.5278	373.1630	0.0419	1.491×10^22^	0.1417	24.8949	178.2225			
	250	468.5920	467.8708	0.5563	468.5358	0.0392	2.099×10^22^	0.1131	31.2730	223.8249			
	400	763.5190	762.3240	0.5241	763.6439	0.0190	1.999×10^22^	0.0687	51.0114	364.1989			

Parameter			PFOM		PSOM			ELM			IDM		
AC5-600	Cr^6+^ (mg L^–1^)	*R*^*2*^	*RSS*	*X*^*2*^	*R*^*2*^	*RSS*	*X*^*2*^	*R*^*2*^	*RSS*	*X*^*2*^	*R*^*2*^	*RSS*	*X*^*2*^
50 mg	100	0.9998	5.1046	0.8508	0.9999	3.0509	0.5085	0.9998	5.0726	0.8454	0.4788	15,377.8287	2562.9715
	150	0.9998	12.2290	2.0382	0.9999	1.4944	0.2491	0.9998	14.9974	2.4996	0.4725	35,765.0288	5964.1715
	200	0.9999	1.4361	0.2394	1.0000	0.4143	0.0690	0.9984	197.2987	32.8831	0.4551	66,080.0487	11,013.3411
	250	0.9999	2.0724	0.3454	1.0000	0.1780	0.0297	0.9984	311.9400	51.9900	0.4553	104,220.1916	17,370.0320
	400	0.9999	6.8870	1.1478	1.0000	0.9478	0.1580	0.9984	795.6332	132.6055	0.4565	275,939.7174	45,989.9529

### Analysis of the NL isotherm models

Intrinsic biases primarily result from linearization. To assess the fit of the isotherm Eqn. to the experimental equilibrium data, error functions are needed to enable the optimization process^107^. The Langmuir model (LNRM) assumes that monolayer sorption happens on a solid surface with corresponding similar sites. It also further proposes that no additional sorption takes place when the active sites are enclosed with contaminants. The Freudlich model (FRDM) is an experimental model signifying that the distribution of heat on the surface of the adsorbent is non-uniform specifically a heterogeneous sorption. The Temkin model (TEKM) takes into account the impact of the adsorbate and adsorbing species interaction. This model assumes that the sorption heat which is a function of temperature of the all the molecules in the layer reduces linearly rather than logarithmically with coverage owing to the interactions between the adsorbate and adsorbent. The NL model of the LNRM, FRDM and TEKM are represented by Eq. (12–14).

12 $${q}_{e}=\frac{{K}_{L}{q}_{m}{C}_{e}}{1+{K}_{L}{C}_{e}}$$

13 $${q}_{e}={K}_{F}{C}_{e}^{n}$$

14 $${q}_{e}=\frac{RT}{A}\text{In}\left({B}_{T}{C}_{e}\right)$$

*K*_L_, *q*_m_, *n*, *K*_F_, A and *B*_T_ represent the energy of sorption and capacity for a complete monolayer constant (mg g^-1^), determined sorption capacity (*q*_m_-L/mg), FRHM constants indicating sorption intensity and capacity (((mg kg^–1^)/(mg L^–1^)^n^)), TEKM constant relating to the sorption heat (kJ mol^–1^) and the empirical TEKM relating to the equilibrium binding constant associated to the optimum binding energy (L mg^–1^)^110,111^.

**Fig. 14:**
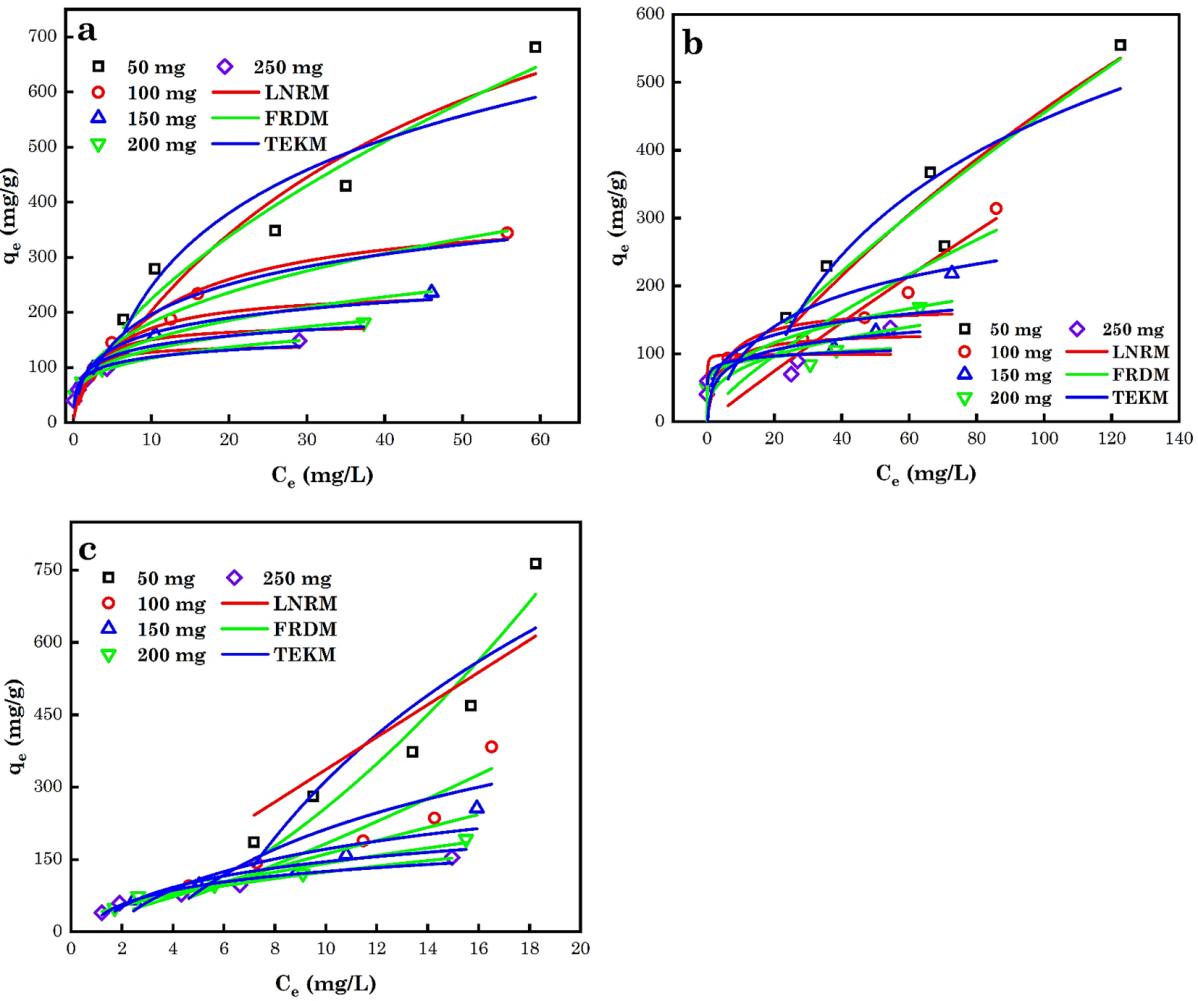
Experimental equilibrium data of the NL isotherm modelling for the sorption of (**a**) AB14 dye, (**b**) AO7 dye and (**c**) Cr^6+^ ions to AC5-600.

Owing to the intrinsic bias associated with linearization, the different parameter sets were assessed by NL regression. This provides a scientifically laborious approach to evaluating the isotherm parameters employing the unique form of the isotherm Eqns. This analysis of isotherm data is an interesting systematic method for defining sorption isotherms at constant temperatures for water and effluent remediation applications and predicting the general sorption behaviour under various working settings^112–118^. NLM of AB14 dye, AO7 dye and (c) Cr^6+^ ions sorption to AC5-600 using isotherm models is shown in Fig. 14. In this study, the sorption of AB14 dye, AO7 dye and Cr^6+^ ions sorption to AC5-600 were modelled using originlab software and the results of the determined parameters and their corresponding error values between the experimental and predicted data sets are depicted in Tables 4, 5, and 6. Based on the highest *R*^2^ values and lowest RSS and X^2^ values determined, the FRHM was found to best explain the sorption of AB14 dye, AO7 dye and Cr^6+^ ion pollutants to AC5-600 than the LNRM and TEKM from a water-soluble medium. The determined optimum *q*_m_ was 1114, 1929 and 318 mg g^-1^ for AB14 dye, AO7 dye and Cr^6+^ ions, using AC5-600 adsorbent for their removal. The obtained *K*_F_ values from the FRHM, it was observed that the values from the sorption of AB14 dye had higher *K*_F_ values than AO7 dye and Cr^6+^ ions (AB14 > AO7 > Cr^6+^) and this showed that the sorption of AB14 dye was more favourable. The *n* values for most contaminants were close to 1 and this endorses the AC5-600 surface heterogeneity. For *n* values above 1 noticed in Cr^6+^ ions sorption, it showed cooperative sorption. The comparison of the different optimal *q*_m_ for the sorption of AB14 dye, AO7 dye and Cr^6+^ ions to various AC adsorbents (Table 7), showed that the prepared AC5-600 was an efficient adsorbent for the confiscation of the different contaminants from water-soluble solutions^66,104^.

**Table 4:** Determined parameters from the modelling of the experimental data from the sorption of AB14 to AC5-600 employing NL isotherm models.

Parameter			LNRM		FRDM		TEKM			
AC5-600	AB14 (mg L^–1^)	*q*_*m (exp)*_	*q*_*m*_ (cal.)	*K*_L_	*K*_*F*_	*n*	$A$	*B*_*T*_		
50 mg	100	909.1000	1113.6890	0.0222	57.0836	0.5935	0.3585	192.9821		
	150	400.0000	395.0144	0.0963	75.8678	0.3789	1.1978	78.9953		
	200	256.4100	243.2453	0.2367	75.9556	0.2981	5.4065	40.5578		
	250	192.3080	179.7189	0.5127	74.7031	0.2486	18.1408	26.6832		
	400	153.8460	141.9997	0.9002	69.2232	0.2275	60.5339	18.5446		

Parameter			LNRM		FRDM			TEKM		
AC5-600	AB14 (mg L^–1^)	*R* ^*2*^	*RSS*	*X* ^*2*^	*R* ^*2*^	*RSS*	*X* ^*2*^	*R* ^*2*^	*RSS*	*X* ^*2*^
50 mg	100	0.8863	16,086.9038	5362.3013	0.9451	7775.1524	2591.7175	0.8609	19,681.0023	6560.3341
	150	0.9536	1653.9450	551.3150	0.9842	563.8331	187.9444	0.9731	958.8859	319.6286
	200	0.8982	1717.8143	572.6048	0.9967	55.1263	18.3754	0.9657	579.6382	193.2127
	250	0.8565	1457.4360	485.8120	0.9806	197.3325	65.7775	0.9618	387.7690	129.2563
	400	0.8007	1373.5144	457.8381	0.9981	13.0330	4.3443	0.9503	342.6897	114.2290

**Table 5:** Determined parameters from the modelling of the experimental data from the sorption of AO7 to AC5-600 employing NL isotherm models.

Parameter			LNRM		FRDM		TEKM			
AC5-600	AO7 (mg L^–1^)	*q*_*m*_ (exp.)	*q*_*m*_ (cal.)	*K*_L_	*K*_*F*_	*n*	*A*	*B*_*T*_		
50 mg	100	1250.0000	1928.5622	0.0031	12.0626	0.7884	0.0765	219.1844		
	150	384.6150	3430.0300	0.0011	10.7335	0.7344	0.4101	66.5563		
	200	204.0820	170.1932	0.1926	43.4057	0.3285	4.5341	28.3598		
	250	153.8460	132.5135	0.2849	36.2617	0.3293	4.3727	23.5993		
	400	128.2050	99.4250	15.0433	67.1197	0.1182	11,916.7037	7.8126		

Parameter			LNRM		FRDM			TEKM		
AC5-600	AO7 (mg L^–1^)	*R* ^*2*^	*RSS*	*X* ^*2*^	*R* ^*2*^	*RSS*	*X* ^*2*^	*R* ^*2*^	*RSS*	*X* ^*2*^
50 mg	100	0.8808	11,551.7744	3850.5915	0.8942	10,258.5853	3419.5284	0.8224	17,216.1161	5738.7054
	150	0.7897	6211.8484	2070.6161	0.8232	5222.3284	1740.7762	0.6249	11,080.6854	3693.5618
	200	0.5080	6633.1295	2211.0432	0.7190	3788.2161	1262.7387	0.6312	4972.4262	1657.4754
	250	0.2802	5829.7052	1943.2351	0.4404	4531.8698	1510.6233	0.3749	5062.8832	1687.6278
	400	0.2712	4062.1022	1354.0341	0.3718	3501.0893	1167.0298	0.3454	3659.6549	1219.8850

**Table 6:** Determined parameters from the modelling of the experimental data from the sorption of Cr^6+^ to AC5-600 employing NL isotherm models.

Parameter			LNRM		FRDM		TEKM			
AC5-600	Cr^6+^ (mg L^–1^)	*q*_*m*_ (exp.)	*q*_*m*_ (cal.)	*K*_L_	*K*_*F*_	*n*	$A$	*B*_*T*_		
50 mg	100	-1000	1.2823×10^7^	2.6192×10^–6^	5.5325	0.5999	0.1808	528.1498		
	150	10,000	521,486.3620	3.7507×10^–5^	10.780	0.8131	0.3135	186.0083		
	200	555,556	2986.3589	0.0057	21.5481	1.1439	0.6539	91.2197		
	250	277,778	318.4825	0.0852	35.0758	1.6496	1.2131	58.2071		
	400	208,333	213.4666	0.1521	37.8308	1.9402	1.8572	42.8497		

Parameter			LNRM		FRDM			TEKM		
AC5-600	Cr^6+^ (mg L^–1^)	*R* ^*2*^	*RSS*	*X* ^*2*^	*R* ^*2*^	*RSS*	*X* ^*2*^	*R* ^*2*^	*RSS*	*X* ^*2*^
50 mg	100	0.8135	36,677.1987	12,225.7329	0.9218	15,376.1237	5125.3746	0.8184	35,717.7596	11,905.9199
	150	0.8598	6852.3009	2284.1003	0.8779	5970.7761	1990.2587	0.7721	11,142.7486	3714.2495
	200	0.9413	1263.2119	421.0706	0.9502	1071.6619	357.2206	0.8209	3850.5291	1283.5097
	250	0.9321	814.8751	271.6250	0.9704	355.1369	118.3790	0.9057	1131.4607	377.1536
	400	0.9564	335.4439	111.8147	0.9918	62.8052	20.9351	0.9471	406.7493	135.5831

**Table 7:** Comparison of the determined sorption capacities (*q*_m_) of various AC materials for the sorption of AB14 dye, AO7 dye and Cr^6+^

Adsorbent/Adsorbate	q_m_ (mg.g^-1^)	References
AC (NDAC) from saw dust/ZnCl_2_/AB14 dye	909.09	^13^
Microporous AC from Pisum sativum Pods/AO7 dye	473.93	^44^
Mandarin-CO-TETA derived from mandarin peels/AB14 dye	416.67	^88^
Orange peel magnetic AC /AO7 dye	357.14	^90^
AC obtained from a waste lignocellulosic material/Cr^6+^	196.38	^111^
Spent coffee grounds/AO7 dye	119.5	^113^
De-inked Pulp waste Sludge AC (DIPSAC)/AO7 dye	12.88	^114^
AC produced from Tectona grandis tree SD /Cr^6+^	127	^115^
Fe_3_O_4_-loaded granular AC/Cr^6+^	10.44	^118^
AC5-600/AB14 dye	1114	This study
AC5-600/AO7 dye	1929	This study
AC5-600/Cr^6+^ ions	318	This study

### Computational adsorption of the species on graphene nanomaterial

The optimized unit cell for the graphene with primitive hexagonal Bravais lattice and group name P6/MMM has a lattice constant of 2.468 AA in the a- and b-axis with an interlayer spacing of 20 AA in the c-axis to prevent periodic image interactions. The constructed 8 × 8 supercell has a lattice constant of 19. 744 AA. The choice of the super-cell was necessitated to alloy for the adsorption of the considered dyes^117,118^.

**Fig. 15:**
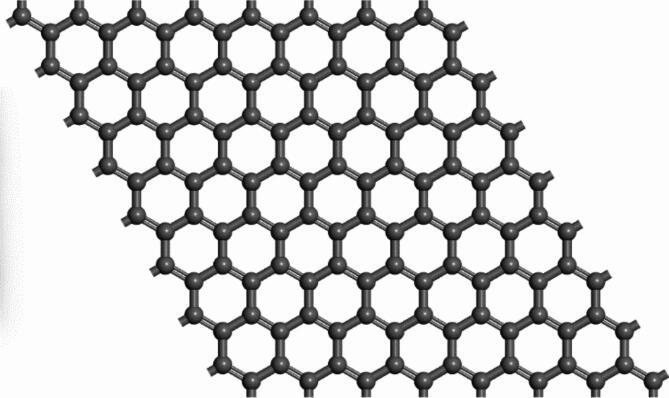
Ball and stick model of pristine graphene supercell sheet with carbon atoms in grey.

**Fig. 16:**
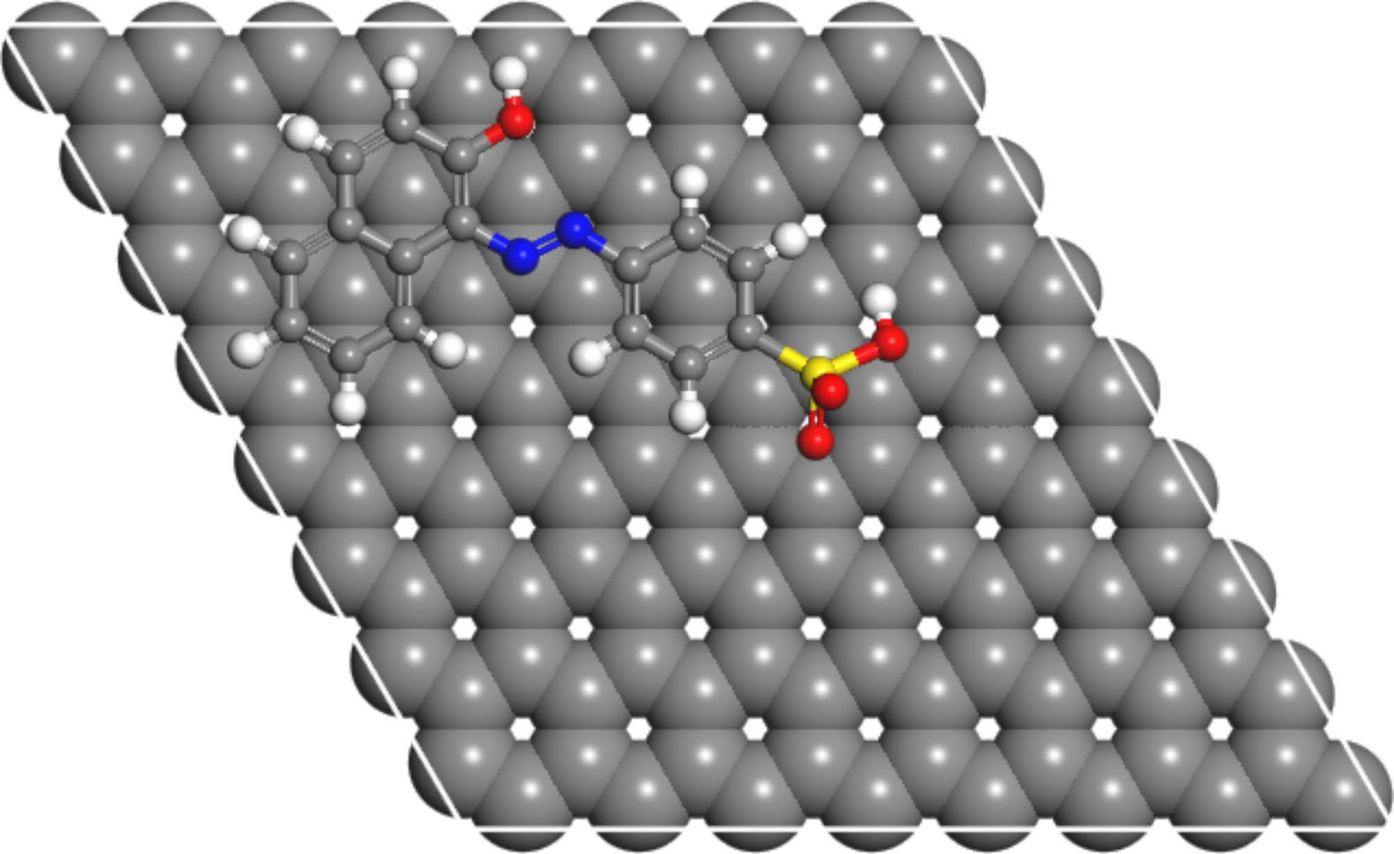
Schematic of graphene supercell with the adsorbed AB14 dye compound, where the grey, white, yellow, blue and red balls are carbon, hydrogen, sulphur, sodium and oxygen atoms, respectively.

In the current study, the ability of the dyes and chromate species to stably adsorb on pristine graphene compound is considered and thus effectively acting as an agent for the removal of these substances is determined. The adsorption locator module in BIOVIA Materials Studio was used to determine the optimal adsorption configurations for these species as described in “Computational method” section. Figures 15 and 16 show the pristine structure of graphene as well as graphene with AO7 dye.

The calculated sorption energies for the AO7 and AB14 dyes are -4.492 and -8.090 eV, respectively, which is indicative of strong exothermic characteristics. These strong binding energies indicate that pristine graphene is capable of abstracting these species. Considering, that the observed binding energies lend credence to the experimentally observed behaviour in the current study, the synthesized AC is capable of acting as a scrubber for these dyes. Considering the adsorption energies of Cr-based ions on the graphene surface, different configurations of Cr configurations were considered. The Cr_2_, CrO_3_, CrO_4_, and CrO_4_H species adsorbed on the graphene structure with adsorption energies of 2.563, 1.789, 1.226 and 1.928 eV, respectively. The implication is that the ideal surface of graphene is endothermic for the abstraction of these species and not suitable for the removal of these species. However, understanding that the experimental considered AC configurations is not pristine graphene. Further investigation would be carried out in subsequent studies on different AC and the functionalization of graphene for the abstraction of chromate species.

### RSM study

The interaction effects of three crucial variables, namely contact duration, adsorbent dosage, and beginning dye concentration, on the percentage of AB14 dye, AO7 dye, and Cr^6+^ ions removed were examined using the design matrix. Table S2 displays the responses and the experimental design. The following polynomial Eqs. (15–16) for AB14 dye removal percentage were created based on the findings obtained:

15 $$\begin{aligned} {\text{Removal}}\;\% \;{\text{for}}\;{\text{Coded}}\;{\text{Factors}} = & 92.66 + 4.14{\text{A}} - 5.39{\text{B}} + 2.28{\text{C}} + 0.2438{\text{AB}} + 0.0342{\text{AC}} \\ & + 1.36{\text{BC}} - 2.58{\text{A}}^{{2}} + 0.2931{\text{B}}^{2} + 0.4491{\text{C}}^{2} \\ \end{aligned}$$

16 $$\begin{aligned} {\text{Removal}}\;\% \;{\text{for}}\;{\text{Actual}}\;{\text{Factors}} = & 91.84335 + 0.114376\;{\text{Adsorbent}}\;{\text{dosage}} \\ & - 0.056523\;{\text{Dye}}\;{\text{Conc}}. - 0.022752{\text{Time}}. + 0.000016{\text{Adsorbent}}\;{\text{dosage}} \times {\text{Dye}}\;{\text{Conc}}. \\ & + 6.51812{\text{E}} - 06\;{\text{Adsorbent}}\;{\text{dosage}} \times {\text{Time}} \\ & + 0.000172\;{\text{Dye}}\;{\text{Conc}}. \times {\text{Time}} - 0.000258\;{\text{Adsorbent}}\;{\text{dosage}}^{2} \\ & + 0.000013{\text{ Dye}}\;{\text{Conc}}.^{2} + 0.000163\;{\text{Time}}^{2} \\ \end{aligned}$$

The following polynomial Eqs. (17–18) for AO7 dye removal percentage were created based on the findings obtained:

17 $$\begin{aligned} {\text{Removal}}\;\% {\text{for}}\;{\text{Coded}}\;{\text{Factors}} = & 83.52 + 9.{\text{58A}} - 6.{\text{13B}} + 0.{\text{6575C}} - 1.{\text{92AB}} \\ & + 1.0{\text{7AC}} - 0.600{\text{7BC}} - 6.{\text{22A}}^{2} + 6.{\text{83B}}^{2} - 4.{\text{43C}}^{2} \\ \end{aligned}$$

18 $$\begin{aligned} {\text{Removal}}\;\% \;{\text{for}}\;{\text{Actual}}\;{\text{Factors}} = & 72.17516 + 0.300502\;{\text{Adsorbent}}\;{\text{dosage}} \\ & - 0.168403\;{\text{Dye}}\;{\text{Conc}}.\;0.217828\;{\text{Time}}. \\ & - 0.000128{\text{Adsorbent}}\;{\text{dosage}} \times {\text{Dye}}\;{\text{Conc}}. \\ & + 0.000205{\text{Adsorbent}}\;{\text{dosage}} \times {\text{Time}} \\ & - 0.0000{\text{76 Dye Conc}}. \times {\text{Time}} - 0.000622\;{\text{Adsorbent}}\;{\text{dosage}}^{2} \\ & + 0.00030{\text{4Dye}}\;{\text{Conc}}.^{2} - 0.001607\;{\text{Time}}^{2} \\ \end{aligned}$$

The following polynomial Eqs. (19–20) for the elimination percentage of Cr^6+^ ions were created based on the findings obtained:

19 $${\text{Removal }}\% {\text{ for Coded Factors }} = {95}.{18} + {2}.{\text{42A}}{-}0.{\text{4773B}} + {1}.{\text{21C}}{-}0.{\text{6689AB}}{-}0.{\text{4378AC}} + 0.{7}0{\text{46BC}}$$

20 $$\begin{aligned} {\text{Removal}}\;\% \;{\text{for}}\;{\text{Actual}}\;{\text{Factors}} = & {89}.{79433} + 0.0{4}0{\text{943Adsorbent}}\;{\text{dosage}} - 0.00{\text{2533Dye}}\;{\text{Conc}}. \\ & + 0.0{131}0{\text{2Time}} - 0.0000{\text{45Adsorbent}}\;{\text{dosage}} \times {\text{Dye}}\;{\text{Conc}}. \\ &- 0.0000{\text{83Adsorbent}}\;{\text{dosage}} \times {\text{Time}} \\ & + 0.0000{\text{89Dye}}\;{\text{Conc}}. \times {\text{Time}} \\ \end{aligned}$$

Predictions on the response for specific amounts of each element may be made using the equation expressed in terms of the actual factors. In this case, each factor’s levels have to be stated in their original units. Because the intercept is not in the centre of the design space and the coefficients are scaled to account for the units of each element, this equation should not be used to calculate the relative influence of each factor.

**Fig. 17:**
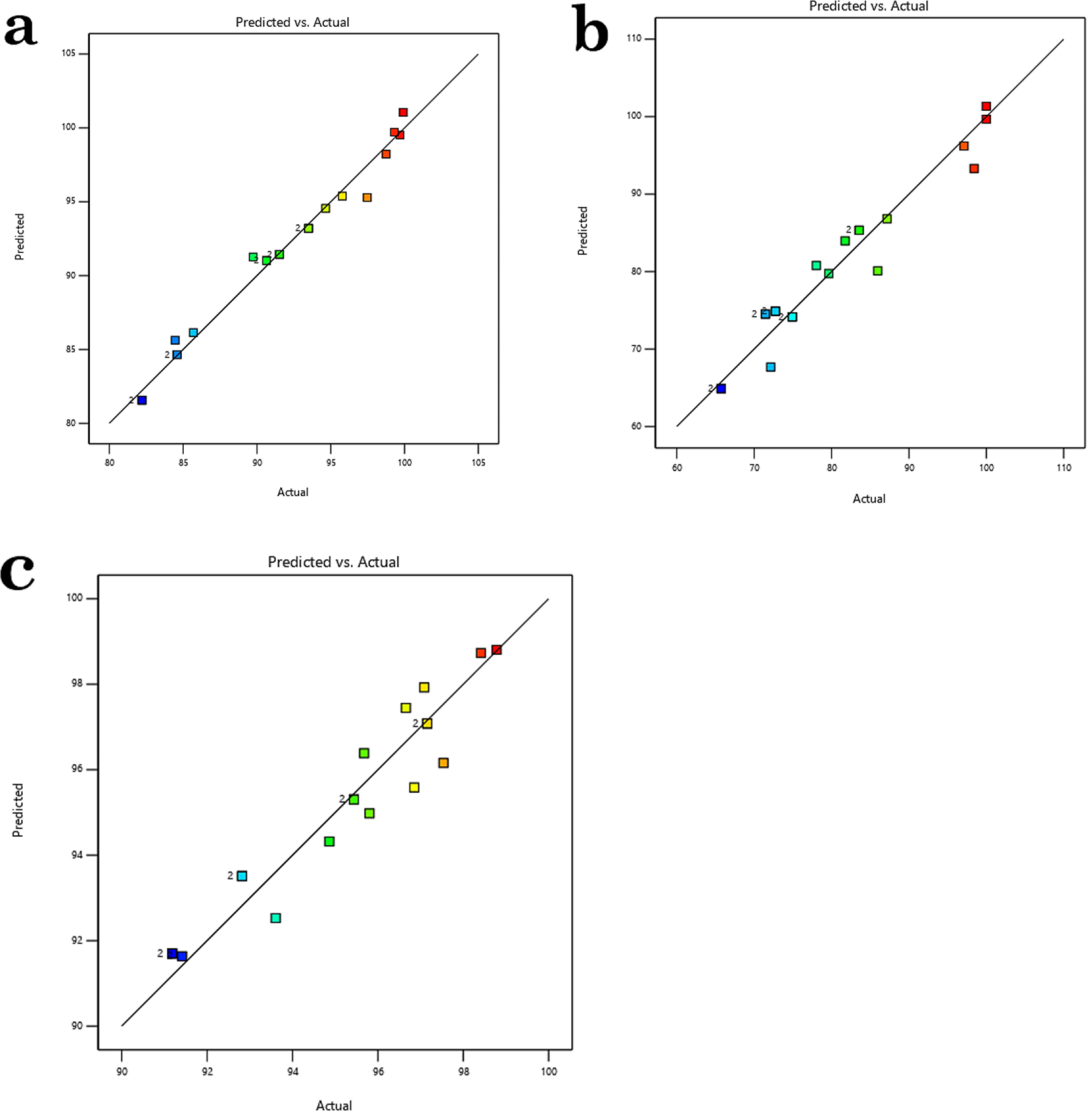
Plots between the predicted and experimental data for (**a**) AB14 dye adsorption, (**b**) AO7 dye adsorption, and (**c**) Cr^6+^ ions adsorption.

Figure 17 displays a link between the actual and expected percentages of AB14 dye, AO7 dye, and Cr^6+^ ions adsorbed on AC5-600. The image clearly shows that the actual data and the projected model coincide well. This is supported by the strong correlation coefficient values (*R*^2^ = 0.9824, 0.9426, and 0.9233) for the Cr^6+^ ions, AO7 dye, and AB14 dye, respectively. The cubic, individual, and interaction effects of the independent variables on the adsorption of Cr^6+^ ions, AO7 dye, and AB14 dye on AC5-600 are predicted using the ANOVA, which is presented in Table S3-S5. According to the results, there is a substantial contribution from the quadratic and 2FI models (P-value < 0.0001). The values of Adj-*R*^2^ = and *R*^2^ = demonstrate a strong correlation between the expected and exponential data, and the determination coefficient defined the standard of the polynomial model as a foundation of the extent of departure via the mean explained by the model^32,33^. The discrepancy between the Predicted *R*^2^ and the Adjusted *R*^2^ of Cr^6+^ ions, AO7 dye, and AB14 dye is less than 0.2, indicating a satisfactory agreement. The ratio of signal to noise (S/N) is measured by Adeq Precision. Ideally, the ratio should be higher than 4. For AB14 dye, AO7 dye, and Cr^6+^ ions, respectively, the S/N values of 57.87, 672.60, and 19.68 reveal a sufficient signal with a significant RSM model signal that may be used to navigate the design^32,34^.

#### Simultaneous effects of interactive adsorption variables

The impacts and interactions of three independent variables-A: CA5-600 dosage, B: starting dye concentration, and C: contact time on the removal percentages of Cr^6+^ ions, AO7 dye, and AB14 dye are displayed in three-dimensional surface plots as the responses (Figure S4-S6). Figures S4a, S5a, and S6a illustrate how the starting ion concentration and adsorbent dose combine to significantly impact the percentage of AB14 dye, AO7 dye, and Cr^6+^ ions removed. As the dosage of adsorbent was raised, the removal percentages rose as well. This outcome was brought about by the abundance of extra active sites and a sizable surface area of the adsorbent that is easily accessible for adsorption^31,35^. Figures S4c, S5c, and S6c show that by raising the starting ion concentration, the removal % was lowered. This observation may be explained by the small number of active sites present at high adsorbate concentrations on the adsorbent surface^36^. These findings demonstrated that the adsorbent surface’s vast surface area and unoccupied spots contributed to the initial high adsorption rate^37^. One possible reason for the slowdown in ion removal might be the challenge of getting to the remaining empty locations. A parallel statistical design calculation was carried out under identical experimental settings in order to optimise and validate the anticipated mathematical model; Figures S7-S9 demonstrate that the greater desirability value generated from the mathematical model’s results is equal to 1. The greatest removal (%) (99.93%) for AB14 dye under these conditions matches the contact time: The maximum % removal (100%) for AO7 dye attained in 79.7 min with an initial concentration of 102.3 mg/L and an AC5-600 dosage of 213.6 mg/100 mL matches the contact time: For Cr^6+^ ions, the greatest removal (%) (98.78% = experimental) achieved corresponds to the contact time: 116.7 min, AC5-600 dosage of 249.8 mg/100 mL, and beginning concentration of 104.4 mg/L. For AC5-600, the same parameters apply: 67.9 min, 223.4 mg/100 mL, and an initial concentration of 100.1 mg/L.

### ANN modelling

**Fig. 18:**
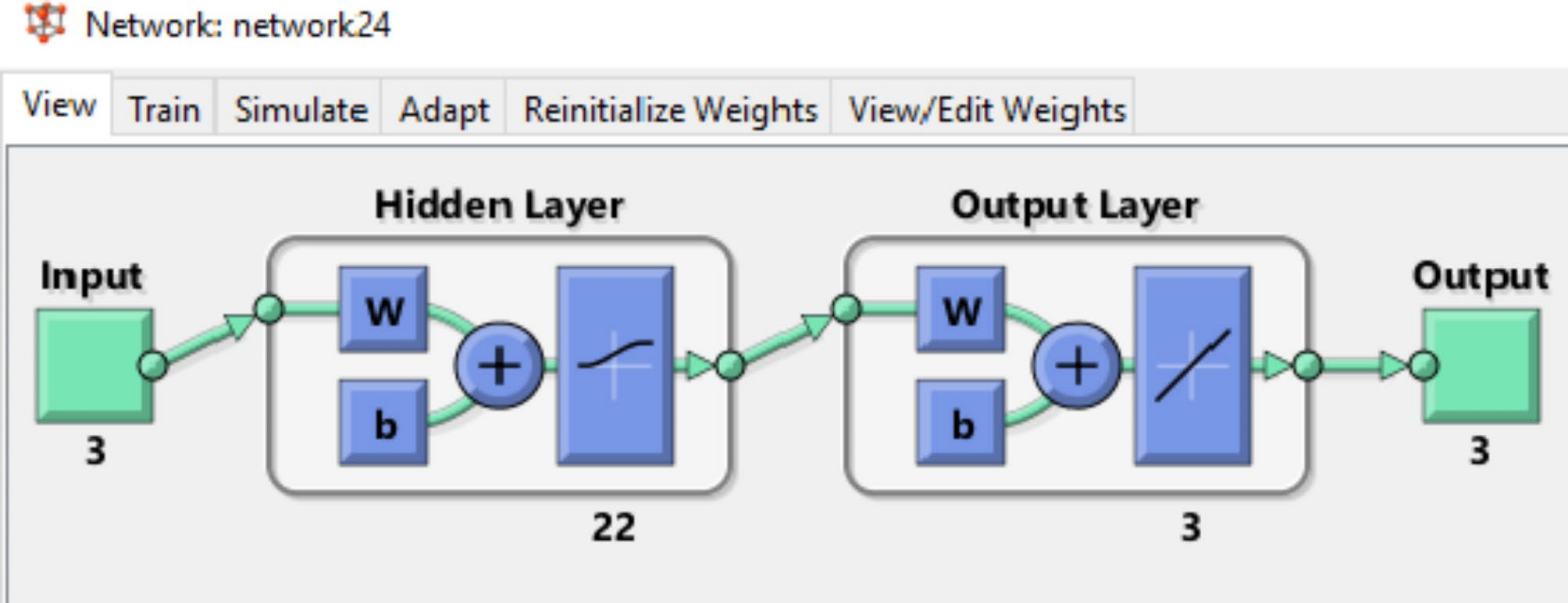
ANN architecture of the adsorption of AO7 dye, AB14 dye, and Cr^6+^ ions.

**Fig. 19:**
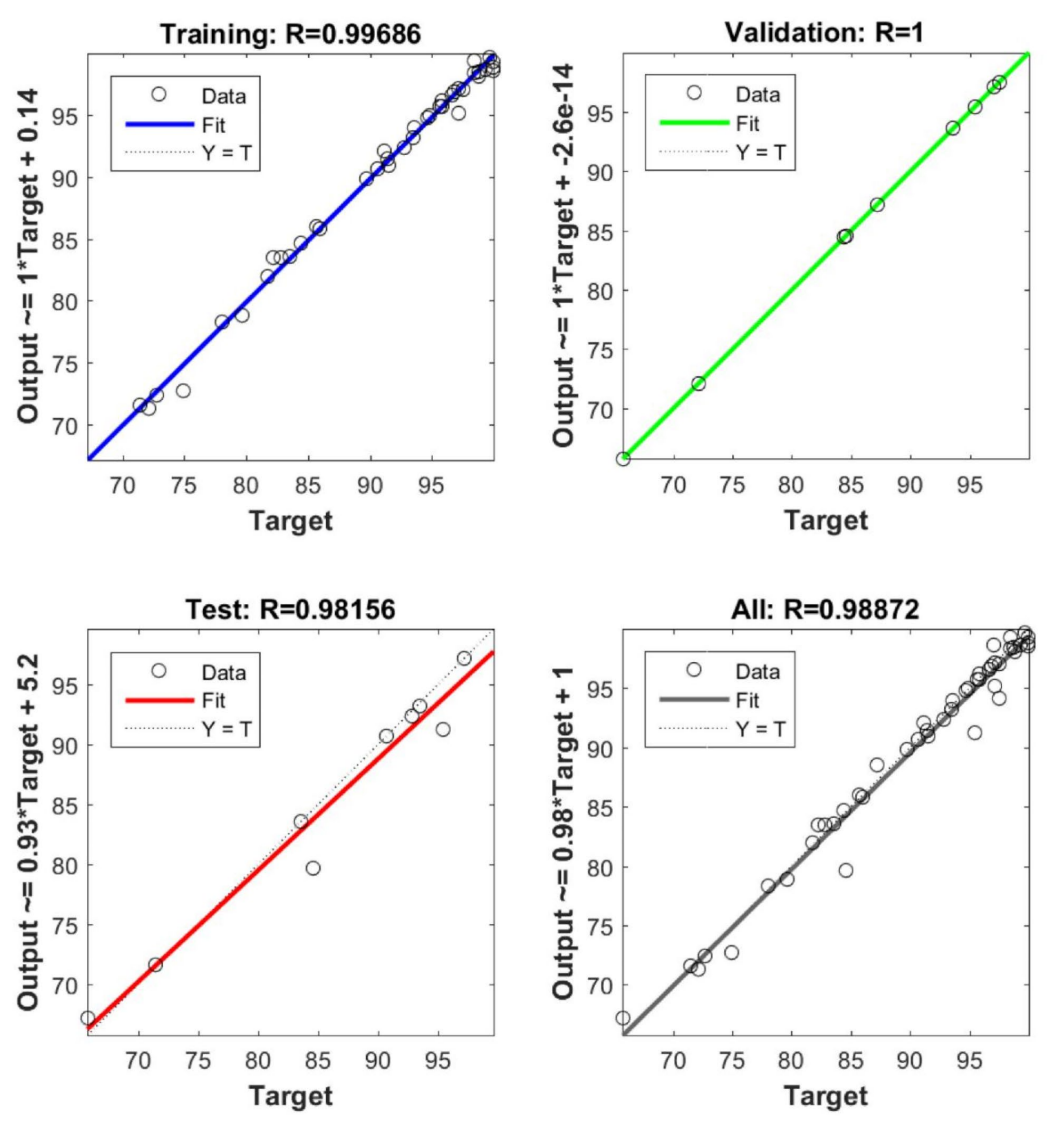
Training, validation, testing and overall datasets for the LM algorithm.

**Fig. 20:**
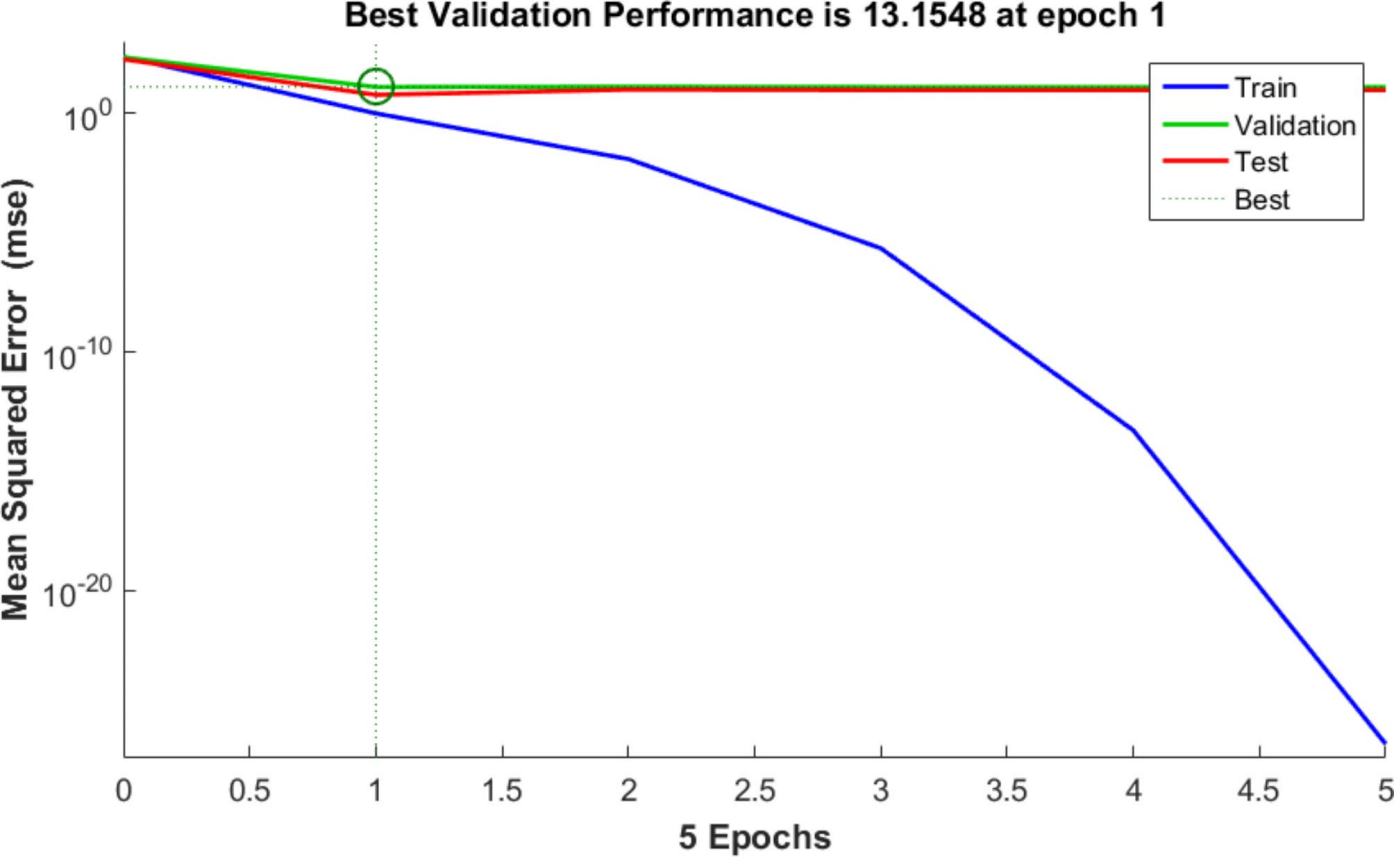
MSE error vs the epoch number for the optimized ANN model (LM algorithm performance).

For training, validation, and testing, the study’s sample data were split up at 70:15:15, correspondingly. Performance, performance gradient, validation checks number, and training epoch number were the four main training variables of the ANN. The highest *R*^2^ and the least MSE were used to classify the optimal ANN model architecture. The backpropagation algorithm (trainlm) was the training procedure. The best-fit network for the biosorption of AO7, AB14, and Cr^6+^ to AC5-600 was determined as 3-22-3 (3- ILs, 22- HLs, and 3 OL) as shown in Fig. 18. The regression plots displayed that the R^2^ was high with low MSE error values (R^2^ for training, validation, testing, and overall were 0.9967, 1.000, 0.9816, and 0.9887, respectively). These regression plots are shown in Fig. 19. The MSE value was 3.5e-27 and the results showed a good association between the training data of the optimized structure of the obtained ANN model. The performance of the ANN model in the testing and validation was perfect, although some data scatterings. In this study, 3 input variables (adsorbent dosage of AC5-600 (mg), time (min), and initial concentrations of AO7 dye, AB14 dye, and Cr^6+^ ions (mg/L)) and 3 output variables (removal % of AO7 dye, AB14 dye, and Cr^6+^ ions) were specified in the system. The best ANN followed the LM, which possessed 22 neurons in the hidden layer. The best ANN activation functions were Log-Sigmoid (log-sig) for the hidden layer and purelin for the output layer. The MSE error vs the epoch number for the optimized ANN model is shown in Fig. 20. For the best ANN, the best validation performance is 13.1548 reported at epoch 1 and the training process stopped after 1 epoch, showing that the maximum model training of the ANN was satisfactorily attained at 1 epoch for modelling the adsorption process^45,46,119^.

### Adsorption mechanism

**Fig. 21:**
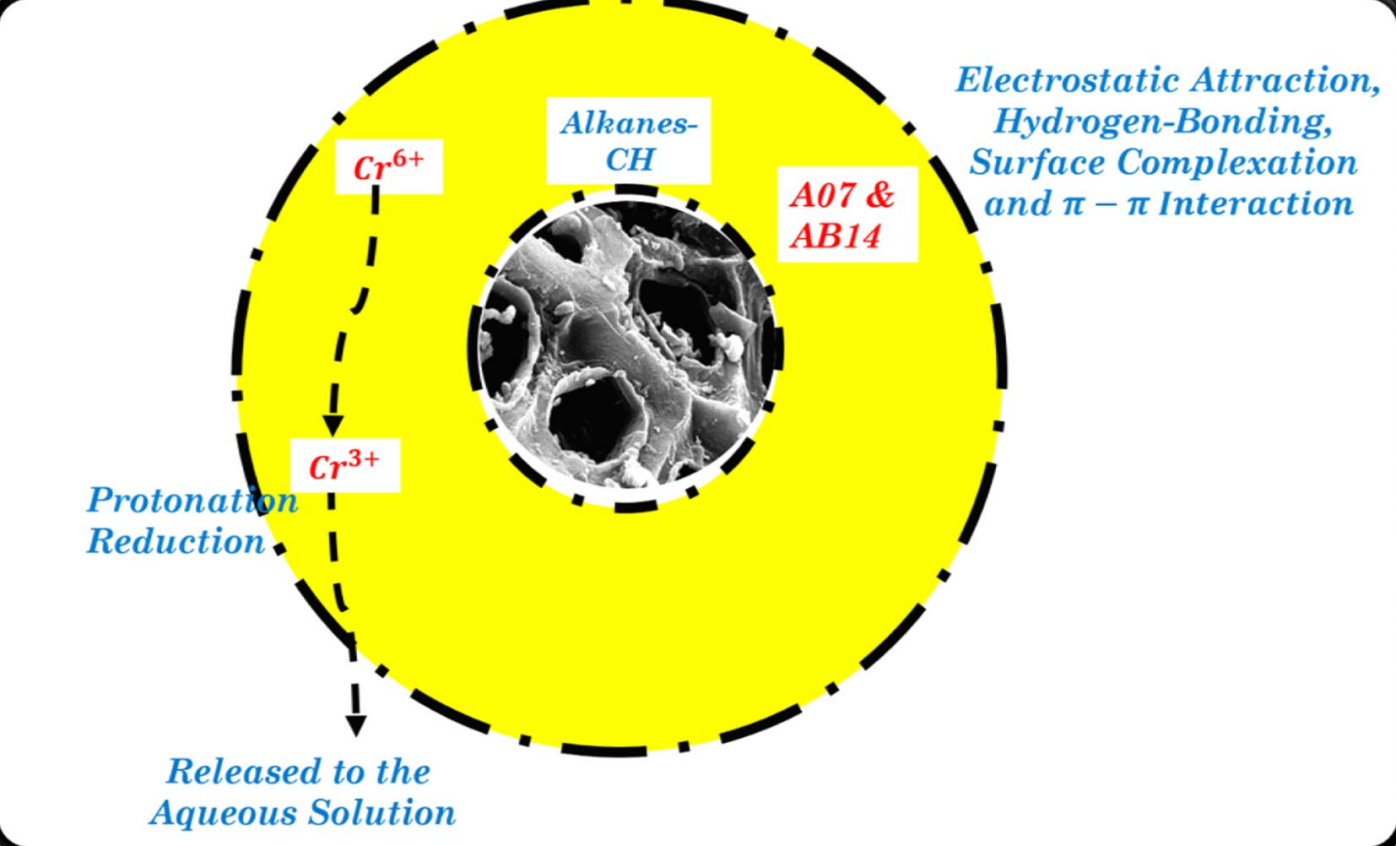
Possible adsorption mechanism of the sorption of AO7, AB14 and Cr^6+^ to AC5-600.

A more precise understanding of the sorption mechanism of AO7 dye, AB14 dye, and Cr^6+^ ions to AC5-600 is essential. This depends on the textural and surface properties of the AC5-600 adsorbent and how the sorbate diffuses towards the adsorbent, with a blend of chemisorption and physisorption driving the sorption process of AO7 and AB14 dye. As observed from the IR analysis, both anionic dye molecules were sorbed to the AC5-600 owing to the physical electrostatic interaction/attraction between H^+^ FGs such as alkane on the surface of the synthesized AC5-600 and the -vely charged sulfonate anion dye molecules through ion-exchange. These groups can draw species with opposite charges and repel those with similar charges. Furthermore, the removal of these dyes consists of hydrogen bonding and $\pi -\pi$ interaction as shown in Fig. 21^48^. Reduced removal of dye molecules at the alkaline pH values was attributed to the dual competition between the -vely charged sulfonate anion dye molecules and the -OH anions for the active surface sites on AC5-600^120^. AC with its graphitic structure can form $\pi -\pi$ interactions with the dye aromatic rings and this kind of interaction improves the removal of these dyes^92^. The mechanism of Cr^6+^ ions sorption to AC5-600 can be explained based on (a) sorption joined with reduction, (b) anionic sorption, (c) reduction and cationic sorption and (d) anionic and cationic sorption. Analyzing the above results, the Cr^6+^ sorption mechanism to AC5-600 comprises of the sorption attached reduction as shown in Fig. 21. From the IR analysis, the existence of alkane groups (CH) was involved in the Cr^6+^ sorption collective with the reduction process. This sorption mechanism advocates that the first three stages of Cr^6+^ sorption were from the bulk phase to the solid phase. Hence, the first stage of the swift sorption of Cr^6+^ ions to the functional active surface sites on AC5-600 was via the electrostatic attraction resulting from the surface site protonation with CH on AC5-600 and HCrO_4_^−^. Cr^6+^ ions were reduced to Cr^3+^ ions in a sturdily acidic medium providing excessive H^+^ for Cr reduction^93,121^.

#### Regeneration of AC5-600

**Fig. 22:**
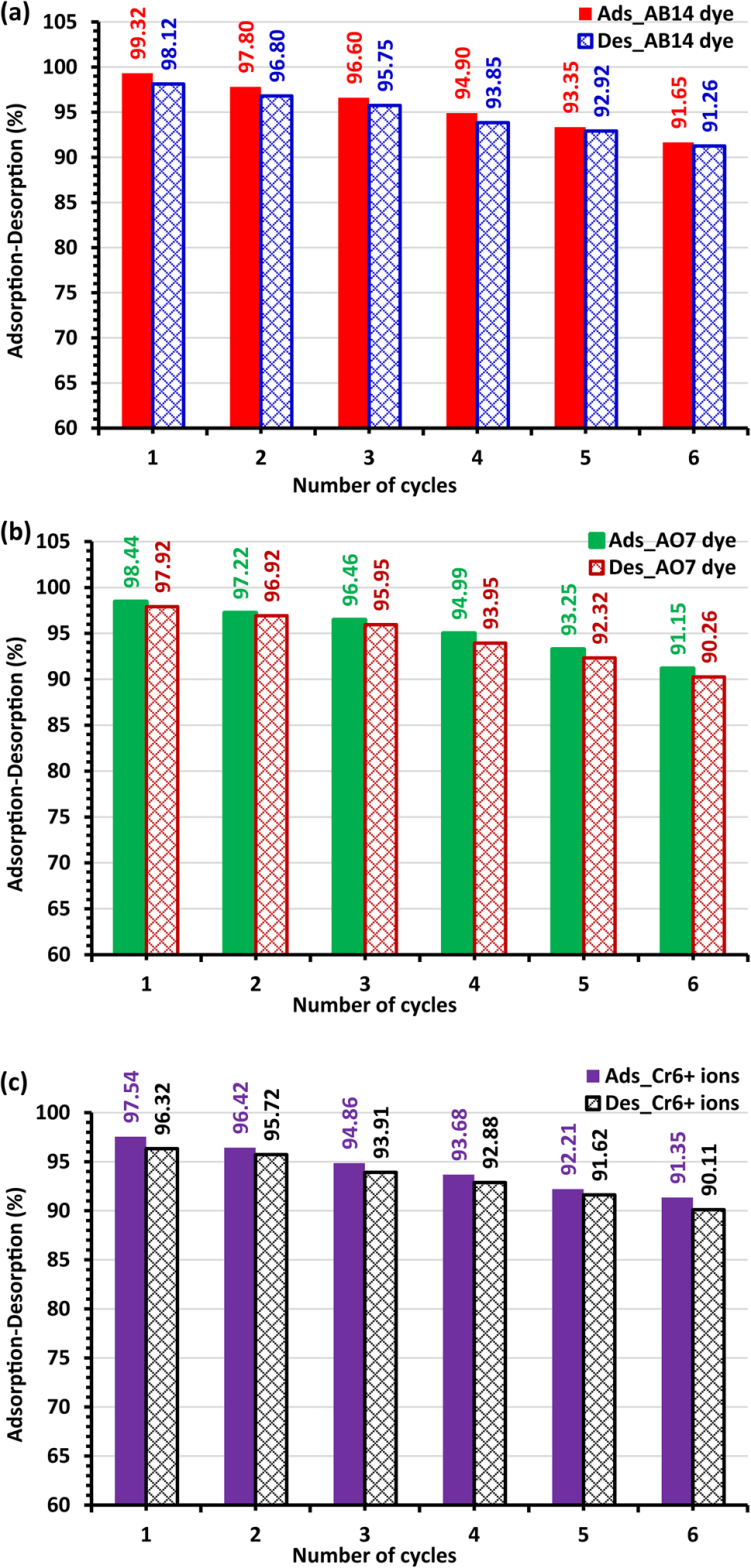
(**a**) Regeneration of AC5-600 (1.5 g L^–1^) for adsorption–desorption of (a) AB14 dye (100 mg L^–1^), (**b**) AO7 dye (100 mg L^–1^), and (**c**) Cr^6+^ ions (100 mg L^–1^) during 6 cycles at pH = 1.5.

To test the viability and reusability of AC5-600 as an adsorbent, desorption tests of the AO7 dye, AB14 dye and Cr^6+^ ions from the AC5-600 adsorbent were carried out by 0.1 M NaOH and HCl as elution media and regeneration. With increasing regeneration cycles in this situation, the desorption percentage dropped (Fig. 22). The regenerated AC5-600 was used in six successive adsorption/desorption cycles for the three pollutants. The amount of adsorption–desorption that was offered remained mainly constant during the six cycles. It implies that it might be employed for the Cr^6+^ ions and dyes adsorption process (Fig. 22)^122,123,124^.

## Conclusion

N-doped AC (AC5-600) was created from a blend of SD and grinded FW, which subsequently underwent hydrothermal treatment at 180 °C in the presence of urea and zinc chloride (ZnCl_2_). The prepared AC5-600 was assessed for the sorption of two dyes (AB14 and AO7 dyes) and HM (Cr^6+^ ions). The sorption of AB14 dye, AO7 dye and Cr^6+^ ions was found to be pH dependent, with the effective % elimination of AB14 dye, AO7 dye and Cr^6+^ ions observed at pH 1.5 and was attributed to the EI between the Cr^6+^ species and anion dyes and the protonated sites accessible on the surface of AC5-600 adsorbent at this pH. Based on the determined error values from the NL modelling (NLM) of the kinetic and isotherm models, the Elovich (ELM-AB14 dye and Cr^6+^ ions), PFOM-AB14 dye and second orders (PSOM-AB14 dye, AO7 dye and Cr^6+^ ions) and the FRHM were found to preferably explain the sorption of the various contaminants to AC5-600. The maximum sorption capacities of AB14 dye, AO7 dye and Cr^6+^ ions based on the NLM were 1114, 1929 and 318 mg g^-1^, respectively. Based on the computational adsorption calculations, the sorption energies for the AO7 and AB14 dyes were -4.492 and -8.090 eV and 2.563, 1.789, 1.226 and 1.928 eV for Cr_2_, CrO_3_, CrO_4_, and CrO_4_H species, respectively. These were indications of strong exothermic and endothermic absorption properties. The % of AB14 and AO7 dyes and Cr^6+^ ions sorbed by AC5-600 was predicted using the RSM, and ANN models. The ANN model was found to be highly applicable and effective in predicting the adsorption process of AB14 and AO7 dyes and Cr^6+^ ions to AC5-600 than the RSM model in this study. Future studies will focus on the use of AC5-600 adsorbent in the treatment of real wastewater containing AB14 and AO7 dye molecules and Cr^6+^ ions.

Furthermore, pilot-scale research and testing the adsorbent under actual wastewater conditions would aid in assessing this present study’s potential. Finally, to ensure the sustainability and safety of the biosorption process for wastewater treatment, it is essential to investigate the possible toxicity and environmental impact of the toxic metal (such as Cr^6+^) ions and dyes (such as AO7 and AB14) adsorbents.

## Electronic supplementary material

Below is the link to the electronic supplementary material.

Supplementary Material 1.

## Data Availability

Data will be available upon request from the corresponding author.

## Abbreviations

AB14: Acid Brown 14
AC: Activated Carbon
AO7: Acid Orange 7
ANOVA: Analysis of variance
ANN: Artificial neural network
C: Carbon
Cr: Chromium
ELM: Elovich
C_e_: Equilibrium concentration
FW: Fish waste
FRHM: Freundlich model
FGs: Functional groups
HMs: Heavy metals
Cr^6+^: Hexavalent chromium
C_o_: Initial concentration
LNRM: Langmuir model
LM: Levenberg Marquart
LM: Linear modelling
*Q*_m_: Maximum sorption capacity
N: Nitrogen
NLM: Non-linear modelling
PFOM: Pseudo-first-order model
PSOM: Pseudo-second order model
RSM: Response surface methodology
SD: Sawdust
TEKM: Temkin model
