# Supplementary material for: Modelling of a new form of nitrogen doped activated carbon for adsorption of various dyes and hexavalent chromium ions
Source: Sci Rep. 2025 Jan 31;15:3896. doi: 10.1038/s41598-025-87398-6 (PMC11785975; doi:10.1038/s41598-025-87398-6)
Supplement: Supplementary file 1 — Supplementary Material 1. [file 41598_2025_87398_MOESM1_ESM.docx]

**Supplementary Data**

**Modelling of a new form of nitrogen doped activated carbon for adsorption of various dyes and hexavalent chromium ions**

Mohamed A. El-Nemr^1^, Uyiosa Osagie Aigbe^2^, Kingsley  Eghonghon Ukhurebor^3^, Kingsley Obodo^4^, Adetunji Ajibola Awe^5^, Mohamed A. Hassaan^6^, Safaa Ragab^6^, Ahmed El Nemr^6*^

^1^Department of Chemical Engineering, Faculty of Engineering, Minia University, Minia, Egypt

^2^Department of Mathematics and Physics, Cape Peninsula University of Technology, Cape Town, South Africa

^3^Department of Physics, Faculty of Science, Edo State University Uzairue, Edo State, Nigeria

^4^Center for Space Research, North-West University, Potchefstroom, 2531, South Africa

^5^Department of Conservation and Marine Sciences, Cape Peninsula University of Technology, Cape Town, South Africa

^6^Environment Division, National Institute of Oceanography and Fisheries, Kayet Bey, El-Anfoushy, Alexandria, Egypt

E-mail: [mohamedelnemr1992@yahoo.com](mailto:mohamedelnemr1992@yahoo.com) (M.A. El-Nemr); [uyi4we@gmail.com](mailto:uyi4we@gmail.com) (U.O. Aigbe), [ukeghonghon@gmail.com](mailto:ukeghonghon@gmail.com) (K.E. Ukhurebor), obodokingsleyo@gmail.com (K. Obodo), awea@cput.ac.za (A.A. Awe), [safaa_ragab65@yahoo.com](mailto:safaa_ragab65@yahoo.com) (S. Ragab), [mhss95@mail.com](mailto:mhss95@mail.com) (M.A. Hassaan)

^*^Corresponding author: E-mail [ahmedmoustafaelnemr@yahoo.com](mailto:ahmedmoustafaelnemr@yahoo.com); ahmed.m.elnemr@gmail.com (A. El Nemr)

| 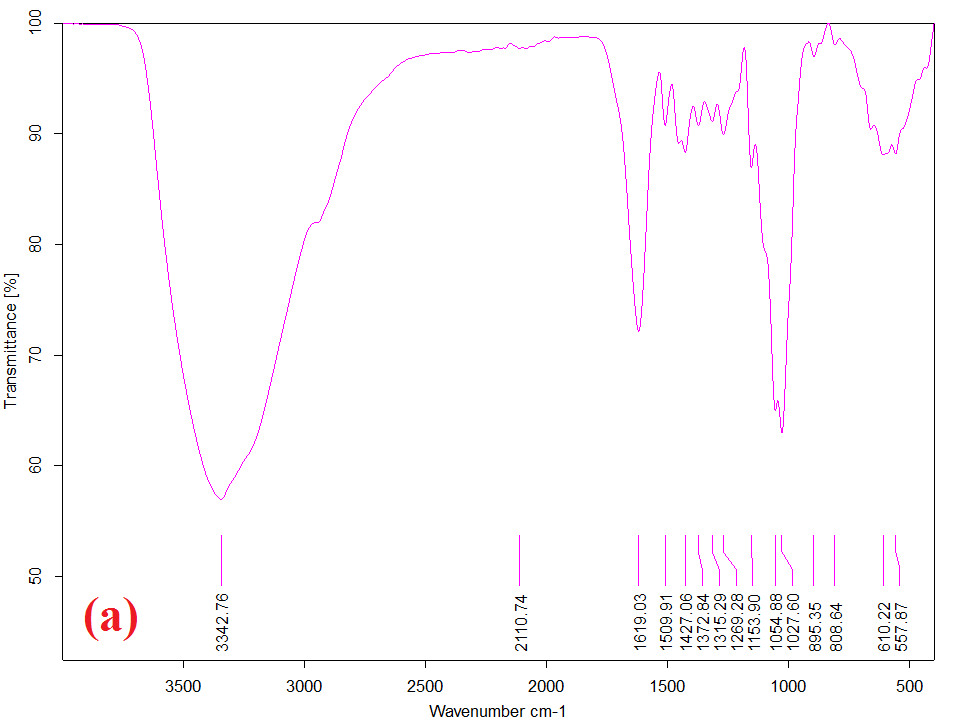 |
| --- |
| 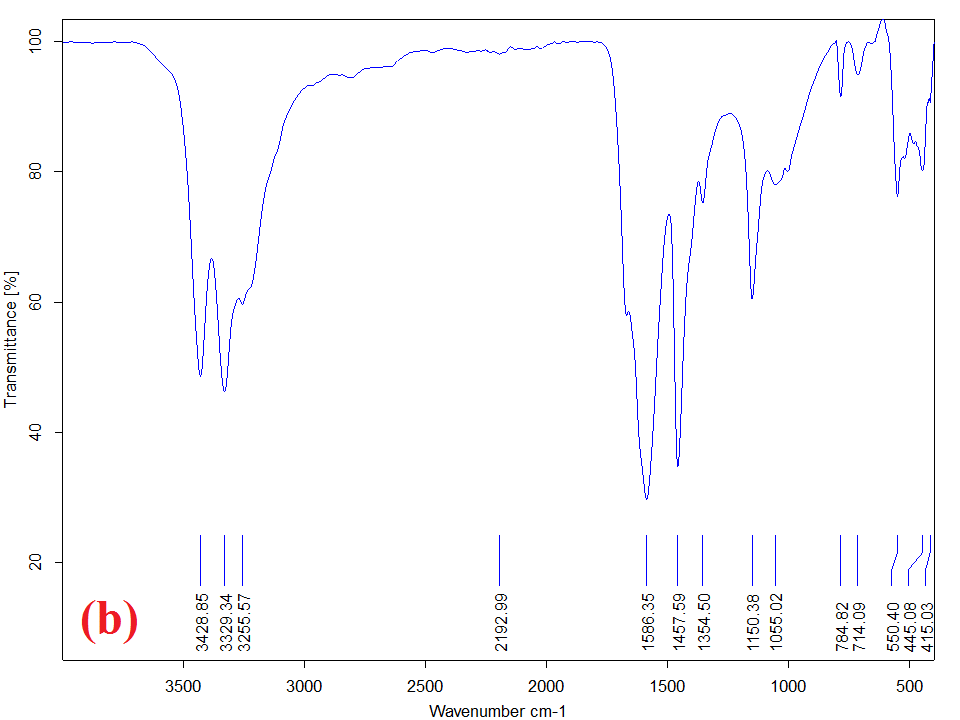 |
| 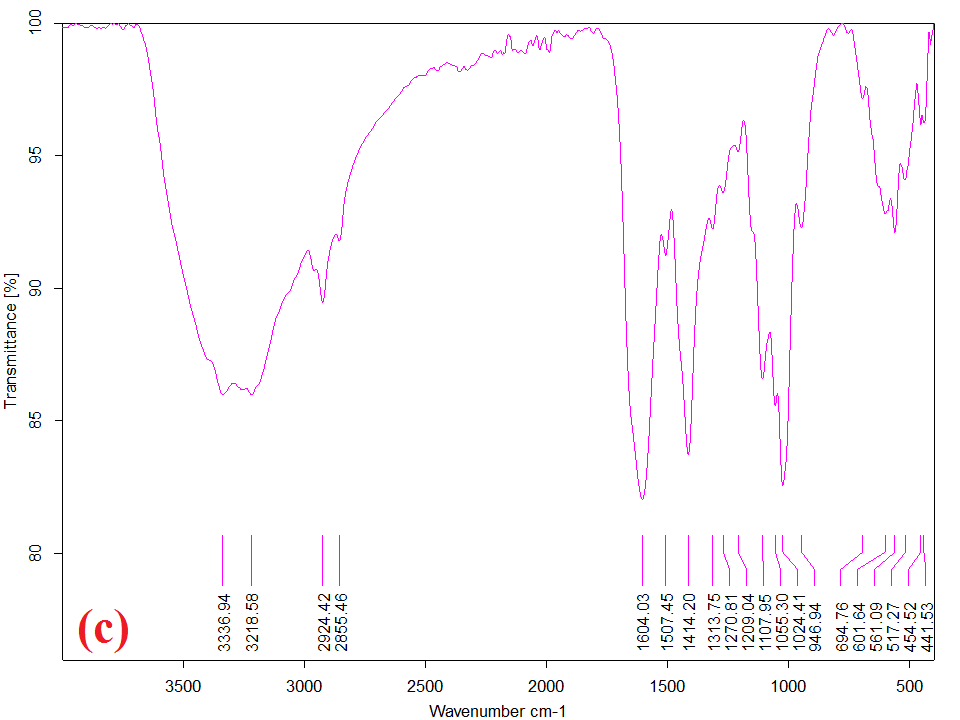 |
| 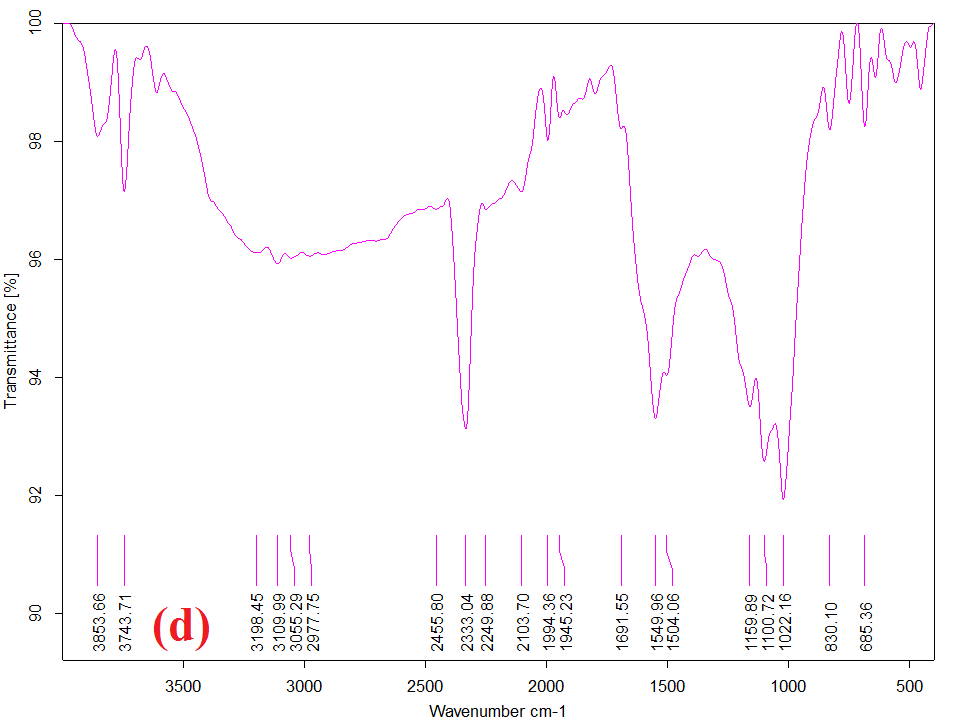 |
| 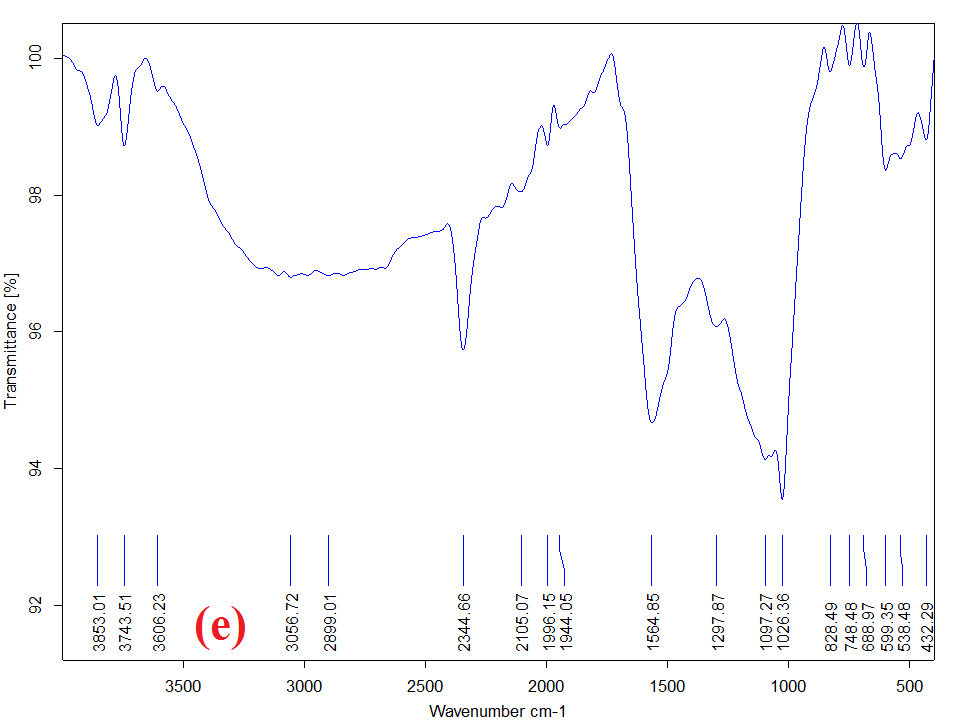 |

**Figure S1**. FTIR analysis of (a) Saw-dust-ZnCl_2_, (b) Urea, (c) Fish-SD-Urea-ZnCl_2_, (d) AC5-600-AO7 dye, (e) AC5-600-AB14 dye.

| 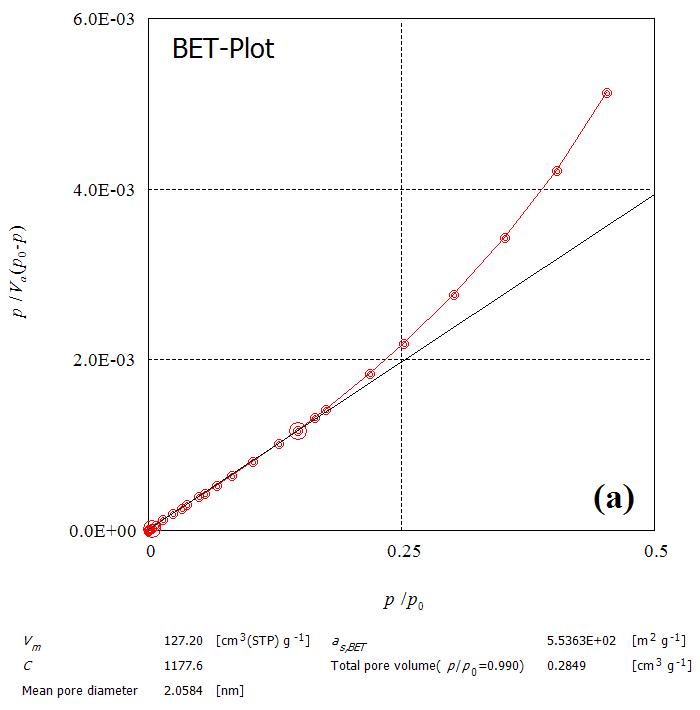 |
| --- |
| 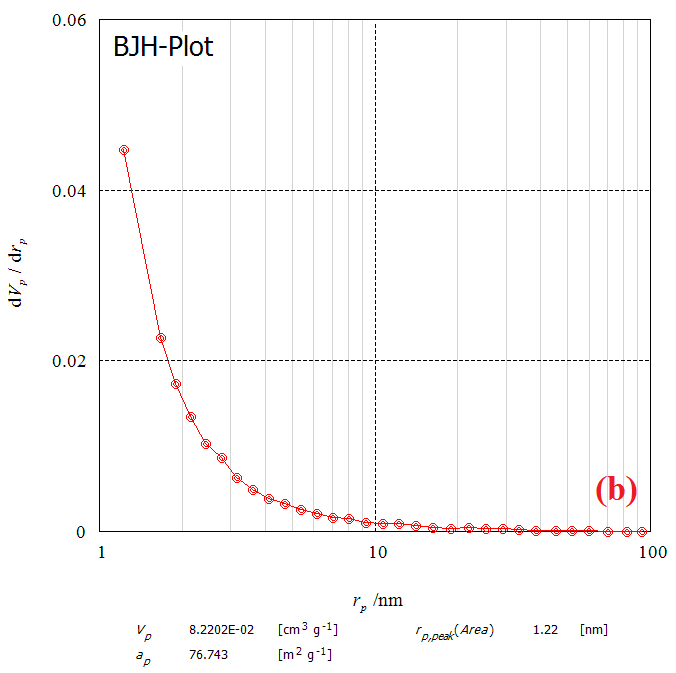 |
| 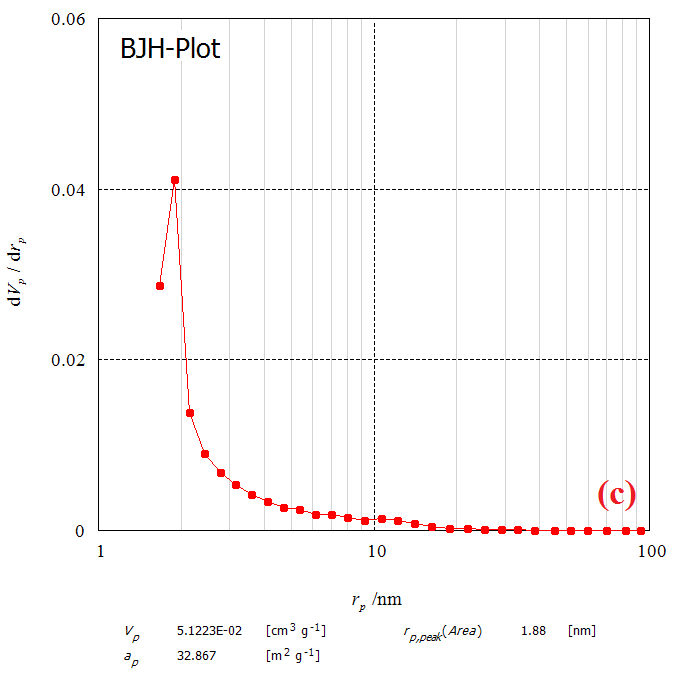 |
| 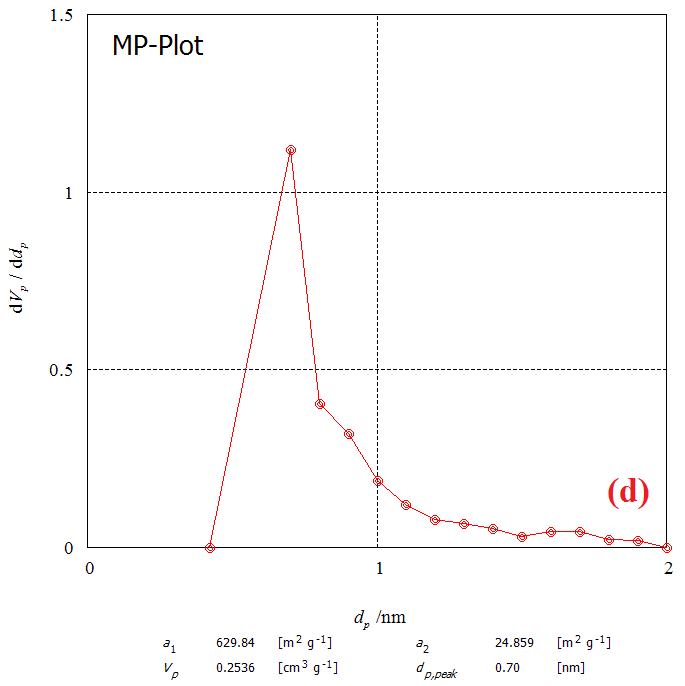 |
| 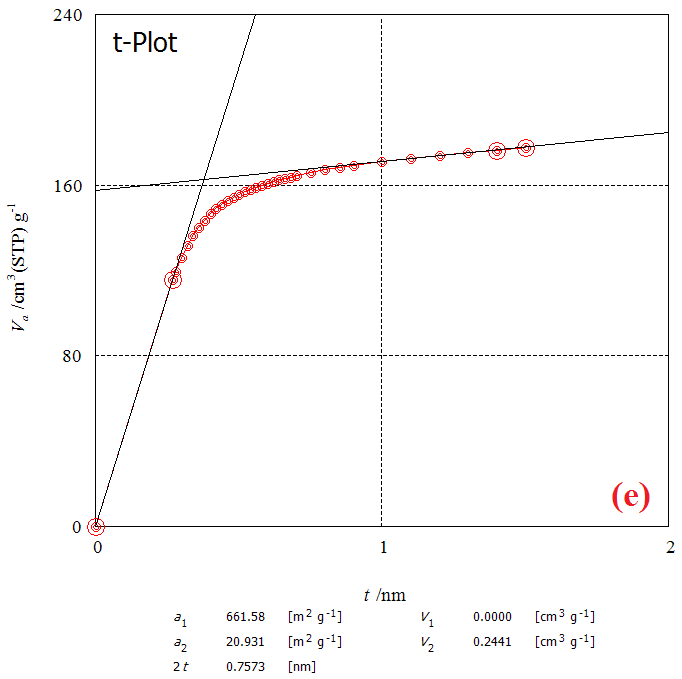 |

**Figure S2**. Surface area analysis of N-doped activated carbon AC5-600 (a) BET plot, (b) BJH adsorption plot, (c) BJH desorption plot, (d) Micropore (MP) plot, and *t*-plot analyses.


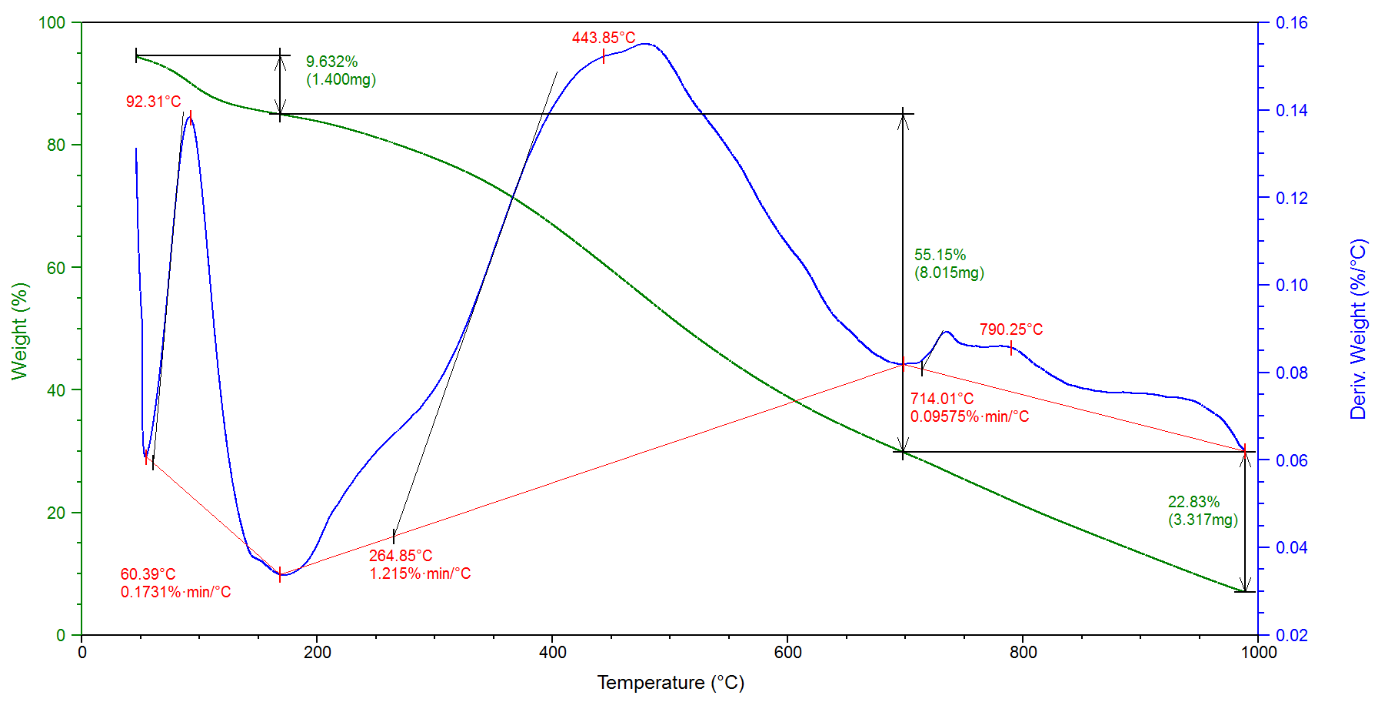


**Figure S3**. TGA-DTA analysis of Saw dust raw material from 50 to 1000 °C.

**Table S1.** Range and levels used for the batch desorption study.

| **Factor** | **Name** | **Units** | **Type** | **SubType** | **Minimum** | **Maximum** | **Coded Low** | **Coded High** | **Mean** | **Std. Dev.** |
| --- | --- | --- | --- | --- | --- | --- | --- | --- | --- | --- |
| A | Adsorbent dosage | mg | Numeric | Discrete | 50.00 | 250.00 | -1 ↔ 50.00 | +1 ↔ 250.00 | 145.00 | 87.21 |
| B | Dye Conc. | mg/L | Numeric | Discrete | 100.00 | 400.00 | -1 ↔ 100.00 | +1 ↔ 400.00 | 240.00 | 133.38 |
| C | Time | min | Numeric | Discrete | 15.00 | 120.00 | -1 ↔ 15.00 | +1 ↔ 120.00 | 70.50 | 45.16 |

**Table S2.** Experimental design for adsorption of AB14, AO7 and Cr (VI) on AC5-600.

|  | **Factor 1** | **Factor 2** | **Factor 3** | **Response 1**  **AB14** | | **Response 1**  **AO7** | | **Response 1**  **Cr (VI)** | |
| --- | --- | --- | --- | --- | --- | --- | --- | --- | --- |
| Run | **A:Adsorbent dosage** | **B:Dye Conc.** | **C:Time** | **Removal** | **PREDICTED** | **Removal** | **PREDICTED** | **Removal** | **PREDICTED** |
|  | mg | mg/L | min | % | % | *%* | *%* | % | % |
| 1 | 50 | 400 | 120 | 84.5954 | 84.65 | 65.7348 | 64.89 | 95.4399 | 95.30 |
| 2 | 250 | 200 | 15 | 94.6603 | 94.55 | 81.755 | 83.97 | 96.6579 | 97.45 |
| 3 | 150 | 100 | 120 | 99.3154 | 99.71 | 98.4361 | 93.31 | 97.5377 | 96.16 |
| 4 | 100 | 400 | 15 | 82.2108 | 81.56 | 72.751 | 74.88 | 91.185 | 91.70 |
| 5 | 250 | 400 | 90 | 90.6519 | 91.02 | 83.5716 | 85.34 | 97.1572 | 97.08 |
| 6 | 50 | 100 | 120 | 93.4988 | 93.20 | 71.461 | 74.52 | 92.819 | 93.51 |
| 7 | 50 | 100 | 15 | 91.5326 | 91.43 | 74.936 | 74.15 | 91.4135 | 91.63 |
| 8 | 250 | 250 | 90 | 97.4791 | 95.29 | 87.1732 | 86.81 | 97.0896 | 97.93 |
| 9 | 150 | 100 | 60 | 98.7594 | 98.22 | 97.1303 | 96.21 | 96.856 | 95.59 |
| 10 | 250 | 100 | 120 | 99.9181 | 101.05 | 99.999 | 99.67 | 98.7847 | 98.81 |
| 11 | 50 | 400 | 120 | 84.5954 | 84.65 | 65.7348 | 64.89 | 95.4399 | 95.30 |
| 12 | 150 | 250 | 120 | 95.7938 | 95.39 | 79.6541 | 79.74 | 95.6809 | 96.39 |
| 13 | 250 | 400 | 90 | 90.6519 | 91.02 | 83.5716 | 85.34 | 97.1572 | 97.08 |
| 14 | 50 | 100 | 120 | 93.4988 | 93.20 | 71.461 | 74.52 | 92.819 | 93.51 |
| 15 | 50 | 100 | 15 | 91.5326 | 91.43 | 74.936 | 74.15 | 91.4135 | 91.63 |
| 16 | 250 | 400 | 15 | 85.6926 | 86.14 | 85.9806 | 80.11 | 95.8058 | 94.98 |
| 17 | 50 | 250 | 60 | 84.4579 | 85.63 | 72.135 | 67.68 | 93.6064 | 92.53 |
| 18 | 250 | 100 | 60 | 99.7016 | 99.53 | 99.999 | 101.34 | 98.4148 | 98.73 |
| 19 | 100 | 400 | 15 | 82.2108 | 81.56 | 72.751 | 74.88 | 91.185 | 91.70 |
| 20 | 150 | 250 | 30 | 89.7431 | 91.27 | 78.0323 | 80.79 | 94.864 | 94.32 |

**Table S3.** ANOVA and model fit summary for D-optimal design for AB14 adsorption

| **Source** | **Value** | **Sum of Squares** | **df** | **Mean Square** | **F-value** | **p-value** | **Remarks** | **source** | **SD** | **R²** | **Adjusted R²** | **Predicted R²** | **PRESS** | **Remarks** |
| --- | --- | --- | --- | --- | --- | --- | --- | --- | --- | --- | --- | --- | --- | --- |
| **Model** | - | 667.87 | 9 | 74.21 | 62.07 | < 0.0001 | significant | - | - | - | - | - | - | - |
| A-Adsorbent dosage | - | 179.53 | 1 | 179.53 | 150.15 | < 0.0001 | - | - | - | - | - | - | - | - |
| B-Dye Conc. | - | 394.57 | 1 | 394.57 | 330.02 | < 0.0001 | - | - | - | - | - | - | - | - |
| C-Time | - | 61.98 | 1 | 61.98 | 51.84 | < 0.0001 | - | - | - | - | - | - | - | - |
| AB | - | 0.6047 | 1 | 0.6047 | 0.5058 | 0.4932 | - | - | - | - | - | - | - | - |
| AC | - | 0.0093 | 1 | 0.0093 | 0.0077 | 0.9316 | - | - | - | - | - | - | - | - |
| BC | - | 15.25 | 1 | 15.25 | 12.76 | 0.0051 | - | - | - | - | - | - | - | - |
| A² | - | 18.01 | 1 | 18.01 | 15.07 | 0.0031 | - | - | - | - | - | - | - | - |
| B² | - | 0.2614 | 1 | 0.2614 | 0.2186 | 0.6501 | - | - | - | - | - | - | - | - |
| C² | - | 0.4833 | 1 | 0.4833 | 0.4042 | 0.5392 | - | - | - | - | - | - | - | - |
| **Residual** | - | 11.96 | 10 | 1.20 |  |  | - | - | - | - | - | - | - | - |
| Lack of Fit | - | 11.96 | 5 | 2.39 |  |  | - | - | - | - | - | - | - | - |
| Pure Error | - | 1.353E-09 | 5 | 2.705E-10 |  |  | - | - | - | - | - | - | - | - |
| **Cor Total** | - | 679.83 | 19 |  |  |  |  | - | - | - | - | - | - | - |
| **SD** | 1.09 | - | - | - | - | - | - | - | - | - | - | - | - | - |
| **Mean** | 91.53 | - | - | - | - | - | - | - | - | - | - | - | - | - |
| **C.V.%** | 1.19 | - | - | - | - | - | - | - | - | - | - | - | - | - |
| **R²** | 0.9824 | - | - | - | - | - | - | - | - | - | - | - | - | - |
| **Adjusted R²** | 0.9666 | - | - | - | - | - | - | - | - | - | - | - | - | - |
| **Predicted R²** | 0.9149 | - | - | - | - | - | - | - | - | - | - | - | - | - |
| **Adeq Precision** | 25.2087 | - | - | - | - | - | - | - | - | - | - | - | - | - |
| **Linear** |  |  |  |  |  |  |  |  | 1.54 | 0.9439 | 0.9334 | 0.9175 | 56.07 | **Suggested** |
| 2FI |  |  |  |  |  |  |  |  | 1.53 | 0.9552 | 0.9346 | 0.9038 | 65.43 |  |
| **Quadratic** |  |  |  |  |  |  |  |  | 1.09 | 0.9824 | 0.9666 | 0.9149 | 57.87 | **Suggested** |
| Cubic |  |  |  |  |  |  |  |  | 0.0000 | 1.0000 | 1.0000 |  | * | Aliased |

**Table S4.** ANOVA and model fit summary for D-optimal design for AO7 adsorption

| **Source** | **Value** | **Sum of Squares** | **df** | **Mean Square** | **F-value** | **p-value** | **Remarks** | **source** | **SD** | **R²** | **Adjusted R²** | **Predicted R²** | **PRESS** | **Remarks** |
| --- | --- | --- | --- | --- | --- | --- | --- | --- | --- | --- | --- | --- | --- | --- |
| **Model** | - | 2176.70 | 9 | 241.86 | 18.24 | < 0.0001 | significant | - | - | - | - | - | - | - |
| A-Time | - | 963.94 | 1 | 963.94 | 72.68 | < 0.0001 | - | - | - | - | - | - | - | - |
| B-Dose | - | 510.54 | 1 | 510.54 | 38.50 | 0.0001 | - | - | - | - | - | - | - | - |
| C-Conc | - | 5.18 | 1 | 5.18 | 0.3903 | 0.5461 | - | - | - | - | - | - | - | - |
| AB | - | 37.46 | 1 | 37.46 | 2.82 | 0.1238 | - | - | - | - | - | - | - | - |
| AC | - | 9.12 | 1 | 9.12 | 0.6878 | 0.4263 | - | - | - | - | - | - | - | - |
| BC | - | 2.98 | 1 | 2.98 | 0.2250 | 0.6455 | - | - | - | - | - | - | - | - |
| A² | - | 104.25 | 1 | 104.25 | 7.86 | 0.0187 | - | - | - | - | - | - | - | - |
| B² | - | 142.09 | 1 | 142.09 | 10.71 | 0.0084 | - | - | - | - | - | - | - | - |
| C² | - | 47.00 | 1 | 47.00 | 3.54 | 0.0892 | - | - | - | - | - | - | - | - |
| **Residual** | - | 132.62 | 10 | 13.26 |  |  | - | - | - | - | - | - | - | - |
| Lack of Fit | - | 132.62 | 5 | 26.52 |  |  | - | - | - | - | - | - | - | - |
| Pure Error | - | 0.0000 | 5 | 0.0000 |  |  | - | - | - | - | - | - | - | - |
| **Cor Total** | - | 2309.33 | 19 |  |  |  | - | - | - | - | - | - | - | - |
| **SD** | 3.64 | - | - | - | - | - | - | - | - | - | - | - | - | - |
| **Mean** | 80.86 | - | - | - | - | - | - | - | - | - | - | - | - | - |
| **C.V.%** | 4.50 | - | - | - | - | - | - | - | - | - | - | - | - | - |
| **R²** | 0.9426 | - | - | - | - | - | - | - | - | - | - | - | - | - |
| **Adjusted R²** | 0.8909 | - | - | - | - | - | - | - | - | - | - | - | - | - |
| **Predicted R²** | 0.7087 | - | - | - | - | - | - | - | - | - | - | - | - | - |
| **Adeq Precision** | 14.1557 | - | - | - | - | - | - | - | - | - | - | - | - | - |
| **Linear** |  |  |  |  |  |  |  |  | 5.53 | 0.7885 | 0.7488 | 0.6858 | 725.56 |  |
| 2FI |  |  |  |  |  |  |  |  | 5.27 | 0.8434 | 0.7712 | 0.6270 | 861.27 |  |
| **Quadratic** |  |  |  |  |  |  |  |  | 3.64 | 0.9426 | 0.8909 | 0.7087 | 672.60 | **Suggested** |
| Cubic |  |  |  |  |  |  |  |  | 0.0000 | 1.0000 | 1.0000 |  | * | Aliased |

**Table S5.** ANOVA and model fit summary for D-optimal design for Cr (VI) adsorption

| **Source** | **Value** | **Sum of Squares** | **df** | **Mean Square** | **F-value** | **p-value** | **Remarks** | **source** | **SD** | **R²** | **Adjusted R²** | **Predicted R²** | **PRESS** | **Remarks** |
| --- | --- | --- | --- | --- | --- | --- | --- | --- | --- | --- | --- | --- | --- | --- |
| **Model** | - | 110.80 | 6 | 18.47 | 26.06 | < 0.0001 | significant | - | - | - | - | - | - | - |
| A-Time | - | 79.27 | 1 | 79.27 | 111.89 | < 0.0001 | - | - | - | - | - | - | - | - |
| B-Dose | - | 3.12 | 1 | 3.12 | 4.40 | 0.0561 | - | - | - | - | - | - | - | - |
| C-Conc | - | 17.96 | 1 | 17.96 | 25.35 | 0.0002 | - | - | - | - | - | - | - | - |
| AB | - | 4.90 | 1 | 4.90 | 6.91 | 0.0208 | - | - | - | - | - | - | - | - |
| AC | - | 1.66 | 1 | 1.66 | 2.35 | 0.1496 | - | - | - | - | - | - | - | - |
| BC | - | 5.38 | 1 | 5.38 | 7.59 | 0.0164 | - | - | - | - | - | - | - | - |
| **Residual** | - | 9.21 | 13 | 0.7085 |  |  | - | - | - | - | - | - | - | - |
| Lack of Fit | - | 9.21 | 8 | 1.15 |  |  | - | - | - | - | - | - | - | - |
| Pure Error | - | 0.0000 | 5 | 0.0000 |  |  | - | - | - | - | - | - | - | - |
| **Cor Total** | - | 120.01 | 19 |  |  |  | - | - | - | - | - | - | - | - |
| **SD** | 0.8417 | - | - | - | - | - | - | - | - | - | - | - | - | - |
| **Mean** | 95.07 | - | - | - | - | - | - | - | - | - | - | - | - | - |
| **C.V.%** | 0.8854 | - | - | - | - | - | - | - | - | - | - | - | - | - |
| **R²** | 0.9233 | - | - | - | - | - | - | - | - | - | - | - | - | - |
| **Adjusted R²** | 0.8878 | - | - | - | - | - | - | - | - | - | - | - | - | - |
| **Predicted R²** | 0.8360 | - | - | - | - | - | - | - | - | - | - | - | - | - |
| **Adeq Precision** | 14.4042 | - | - | - | - | - | - | - | - | - | - | - | - | - |
| **Linear** |  |  |  |  |  |  |  |  | 1.15 | 0.8250 | 0.7922 | 0.7235 | 33.19 |  |
| 2FI |  |  |  |  |  |  |  |  | 0.8417 | 0.9233 | 0.8878 | 0.8360 | 19.68 | **Suggested** |
| **Quadratic** |  |  |  |  |  |  |  |  | 0.8098 | 0.9454 | 0.8962 | 0.6916 | 37.01 |  |
| Cubic |  |  |  |  |  |  |  |  | 0.0000 | 1.0000 | 1.0000 |  | * | Aliased |

| 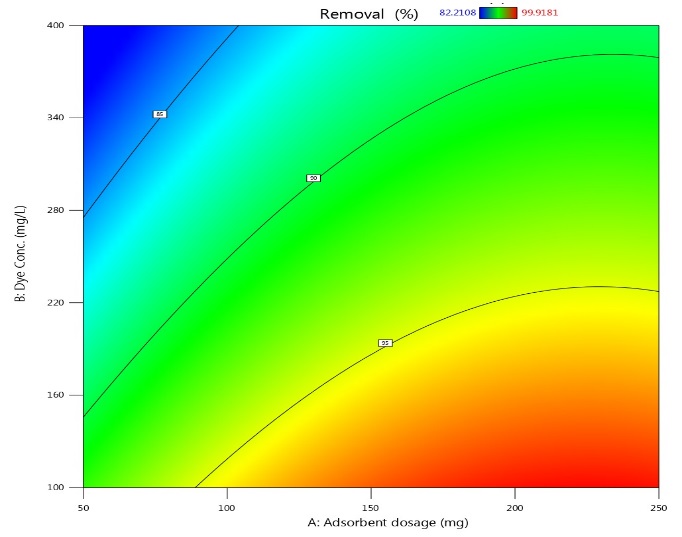 | 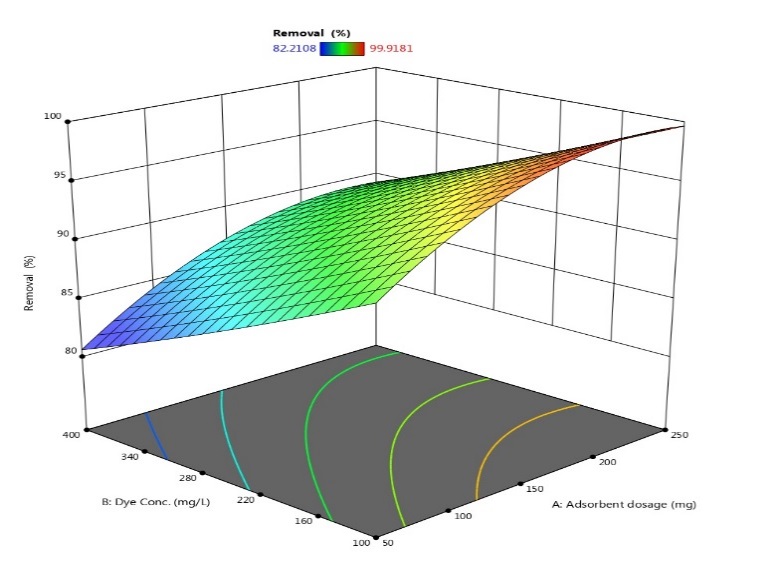 |
| --- | --- |
| (a) | |
| 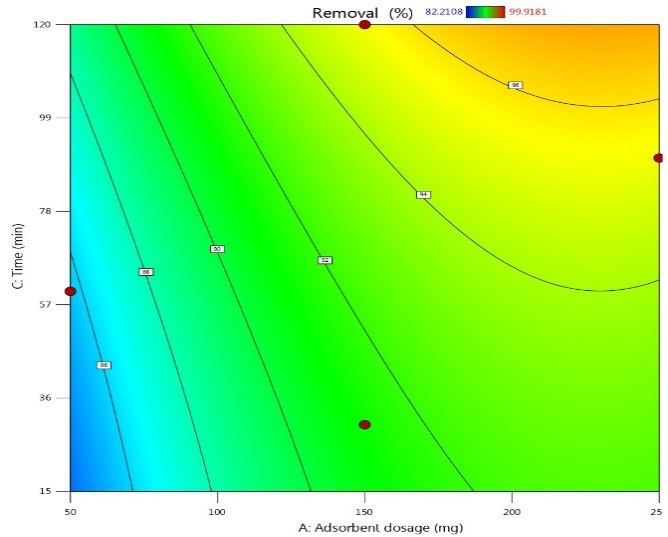 | 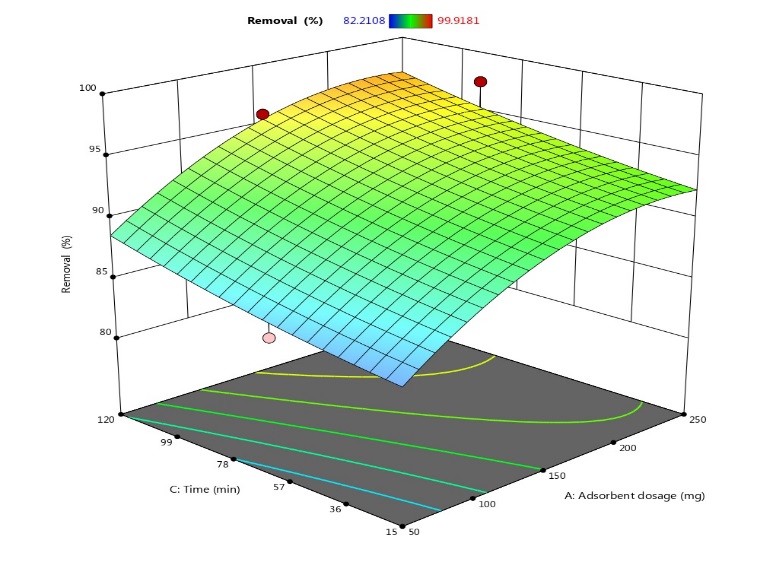 |
| (b) | |
| 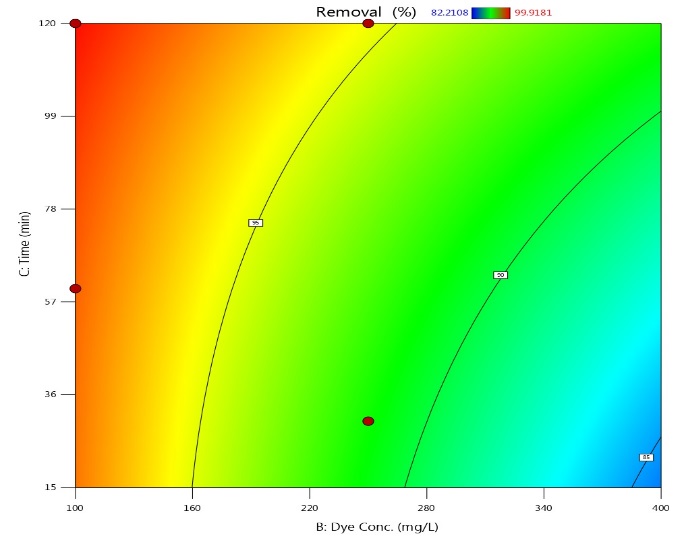 | 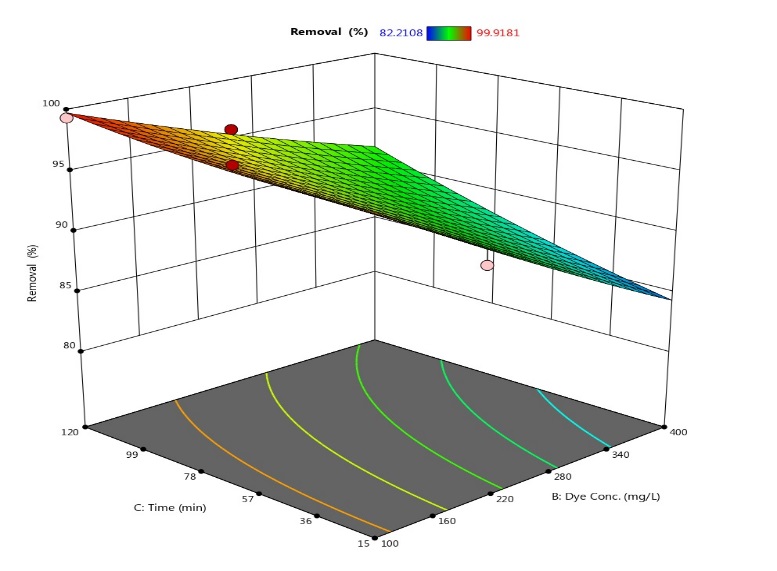 |
| (c) | |

**Figure S4.** The combined effect of process variables (**a**) adsorbent dosage and initial dye concentration, (**b**) adsorbent dosage and contact time and (**c**) initial dye concentration and contact time on AB14 dye removal with interaction effect of dual factors.

| 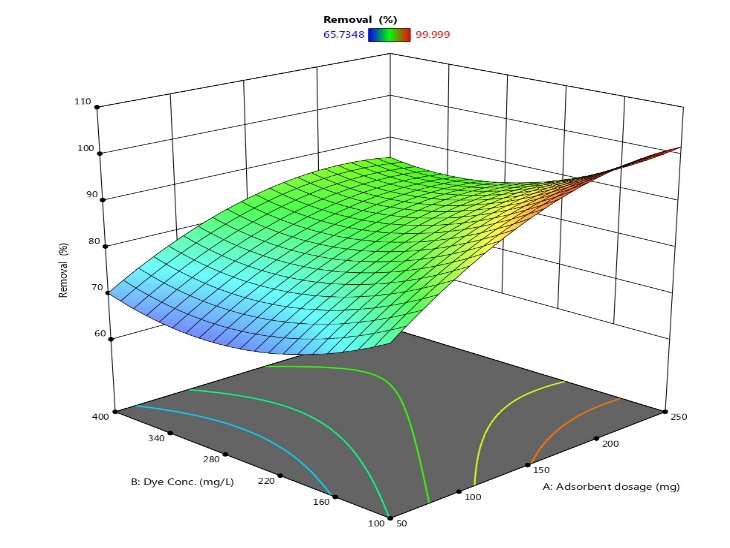 | 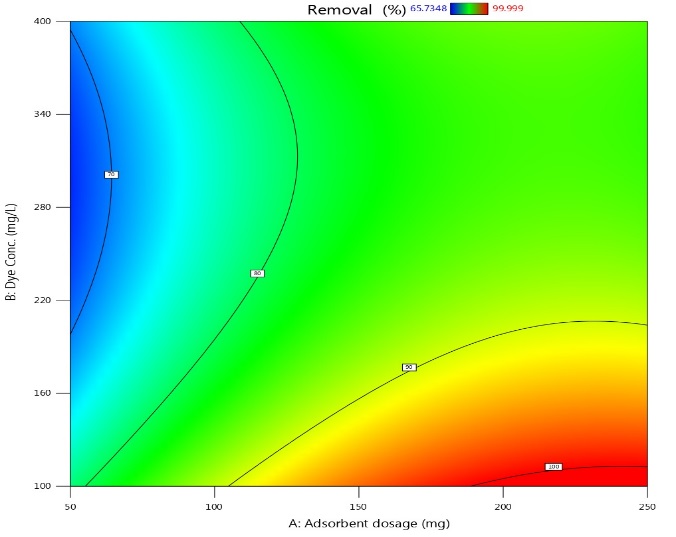 |
| --- | --- |
| (a) | |
| 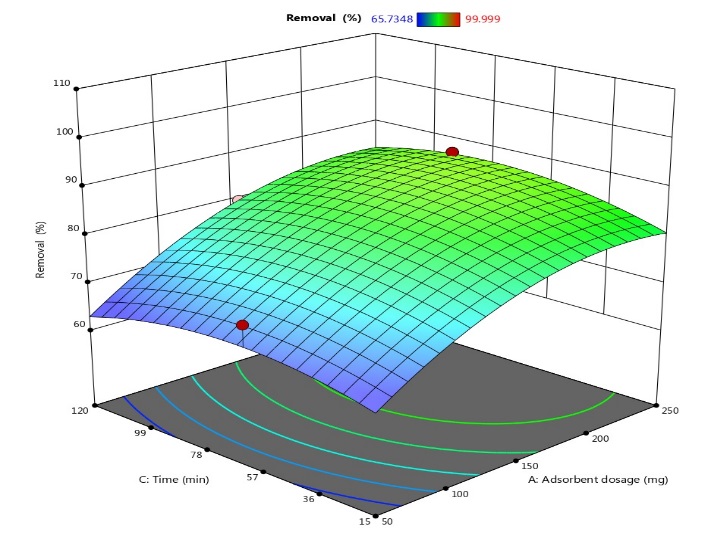 | 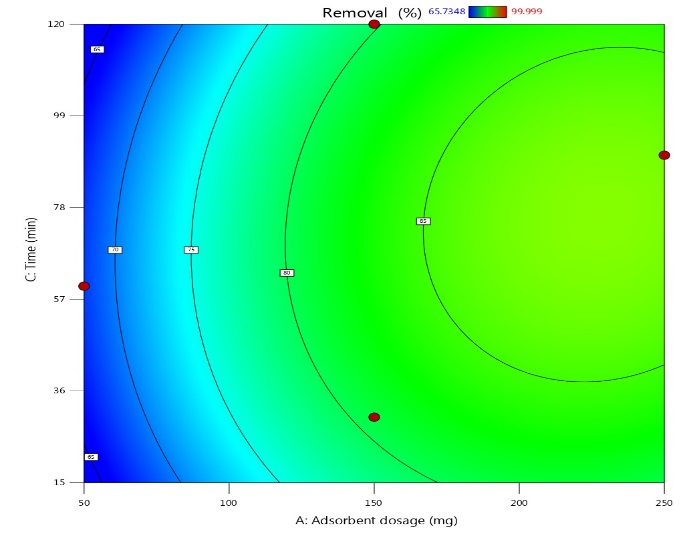 |
| (b) | |
| 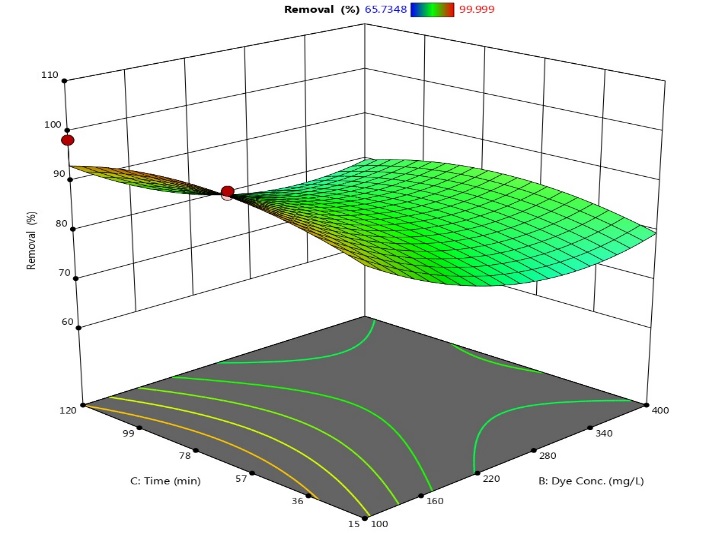 | 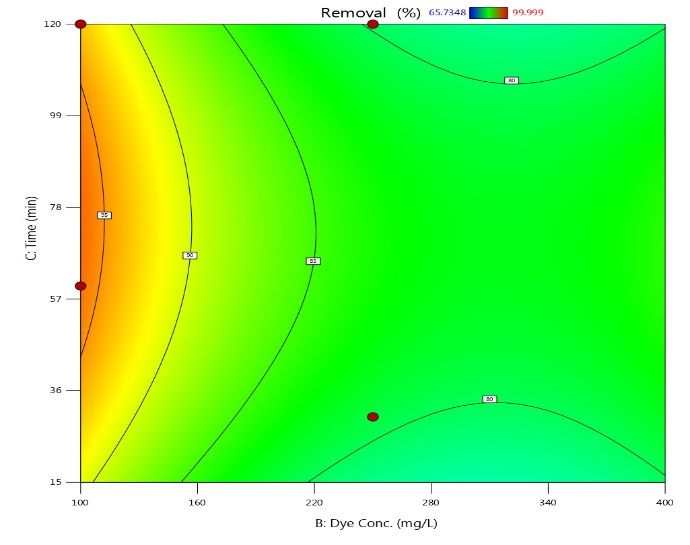 |
| (c) | |

**Figure S5.** The combined effect of process variables (**a**) adsorbent dosage and initial dye concentration, (**b**) adsorbent dosage and contact time and (**c**) initial dye concentration and contact time on AO7 dye removal with interaction effect of dual factors.

| 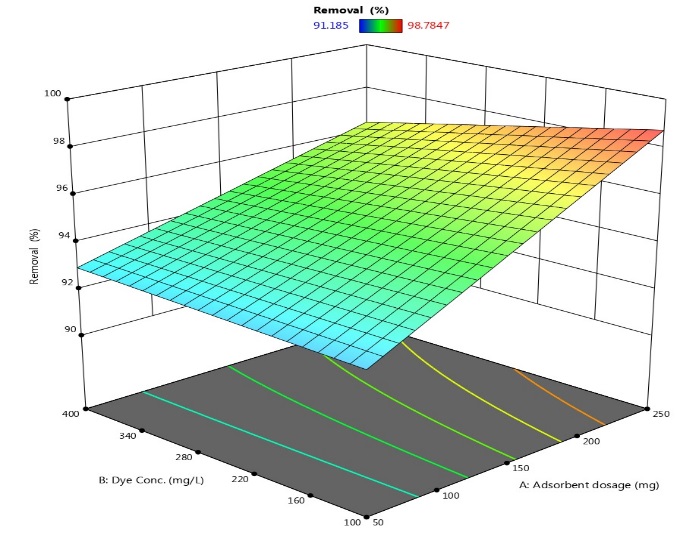 | 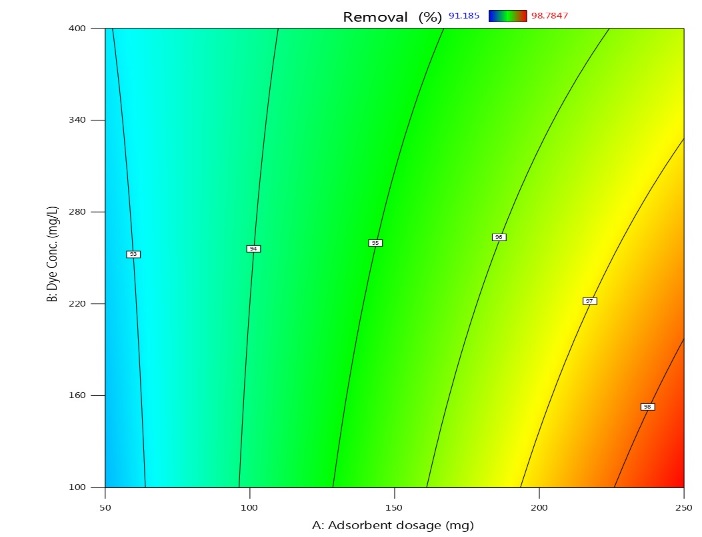 |
| --- | --- |
| (a) | |
| 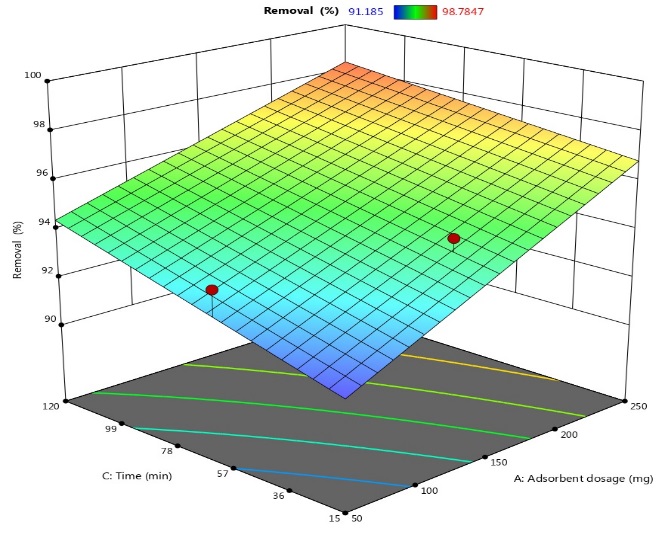 | 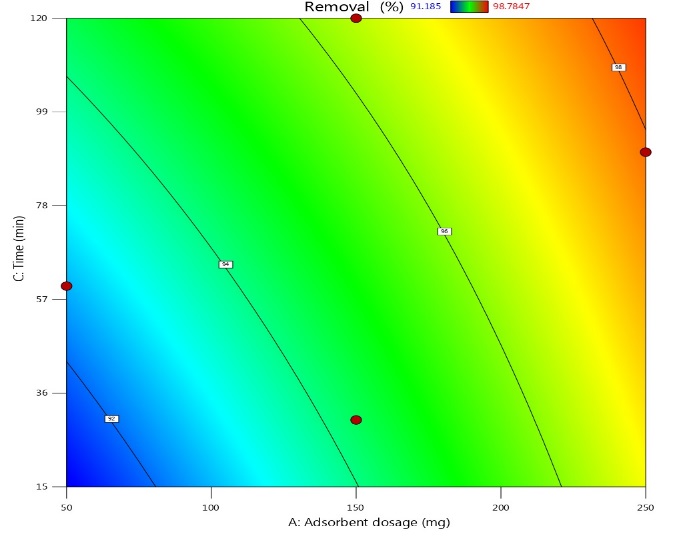 |
| (b) | |
| 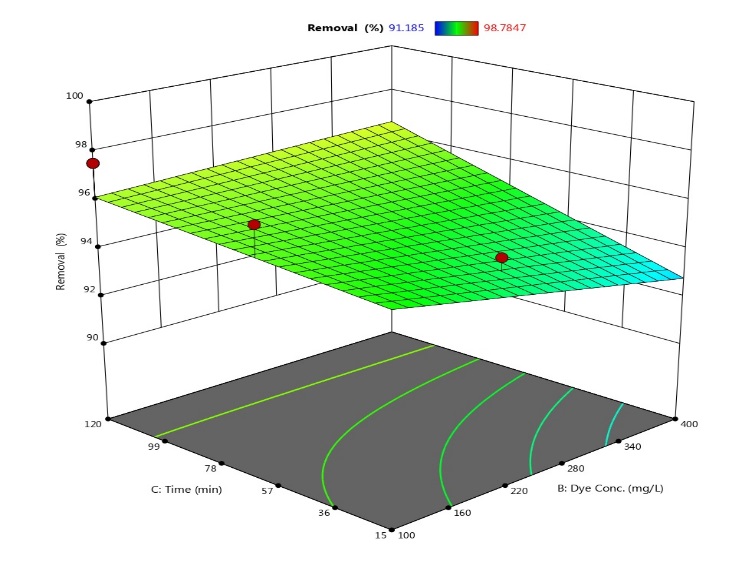 | 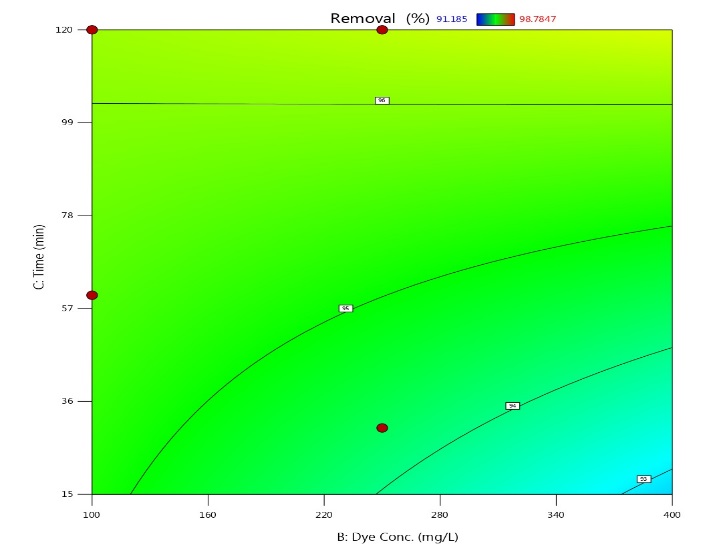 |
| (c) | |

**Figure S6.** The combined effect of process variables (**a**) adsorbent dosage and initial dye concentration, (**b**) adsorbent dosage and contact time and (**c**) initial dye concentration and contact time on Cr (VI) ions removal with interaction effect of dual factors.


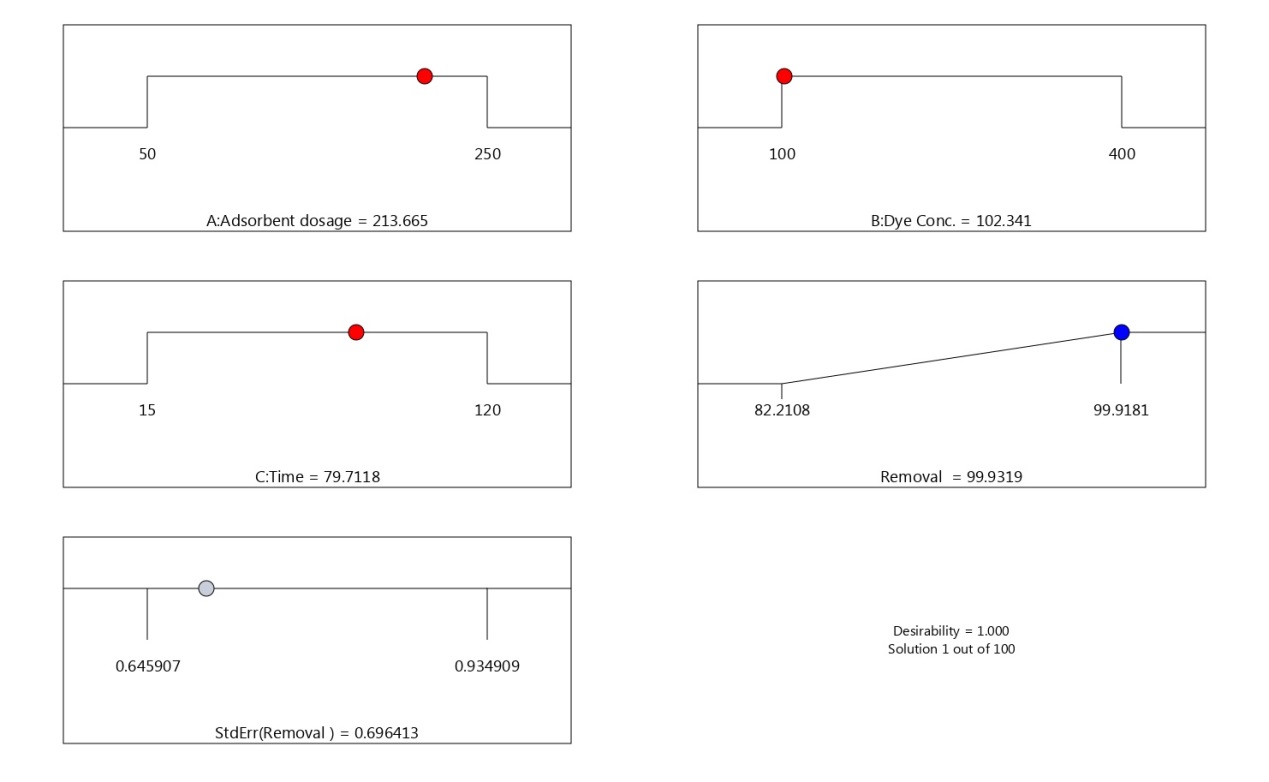


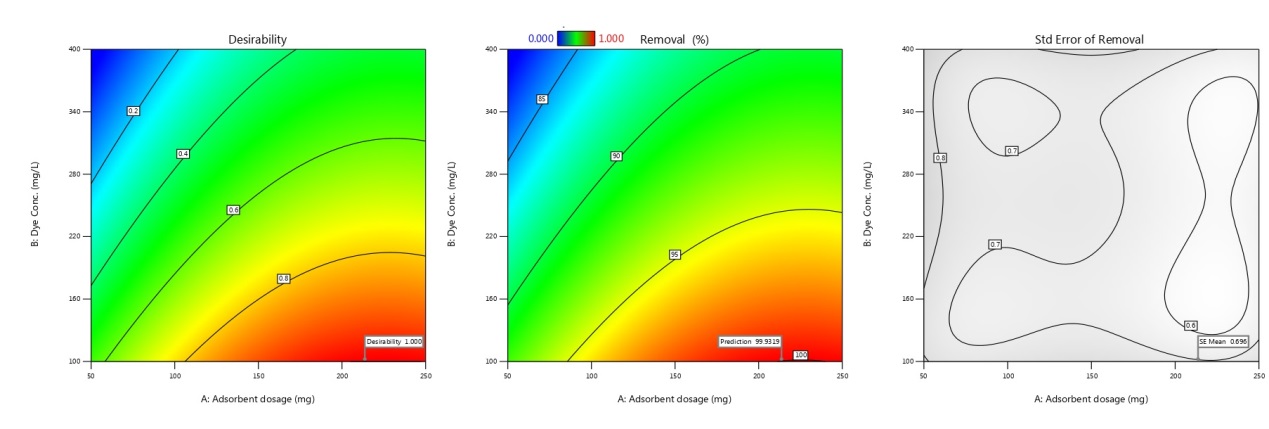


**Figure S7**. Optimum conditions predicted by RSM method for AB14 dye adsorption.


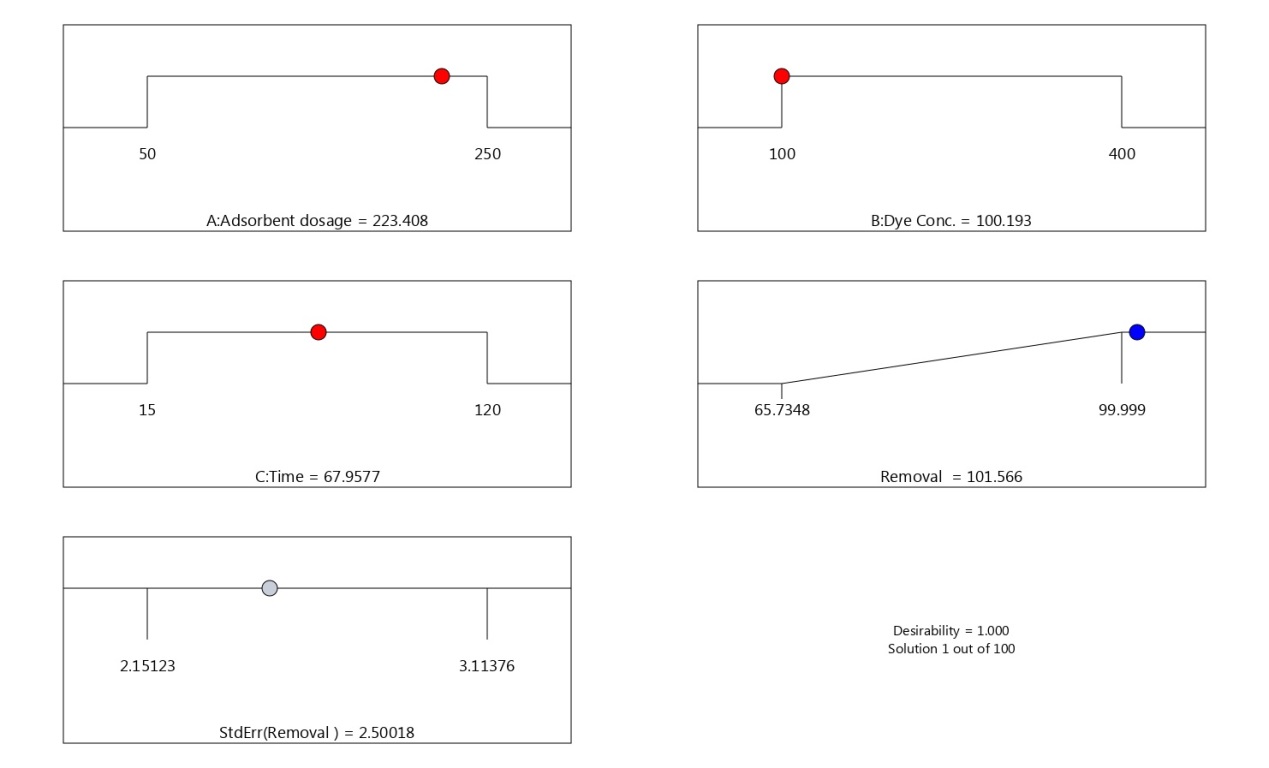


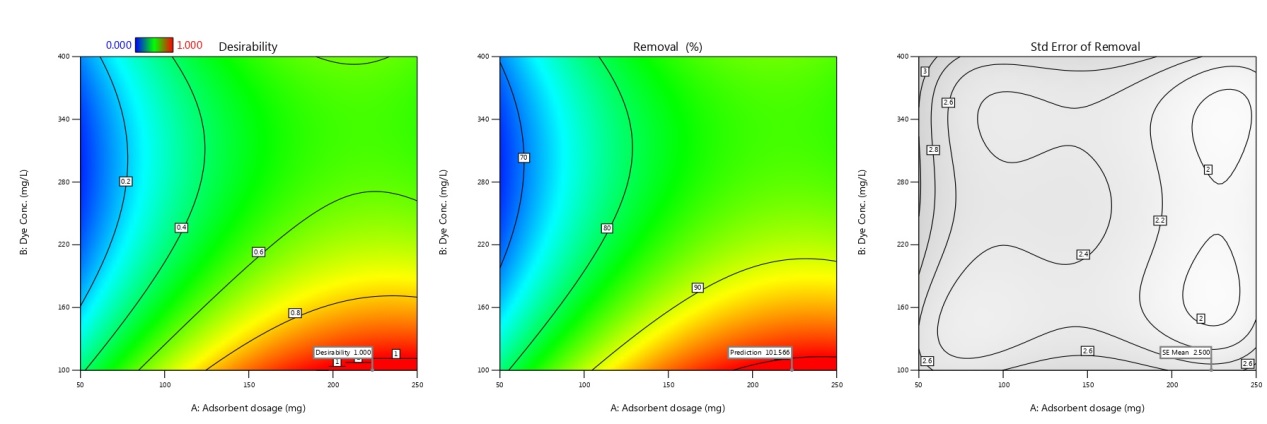


**Figure S8**. Optimum conditions predicted by RSM method for AO7 dye adsorption.


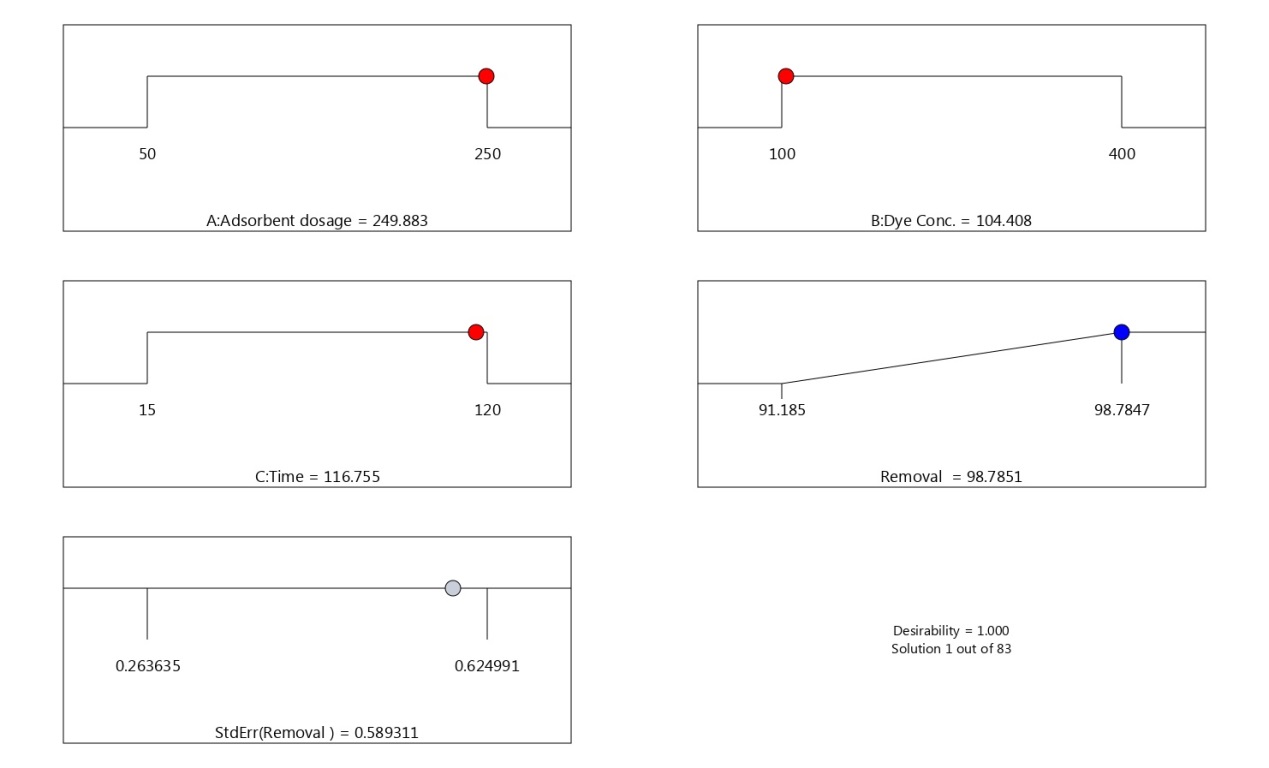


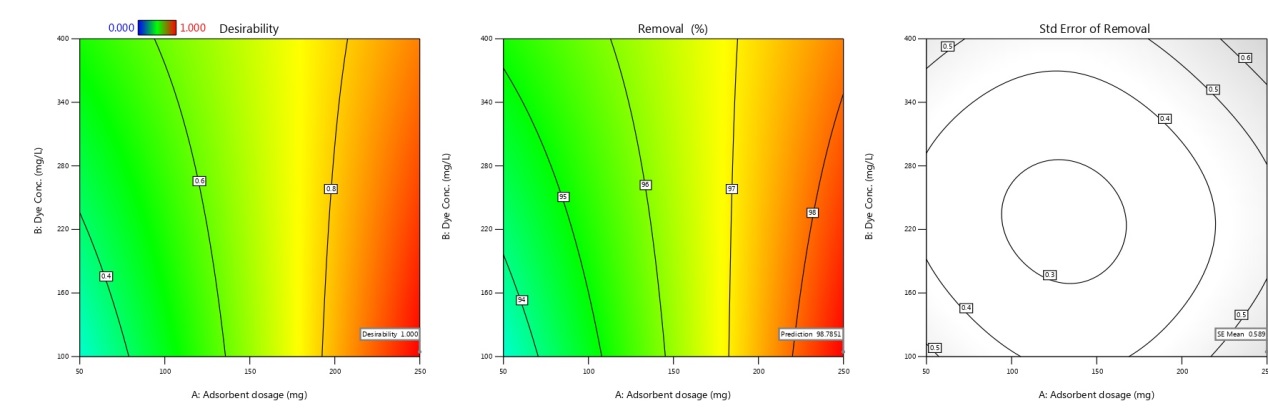


**Figure S9**. Optimum conditions predicted by the RSM method for Cr (VI) ions adsorption
